# Genome-Wide Identification, Characterization and Expression Analysis of Xyloglucan Endotransglucosylase/Hydrolase Genes Family in Barley (*Hordeum vulgare*)

**DOI:** 10.3390/molecules24101935

**Published:** 2019-05-20

**Authors:** Man-Man Fu, Chen Liu, Feibo Wu

**Affiliations:** 1 Department of Agronomy, College of Agriculture and Biotechnology, Zijingang Campus, Zhejiang University, Hangzhou 310058, China; sunshineabigail@163.com (M.-M.F.); liu-chen@zju.edu.cn (C.L.); 2 Jiangsu Co-Innovation Center for Modern Production Technology of Grain Crops, Yangzhou University, Yangzhou 225009, China

**Keywords:** Xyloglucan endotransglucosylase/hydrolase (XTH), genome-wide, gene expression, barley

## Abstract

Xyloglucan endotransglucosylase/hydrolases (XTHs)—a family of xyloglucan modifying enzymes—play an essential role in the construction and restructuring of xyloglucan cross-links. However, no comprehensive study has been performed on this gene family in barley. A total of 24 *HvXTH* genes (named *HvXTH1*-*24*) and an EG16 member were identified using the recently completed genomic database of barley (*Hordeum vulgare*). Phylogenetic analysis showed that 24 *HvXTH* genes could be classified into three phylogenetic groups: (I/II, III-A and III-B) and *HvXTH15* was in the ancestral group. All HvXTH protein members—except HvXTH15—had a conserved *N*-glycosylation site. The genomic location of *HvXTHs* on barley chromosomes showed that the 24 genes are unevenly distributed on the 7 chromosomes, with 10 of them specifically located on chromosome 7H. A structure-based sequence alignment demonstrates that each XTH possesses a highly conserved domain (ExDxE) responsible for catalytic activity. Expression profiles based on the barley genome database showed that *HvXTH* family members display different expression patterns in different tissues and at different stages. This study is the first systematic genomic analysis of the barley *HvXTH* gene family. Our results provide valuable information that will help to elucidate the roles of *HvXTH* genes in the growth and development of barley.

## 1. Introduction

Xyloglucan endotransglucosylase/hydrolases (XET/XEHs also named XTHs) are a family of xyloglucan modifying enzymes that have two different catalytic activities and can act either as endotransglucosylases (XET, EC 2.4.1.207) or as endohydrolases (XEH, EC 3.2.1.151). XTHs belong to glycoside hydrolase family 16 and play an important role in the construction and restructuring of xyloglucan cross-links [1]; their evolution has recently been studied. Faure et al. [2] found some enzymes, such as GH16 XETs and xyloglucanases, may have the ability to catalyze hetero-transglycosylation reactions in nasturtium (*Tropaeolum majus*) and the unique specificity of hetero-trans-β-glucanase (HTG) in horsetails evolved from XTH and is due to three key amino acid substitutions [3]. A previous analysis has indicated that XTHs are closely related to bacterial licheninases (EC 3.2.1.73), which specifically hydrolyze β(1→4) linkages [4]. A later study showed that a newly identified subfamily of GH16 endo-β-glucanases from plants are an evolutionary link between bacterial licheninases and XTH genes [5]. An enzymological characterization and X-ray crystallography of EG16 from *Vitis vinifera* have also given a crystallographic insight into the evolutionary origins of XTHs [6]. Genomic and transcriptomic analysis revealed that EG16 represents an early adoption of the β-jellyroll scaffold from an early GH16 member, which then, diversified by loop deletion and the extension of C terminus in XTHs [7].

The first systematic research of *XTH* gene family in *A. thaliana* showed that the *XTH* genes could be classified into three groups (I, II and III) [8,9]. A later study in rice (*Oryza sativa*) showed that there was no significant boundary between group I and II and the *XTH* genes in rice were classified into two major subgroups named I/II and III [10]. Based on the three-dimensional structures of Tm-NXG1 and Tm-NXG2 from nasturtium, XTHs in group III can be separated into two main clades (group III-A and group III-B) [11]. Furthermore, the study also gained a small outlier group (named ancestral group) close to the root of the tree. Detailed three-dimensional structures of Tm-NXG1 and Tm-NXG2 revealed the major structural features of the active site regulating relative rates of hydrolysis to transglycosylation in GH16 xyloglucan-active enzymes transformation, suggesting that XTH genes evolved to have a role in cell wall remodeling [11]. A study of *XTHs* in *Fragaria vesca* also suggested a similar classification of *XTH* genes and revealed differing expression levels of *XTHs* during different developmental stages of fruit ripening and in different tissues [12].

*XTH* genes play an active role in response to plant hormones. The expression of *OsXTH8* was upregulated by gibberellic acid (GAs) and there was little response to other hormones [13]. Members in the *XTH* gene family respond differently to different plant hormones and no close correlation was found between the tissue-specific expression and the responses to plant hormones of *XTH* genes in *A. thaliana* [8]. For example, the *XTH* genes—*AtXTH17*, *AtXTH18*, *AtXTH19* and *AtXTH20*—are phylogenetically closely related to each other and are preferentially expressed in roots but exhibit different expression profiles within roots and in response to hormonal signals [14]. Auxin and brassinosteroids (BR) can regulate a subset of *XTHs* during cell elongation [15]. AtXTH19 and AtXTH20 can be induced by ANAC071 (Arabidopsis NAC domain containing protein 71) in the distal part of an incised stem and were involved in cell proliferation in the tissue reunion process [16]. Expression of *DkXTH6* was up-regulated during ethylene production, as well as by propylene and abscisic acid (ABA) treatments but suppressed by GA3 and a cold treatment, while *DkXTH7* exhibited its highest transcript levels in GA3-treated fruit and cold-treated fruit [17].

With the development of Next Generation Sequencing (NGS), multigene families of XTHs have been reported both in monocotyledonous and dicotyledonous plants. There are 33 *XTH* family members in *Arabidopsis thaliana* [8], 29 in rice (*Oryza sativa*) [10], more than 57 in wheat (*Triticum aestivum*) [18], 35 in sorghum (*Sorghum bicolor*) [19], 56 in tobacco (*Nicotiana tabacum*) [20], 61 in soybean (*Glycine max*) [21], 21 in tomato (*Solanum lycopersicum*) [22,23], 41 in poplar (*Populus* spp.) [24]. Barley (*Hordeum vulgare*), one of the earliest domesticated species in the Near East, is the fourth most major cereal in the world [25]. Barley has been chosen for study, as it is an important crop species, as well as a model for genetic and physiological studies and breeding [26]. However, little is known about barley *XTHs*, except for the report that the expression of XET-related genes was related to elongation in leaves of barley [27] and can be heterologous expressed in *Pichia pastoris* [28]. Now the availability of barley genome and the comparative study of the *XTH* gene family across plant species provide an opportunity to explore the *XTH* diversity in barley [29,30]. In this study, we identified 24 candidate *HvXTHs* based on the bioinformatic analysis with the barley genome. Phylogenetic analysis indicated that the *HvXTHs* can be classified into three groups (I/II, III-A and III-B) and a small ancestral group. The structure and chromosomal location of *HvXTH* gene family members were also analyzed. Expression analysis showed that *HvXTH*s had different expression levels at different stages of development. *HvXTH8* showed high expression in all tissues except in senescing leaves. The transcripts of *HvXTH17* and *HvXTH18* in different tissues were at the similar level.

## 2. Results and Discussion

### 2.1. Identification of HvXTHs

With the publication of barley genome, it is possible to study the *XTH* family in barley on a genome-wide level. *EXT* (GenBank: X91659.1), reported as a candidate *HvXTH* of barley, was filtered in this study because of a lack of a complete ORF [24]. A total of 24 candidate *HvXTHs* were identified in the barley genome using data from EnsemblPlants. The details of information relating to previously reported *HvXTHs* is presented in Table 1 and renamed 24 predicted *HvXTHs* based on chromosome position. Previous research showed that the number of *XTHs* in commelinid monocots was approximately 30 homologs per genome with more than 30 *XTHs* being found in maize, wheat and rice [7]; fewer *XTH* members were identified in barley. Moreover, an EG16 homolog (Gene ID: HORVU4Hr1G085940.1, Gene locus: chr4H: 633,032,657-633,033,820) was also identified [7], which appears to be the evolutionary link between bacterial licheninases and *XTH* gene products [5,7].

### 2.2. Phylogenetic Analysis of HvXTHs

**Figure 1:**
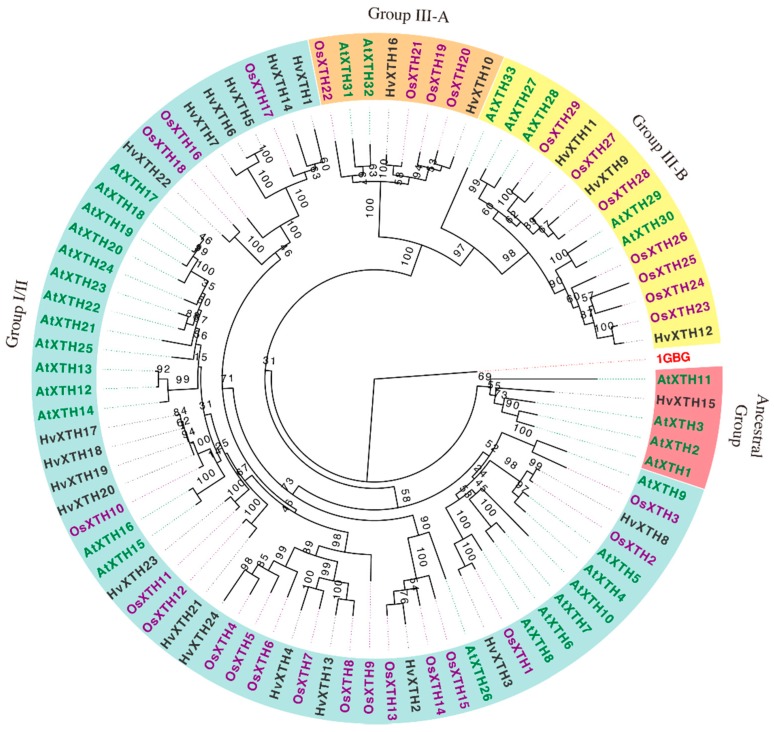
Phylogenetic tree of full-length HvXTH, OsXTH and AtXTH proteins. The tree was constructed using identified 24 *HvXTHs* in barley, 33 *AtXTHs* from Arabidopsis and 29 *OsXTHs* from rice. The numbers at each fork of the tree indicate the number of times (expressed as percentage) that the group of genes was clustered together in 100 bootstrap replicates.

A phylogenetic analysis was conducted with a total of 88 full-length protein sequences, using PhyML to study the evolutionary relationships of the *XTH* genes from barley (*HvXTH*), rice (*OsXTH*) and *A. thaliana* (*AtXTH*) (sequence information is provided in Supplementary File 1). The phylogenetic tree consists of three predominant branches with significant bootstrap support and used the structurally characterized bacterial lichenase (1GBG, EC 3.2.1.73) as an outgroup (Figure 1). *HvXTH* genes were classified into three groups: 18 genes in Group I/II, 2 in Group IIIA and 3 in Group IIIB. The ancestral group close to the root included *AtXTH1*, *AtXTH2*, *AtXTH3*, *AtXTH11* and *HvXTH15*; this structure is similar to found in previous research [11]. It was reported that the merging of group I and II constructed the largest branch [10]. There was no significant difference between group I and group II which constituted the largest cluster and is referred to as group I/II (containing 22 *AtXTHs*, 18 *OsXTHs* and 18 *HvXTHs*); this result is also consistent with a previous study [10]. Three *HvXTH* genes (*HvXTH9*, *HvXTH11* and *HvXTH12*) were classified into group III-B (including 5 *AtXTHs* and 7 *OsXTHs*) and *HvXTH15* was in the ancestral group; thus, most genes may exhibit XET activity. Two members (*HvXTH10* and *HvXTH16*) were in Group III-A and may have mixed glucosylase/hydrolases holding functions [11]. It seems that barley contains more *XTHs* in group I/II compared with rice. In dicotyledons, members of Group III have been shown to catalyze xyloglucan hydrolysis (xyloglucan endohydrolase activity) rather than xyloglucan transglucosylation (XET activity) [11,32,33]. A previous study of heterologous expression in the yeast *Pichia pastoris* [28] showed that HvXTH21 and HvXTH23 had detectable XET activity.

### 2.3. Gene Structure Analysis and Chromosomal location

**Figure 2:**
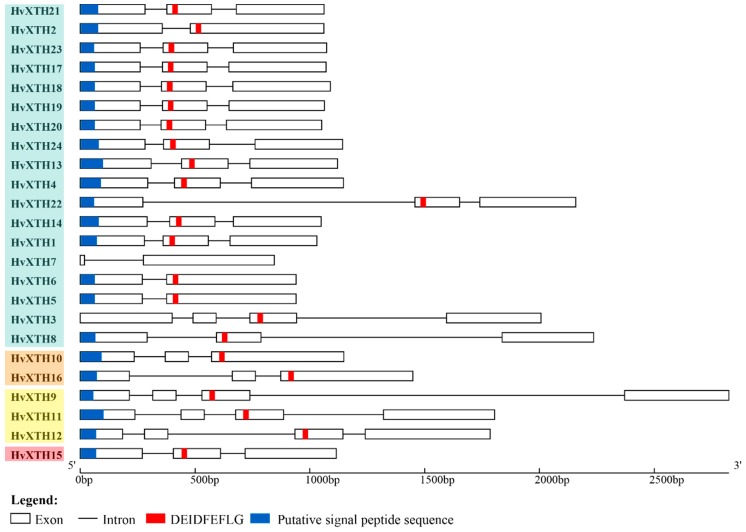
Gene structure and conserved protein domains of *XTH* genes in barley showing exons, introns, putative signal peptide sequences and motif sequence organization. Group I/II HvXTHs are in light blue, III/A in orange, III/B in yellow and the ancestral group in red. Sequence information is provided in Supplementary Files 3 and 4.

To obtain further insight into the structural diversity of the *XTH* genes in barley, the structure of the 24 *HvXTHs* (Figure 2) was identified using GSDS; coding and genomic sequences of *HvXTHs* are provided in Supplementary Files 2 and 3. The conserved motif sequence ExDxE was found in all HvXTH members (Figure 2). The number of exons varied from two to four which is similar to a previous study in rice [10]. The motif was always located on the second exon but also on the third exon of *XTH* (*HvXTH3*) in group I/II which is not consistent with study in *A. thaliana* [8,34]. SignalP was used to predict the presence of a signal peptide for entry into the secretory pathway for the 24 HvXTH proteins. The result showed that 22 HvXTH proteins contained putative signal peptides which were all located in the first exon (Figure 2).

**Figure 3:**
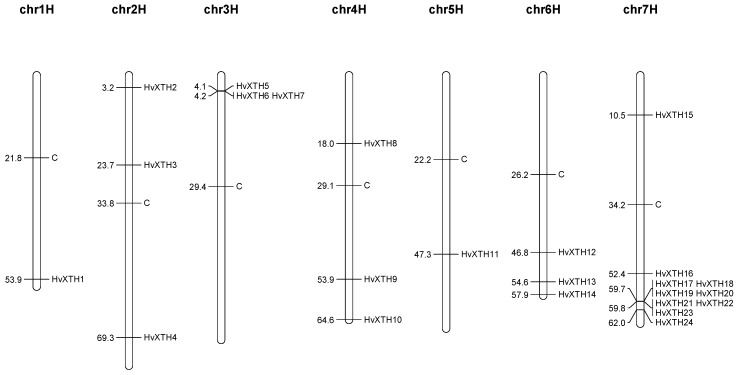
Distribution of *HvXTH* genes in barley chromosomes. The location of *HvXTH* gene family members in the genome barley cultivar ‘Morex’. C indicates the centromere.

The chromosomal positions of *HvXTH* gene family members were located using information derived from the barley genome [29]. A total of 24 *HvXTHs* were mapped to the genome (Figure 3). The *HvXTH* genes were unevenly distributed in the genome of the barley cultivar Morex. There was only one *HvXTH* gene located on chromosome 1H and chromosome 5H. Three *HvXTHs* could be found on each of chromosomes 2H, 3H, 4H, 6H. Chromosome 7H carried 10 *HvXTHs* which was the maximum number. Five or fewer genes located within 100 kb were usually considered as tandem duplicates [6]. In this study, four gene pairs (*HvXTH6/7*, *HvXTH17/18*, *HvXTH19/20*, *HvXTH21/22*) were detected within 100 kb on chromosome 3H and chromosome 7H, which may be the result of tandem duplication (Figure 3). The Smith-Waterman algorithm (http://www.ebi.ac.uk/Tools/psa/) suggested that the similarities of sequence between two pairs of genes (*HvXTH17/18*, *HvXTH19/20*) exceeded 90%. Taken together, these two pairs are considered as tandem duplicates. Detailed information is shown in Supplementary File 4. According to the phylogenetic tree, *HvXTH6* and *HvXTH7* and *HvXTH17*/*18*/*19*/*20* are closely related, which indicates that the gene duplications of these three pair of genes might be related to the evolution.

### 2.4. Structure-Based Sequence Alignment

**Figure 4:**
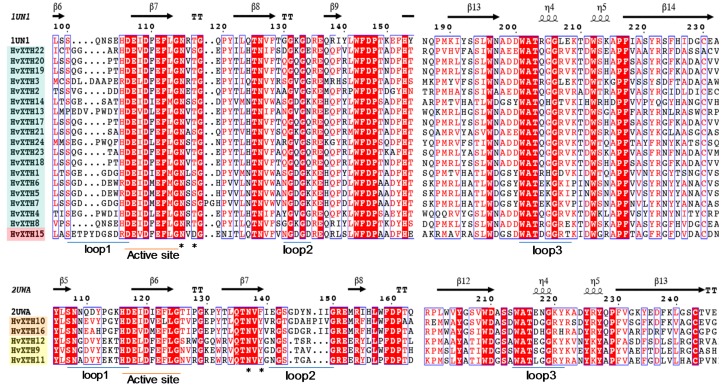
Structure-based sequence alignment of HvXTHs. Sequence were aligned using MEGA7 and secondary structures predicted using ESPript. HvXTHs in group I/II and group III had similar structures to PttXET16-34 (1UN1) and TmNXG1 (2UWA), respectively, whose structures have been experimentally determined. Group I/II HvXTHs are in light blue, group III/A in orange, group III/B in yellow, the ancestral group in red. Blue frames indicate conserved residues, white letters in red boxes indicate strict identity and red letters in white boxes indicate similarity. The secondary structures of β sheets (arrows) and α-helices (spiral) and loops 1, 2 and 3 (lines) are indicated. *N*-glycosylation residues are indicated as asterisks. The entire alignment is presented in Supplementary Files 5 and 6.

The alignment of the 24 HvXTHs, together with a xyloglucan endotransglycosylase—PttXET16-34 (PDB id: 1UN1) [35]—and a xyloglucan endohydrolase—TmNXG1 (PDB id: 2UWA) [11]—with known protein structures were used to predict the secondary structures of the HvXTH proteins of barley using ESPript (http://espript.ibcp.fr/ESPript/ESPript/). The HvXTHs in group I/II and group III had similar structures to PttXET16-34 and TmNXG1, respectively and the most conserved domains are shown in Figure 4. The entire alignment is provided in Supplementary Files 5 and 6. All the members of the HvXTH protein possessed the XET/XEH C-terminal extension, while the endo-glucanases (EG16 members), which are the evolutionary link between bacterial lichenases and XTHs, normally lack this domain [5,6].

The active site (**E**xDx**E)** containing the residues responsible for catalytic activity was highly conserved in all of the HvXTH family members. The first glutamate residue (E) acts as catalytic nucleophile which initiates the enzymatic reaction and the second one as a base to activate the substrate. All members of HvXTH family, except HvXTH15 (ancestral group), had a conserved *N*-glycosylation site (marked with asterisks), which is proximal to the catalytic residues as reported in *Fragaria vesca* [12]. The *N*-glycosylation site domain (NxT/S/Y) is thought to play a role in protein stability. Heterologous expression in *Pichia pastoris* produced some mannosylated protein glycoforms, which did not significantly affect enzymic activity [36].

Group IIIA had longer Loop 2 than the other groups and mainly showed xyloglucan hydrolase activity and previous structural and biochemical analysis of TmNXG indicated that the extension of loop 2 was a key factor in determining endo-xyloglucanase activity in XTH gene products [11]. There were no Ser-213 and Asn (Glu)-218 substitutions in group III-A; this result differs from the study in nasturtium, in which the substitutions were found in most sequences of group IIIA [11]. Members of group III-B (HvXTH9, HvXTH11 to HvXTH12) containing Ser (Ala)-213 substitutions and three residues are missing in loop3 which indicates they might have endotransglucosylase as their major enzyme activity [5,6,7]. The alignment of the EG16 protein encoded by HORVU4Hr1G085940.1 (Gene locus: chr4H: 633,032,657-633,033,820) with other representative EG16s from poplar, grape and selected monocot EG16s was also performed [5,6,7]. The analysis showed that EG16 and HvXTH share the same catalytic site. The full alignment is provided in Supplementary File 7.

### 2.5. Expression Patterns of HvXTHs at Different Developmental Stages and Tissues

**Figure 5:**
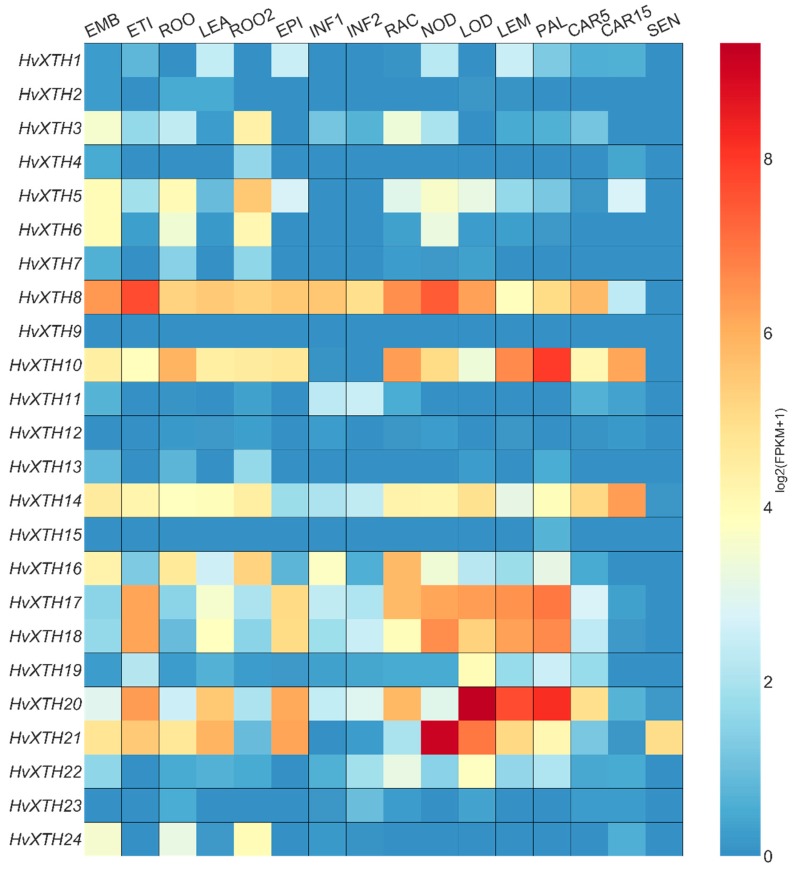
Heatmap of *HvXTH* gene expression in different tissues and stages of development. Data were obtained from a publicly available database, ENA (Accession number: PRJUEB3149, PRJEB14349). Rows represent *HvXTH* members, while columns show different developmental stages and tissues. The expression level of *HvXTHs* (log_2_(FPKM + 1)) is shown by the intensity of color. EMB, embryonic tissue; ETI, isolate etiolated leaf; ROO, root tissue (17 days and 28 days after planting); LEA, leaf tissue; EPI, epidermal strips; INF, whole developing inflorescence tissue (30 days and 50 days after planting); RAC, rachis; NOD, third stem internode; LOD, isolate lodicule dissected from inflorescences; LEM, lemma dissected from inflorescences; PAL, palea dissected inflorescences; CAR, whole developing grain (caryopsis, 5 and 15 days post-anthesis); SEN, senescing leaf. Detailed information is presented in Supplementary File 8 and Table S2.

Transcript analysis showed that members of the *HvXTH* gene family had distinctly different expression patterns during different developmental stages as well as in different plant tissues (Figure 5; Supplementary File 8: Table S2). Some members of *HvXTH* (such as *HvXTH8*, *HvXTH10*, *HvXTH14*, *HvXTH17*, *HvXTH18*, *HvXTH20* and *HvXTH21*) were expressed in almost all of the sixteen stages or tissue analyzed in the present study. Among them, *HvXTH8* showed high expression in all tissues, especially in leaf tissue (ETI) and the third stem internode (NOD). *HvXTH10* is mainly expressed in the rachis (RAC), lemma (LEM) and palea (PAL). The transcript level of *HvXTH14* in caryopsis (CAR15) was higher than in other tissues. The expression pattern of *HvXTH17* and *HvXTH18* showed a high degree of similarity which indicates that they may play similar roles in the development of the plant. *HvXTH20* showed a higher expression level in the lodicule (LOD), LEM and PAL compared with other tissues. *HvXTH21* was highly expressed in NOD and LOD. While *HvXTH2*, *HvXTH4*, *HvXTH9*, *HvXTH12*, *HvXTH15* and *HvXTH23* showed lower expression in almost all developmental stages and tissues. Other members of the *HvXTH* family showed tissue-specific expression patterns. For example, the expression of *HvXTH24* was only obviously detected in etiolated leaves (ETI) and roots (ROO2). *HvXTH6* was only expressed in embryos (EMB), ROO, ROO2 and NOD. The transcript of *HvXTH7* was mainly expressed in roots (ROO and ROO2). *HvXTH11* was almost only expressed in the developing inflorescence tissues, INF1 and INF2, which indicates that *HvXTH11* might be involved in the inflorescence development. Both *HvXTH19* and *HvXTH22* showed a higher expression level in inflorescence tissues such as LOD, LEM and PAL.

Previous research has reported that only a few genes in the *XTH* family have been verified as encoding true XTH proteins [37,38]. Domain and structural analysis, together with the RNA-seq data, strongly indicated that these genes encode proteins. Previous research on *AtXTH* expression using GUS staining in *Arabidopsis* showed that *AtXTHs* are probably expressed in all developmental stages from seed germination through to flowering [39]. Consistent with these results, *HvXTH* family members play different roles in plant development. About half of the *HvXTH* family members showed higher expression in embryonic tissue from germinating grains (EMB), which indicates that *HvXTHs* might play an active role in seed germination and Aubert et al. showed that *AtXTH31* is predicted to play a role in seed germination [40]. Almost all members of the *HvXTH* family are expressed in roots and leaves. Previous research has shown that *AtXTH31* might be involved in root morphogenesis [39,40] and that *AtXTH32* is primarily expressed in the shoot apex [39]. The location of *LeEXT* in epidermal cells in the elongating region suggests a role in elongation induced by auxin [41]. *HvXTH* might also play an essential role in this process.

Internode extension is related to plant height which is an essential element for crop breeding. Gene expression analysis showed that *HvXTH21* has the highest expression in the third stem internode compared with other tissues as well as in comparison with all other members of the *HvXTH* family. These results indicate that members of *HvXTH* family might affect the growth of barley through affecting stem extension. *HvXTH20* shows the highest expression in LOD, LEM and PAL (which are significant components of inflorescence tissues) compared with other tissues. This indicates that higher expression of *HvXTH20* may be beneficial for the development of inflorescence tissues (such as the extension of the rachis and lodicule) which might play an important role in seed formation. XTH activity in vegetative tissues was examined and showed that XTHs might be involved in stopping elongation, which indicates that the activity of XTHs is not always related to plant growth [42,43,44]. Previous study reported that the linkages between the G-layer and secondary cell walls are XET-dependent and have been implicated in transmitting tensile stresses [44,45].

Two grain developmental stages were used to analyze the expression patterns of the 24 *HvXTH* family members. The result indicates that the expression of *HvXTH8*, *HvXTH10*, *HvXTH14*. *HvXTH20* might play a role in the development of barley grain. The various expression patterns of *HvXTH* family members in different developmental stages indicated that *HvXTH* genes play an active role in the development of barley [44].

## 3. Conclusions

In conclusion, 24 *HvXTH* genes (named as *HvXTH1*-*24*) were identified and could be divided into three phylogenetic groups. Similar to other Poales [7], one EG16 gene was also found. Gene location analysis showed that *HvXTHs* are distributed unevenly on 7 barley chromosomes. *HvXTH17/18* and *HvXTH19/20* gene pairs may be tandem duplicates. Structure-based alignment showed that all HvXTHs contain conserved domains and *N*-glycosylation sites. The high expression of *HvXTH8*, *HvXTH10*, *HvXTH14*, *HvXTH17*, *HvXTH18*, *HvXTH20* and *HvXTH21* in all 16 tissues during different developmental stages suggests that *HvXTHs* might have different functions and be involved in the plant development. Taken together, based on a genome-wide identification and analysis of the *HvXTH* gene family in barley, our research provides a foundation for further functional research of *HvXTHs* in barley development.

## 4. Materials and Methods

### 4.1. Identification of XTH Family Genes in H. vulgare L.

Gene, cDNA and protein sequences were retrieved from the Ensembl Plants database (http://plants.ensembl.org) [46]. Two methods were used to identify barley XTH proteins in this study. Firstly, the Hidden Markov Model (HMM) was used to search the protein database using HMMER3.0 with default parameters in which the profiles of the XTH protein domains, PF00722 and PF06955, were used as queries. The online program SMART (http://smart.embl-heidelberg.de/) [47] was used to identify conserved domains of candidate barley XTHs. Only the proteins containing both domains were kept for further analysis. The second method was based on the BLAST analysis of homologous proteins. Twenty-nine published OsXTH protein sequences [10] were used as queries to map the *H. vulgare* genome database using the parameters E-value < 10^−15^ and identity >50% and redundant sequences were removed manually. Then, the remaining candidate XTH protein sequences with conserved PF00722 and PF06955 domains were filtered by the SMART tool using an E-value of <0.1. After that, the gene structure was checked according to information of IPK and sequences published in NCBI. Some ORFs were rejected because they contained uncharacteristic amino acid sequences located in the predicted C-terminal regions or lacked the signature motif that includes the residues responsible for the catalytic activity and is conserved among all the XTHs thus far characterized [8,34]. In these cases, the coding regions of these predicted genes were manually reinterpreted based both on the conserved structural features of XTHs and on the sequence database of full-length cDNAs. Some annotation of *HvXTH* CDS sequences was not consistent with the NCBI BLAST analysis, so we compared the data in IPK and NCBI and reannotated them. Finally, XTH proteins of barley were identified based on methods described above and used for further analysis. The names of barley *XTH* genes were nominated according to the previous study [34].

### 4.2. Phylogenetic Analysis of HvXTH Genes

Phylogenetic analysis was performed using the deduced amino acid sequences obtained with identified *HvXTHs* together with 29 *OsXTH* genes and 33 *AtXTH* genes; the structurally characterized bacterial lichenase, 1GBG; was used as an outgroup. After removing the predicted signal peptide, alignment analysis of amino acid sequences was performed using MAFFT with the iterative refinement method and the scoring matrix Blosum62 [48]. Then the obtained alignment was manually refined by Bioedit (http://www.mbio.ncsu.edu/bioedit/bioedit.html), based on the crystal structures of Ptt-XET16-34 and Tm-NXG1. A phylogenetic tree was constructed using maximum likelihood (ML) by PhyML [49]. The percentages of trees in which the associated taxa clustered together were determined from 100 bootstrap replications.

### 4.3. Gene Structure Analysis and Chromosomal Location

The Gene Structure Display Server (GSDS) tool (http://gsds.cbi.pku.edu.cn/) [50] was employed to identify exon/intron organization of the *HvXTH* genes by aligning the CDS sequences with the corresponding genomic DNA sequences. Putative signal peptide sequences were predicted using the online website SignalP (http://www.cbs.dtu.dk/services/SignalP/) [51]. The MEME tool (http://meme-suite.org/tools/meme) [52] was used to predict the DEIDFEFLG motif. The chromosomal locations of the *HvXTH* genes were physically mapped to each chromosome according to their positions in the barley genome. The physical map was depicted using MapChart tools (v2.3.2) [53].

### 4.4. Structural-Based Sequence Alignment

The online tool, ESPript (http://espript.ibcp.fr/ESPript/ESPript/) [54], was used to predict the secondary structures as well as the presence of structural elements in the HvXTH protein sequences. Alignment of the candidate HvXTH sequences was conducted by ClustalW with default settings that aimed to identify common structural elements of XTHs in barley. The crystal structure of TmNXG1 (PDB id: 2UWA) [11] and that of PttXET16-34 (PDB id:1UN1) [35] were obtained from the PDB databank to locate secondary structures.

### 4.5. Gene Expression Analysis

RNA-Seq data of 16 developmental stages published previously was downloaded from the ENA (Accession number: PRJUEB3149, PRJEB14349) database to analyze the different expression patterns of *HvXTHs* [29]. The data filtered by Trimmomatic were mapped to the genome using TopHat (v2.1.1) [55] with the default settings. Cufflinks (v2.2.1) was used to predict expression level of genes and is presented as fragments per kilobase of exon per million fragments mapped (FPKM) (parameters: -G -b –compatible-hits-norm –F 0.00001). Then, the expression levels of the transcript were calculated by Cuffdiff (parameters: --upper-quartile-norm, --frag-bias-correct). Finally, the expression levels of *HvXTHs* was normalized using log_10_ (FPKM + 1). The developmental stages analyzed were: embryonic tissue (EMB); isolate etiolated leaves (ETI); root tissue (17 days and 28 days after planting) (ROO, ROO2); leaf tissue (LEA); epidermal strips (EPI); whole developing inflorescence tissue (30 days and 50 days after planting) (INF1, INF2); rachis (RAC); third stem internodes (NOD); isolated lodicules dissected from inflorescences (LOD); lemma dissected from inflorescences (LEM); palea dissected from inflorescences (PAL); whole developing grain (caryopsis, 5 and 15 days post-anthesis) (CAR5, CAR15); senescing leaves (SEN).

## Figures and Tables

**Table 1:** *XTH* genes identified in barley genome.

Gene Name	Previous Gene Name	Gene ID	UniProtKB/TrEMBL	Chromosome
*HvXTH1*	*HvPM5* [27]	HORVU1Hr1G087320.1	-	chr 1H
*HvXTH2*		HORVU2Hr1G014530.3	A0A287H550	chr 2H
*HvXTH3*		HORVU2Hr1G045330.4	M0W9B4	chr 2H
*HvXTH4*		HORVU2Hr1G101160.2	M0YQG6	chr 2H
*HvXTH5*		HORVU3Hr1G016800.1	F2D9R7	chr 3H
*HvXTH6*		HORVU3Hr1G016820.3	-	chr 3H
*HvXTH7*		HORVU3Hr1G016850.3	A0A287K963	chr 3H
*HvXTH8*		HORVU4Hr1G028720.1	A0A287NP77	chr 4H
*HvXTH9*		HORVU4Hr1G064220.1	A0A287PD07	chr 4H
*HvXTH10*	*XET7* [28]	HORVU4Hr1G090820.2	F2DPR1	chr 4H
*HvXTH11*		HORVU5Hr1G060340.3	A0A287RFA0	chr 5H
*HvXTH12*		HORVU6Hr1G067470.2	A0A287UI99	chr 6H
*HvXTH13*		HORVU6Hr1G081590.1	A0A287UX29	chr 6H
*HvXTH14*	*HvPM2* [27]	HORVU6Hr1G093230.2	F2D903	chr 6H
*HvXTH15*		HORVU7Hr1G039600.2	A0A287W560	chr 7H
*HvXTH16*		HORVU7Hr1G086890.3	M0XPV0	chr 7H
*HvXTH17*		HORVU7Hr1G098260.1	F2D1T0	chr 7H
*HvXTH18*		HORVU7Hr1G098280.1	A0A287XI65	chr 7H
*HvXTH19*		HORVU7Hr1G098320.3	A0A287XI88	chr 7H
*HvXTH20*		HORVU7Hr1G098330.4	-	chr 7H
*HvXTH21*	*HvXET6* [31]	HORVU7Hr1G098370.1	F2DM52	chr 7H
*HvXTH22*		HORVU7Hr1G098390.1	F2D337	chr 7H
*HvXTH23*	*HvXEB* [27]	HORVU7Hr1G098440.2	F2DUU5	chr 7H
*HvXTH24*		HORVU7Hr1G106530.2	-	chr 7H

## Supplementary Materials

The following are available online. Supplementary File 1: Protein sequences of XTHs employed in the phylogenetic analysis (DOCX 29 kb); Supplementary File 2: Coding sequences of *HvXTHs* (DOCX 21 kb); Supplementary File 3: Genomic sequences of *HvXTHs* (DOCX 27 kb); Supplementary File 4: Table S1. Pairwise identities between paralogous pairs of *HvXTH* genes (DOCX 16 kb); Supplementary File 5: The multiple alignment of deduced amino acid sequences of HvXTHs 22-28and 2UWA (PDF 23 kb); Supplementary File 6: The multiple alignment of deduced amino acid sequences of HvXTHs and 2UWA 1-21 (PDF 28 kb); Supplementary File 7: The multiple alignment of deduced amino acid sequences of HvXTHs and EG16(PDF 28kb); Supplementary File 8: Table S2. Plant tissues for RNA-seq (DOCX 18 kb).
