# Supplementary material for: Genome-Wide Identification, Characterization and Expression Analysis of Xyloglucan Endotransglucosylase/Hydrolase Genes Family in Barley (Hordeum vulgare)
Source: Molecules. 2019 May 20;24(10):1935. doi: 10.3390/molecules24101935 (PMC6572274; doi:10.3390/molecules24101935)
Supplement: Supplementary file 1 [file molecules-24-01935-s001.zip › Supplementary File 2 Coding sequences of HvXTHs.docx]

**Supplementary File S5: Coding sequences of *HvXTHs***

>HvXTH22

ATGCTGCGCGGCTCCCTCCGGTGGCTGCTGGTGCTGGCCGTGGTGGTGGCGGCGTCCGCCGGGAAGGCCGGCCGGGGCCTGCACCGGGACTTCGACGCCGTGTGGGGGAAGCGCAATGCGCGCTTCTTCGACGAGGGCCGGGTGGTGGAGCTGGCGCTGGACCGGGAGACGGGGTCCAGGCTGCAGTCCAAGGACCGGTACCTCTTCGGGCGGTTCGACCTCGACATCAGGCTCGTCGCCGGCGAGTCCGCCGGGACCATCACCTCCTTCTACATCTGCACGGGTGGCGCGCGGCACGACGAGGTGGACTTCGAGTTCCTGGGCAACGTGAGCGGCGAGCCCTACATCCTGCACACCAACATCTTCAGCGACGGCAAGGGCGAGCGGGAGCAGCAGTTCGTGCTCTGGTTCGACCCCACCGCCGACTTCCACACCTACTCCATCCTCTGGAACCCGCTCAACATCATCCTGTACATCGACGGGACGCCGATCAGGGTGTTCAAGAACAACGAGGCCAACGGGGTGCCGTTCCCGACGAGGCAGCCGGTGCACGTCTTCGCCAGCATCTGGAACGCCGAGGAGTGGGCGACGCAGGGCGGCCGCGTCAAGACGGACTGGTCGGAGGCGCCGTTCGTGGCCGCGTACCGGCGCTTCGACGCCAGCAGCGCCTGCGTCTGGCATGGCGGGGCGTCGCCGACGCGGTGCGGCGGCGACCACCTGCCGTCGTCGGCGTCGTCGTGGATGGGGCAGCGGCTGGACTGGTGGAGCTGGATGACGCTCAACTGGGTGCGCATGAACTACATGACCTACGACTACTGCGCCGACCGGAAGCGGTACCCCCACGGGTTCCCCGCCGAGTGCATCATCCCCATCGGGAGGATCTGA

>HvXTH20

ATGGCTCGCATGGCGGTGTCGGTGCTGGCGATCCTGCTCGCCTGGTGCGCCCTGGCGGCGGCGAGCTTCGACAAGGAGTTCGACATCACCTGGGGCGACGGGCGTGGCAAGATCCTGAACAATGGTCAGCTCCTGACGCTGGCGCTGGACAAGGTGTCCGGCTCCGGGTTCCAGTCCAAGCACGAGTACCTCTTCGGCAAGATCGACATGCAGCTCAAGCTCGTCCCCGGCAACTCTGCCGGCACCGTCACCGCATACTACCTGTCGTCGCAGGGGCCGACGCACGACGAGATCGACTTCGAGTTCCTGGGCAACGTCACCGGCGAGCCCTACACGCTGCACACCAACGTGTTCACGCAGGGGCAAGGCCAACGGGAGCAGCAGTTTCGCCTCTGGTTCGATCCCACCAACGATTTCCACACCTACTCCATCCTCTGGAACCCAAAGCACATCATCTTCATGGTGGACGACATGCCGATCAGAGACTTCAAGAACCTGGAGGGAAAAGGGATCGCCTTCCCCAAGAACCAGCCCATGCGGCTCTACTCCAGCCTCTGGAACGCCGACGACTGGGCAACGCAGGGCGGCCGCGTCAAGACGGACTGGTCCCACGCCCCGTTCTCGGCCTCCTATCGCGGCTTCAAGGCAGACGCGTGCGTGGTGACCGCGGGCGGGCGGCCTCACTGCGGCGCCAGCGTCGGCACGGACGTCGCGCCCGGCACAGGCGCGGCCGGCGAGTGGTACAACCAGGAGCTGGACCTGACGCGGCAGCAGCGGATGCGGTGGGTACAGAGCAACTACATGATCTACAACTACTGCACCGACCCCAAGAGGTTCGCTCAGGGCGTCCCCGCCGAGTGCTCCATGTAG

>HvXTH16

ATGGCCAGCCTCTCCTTGCTCCCGGCCATGGCGCTGCTGCTCCTGGCAATGGCGGTTGCCTCCTCCGACGCGCAGCCTTCTCCCGGCTACTACCCGAGCTCGAGGTTCAGGCCTGTGGCGTTCAACCGCGGGTACAGTAACAAGTGGGGCCCGCAGCACCAGACGGTCTCTGGCGACCATTCGGCCATCACCATCTGGCTCGACAGGACCTGCGGGAGTGGGTTCAAGTCGAAGCATGCGTACAGGAACGGCTACTTCGCCACCCGCATCAAGCTCCCCGCCGGCTACACCGCCGGCACCAACACCGCTTTCTACCTGTCCAACAACGAGGCGCACCCTGGGTTCCACGACGAGGTGGACATGGAGTTCCTGGGCACCATCCCCGGCGAGCCCTACACGCTGCAGACGAACGTGTACGTCCGGGGCAGCGGCGACGGGCGGATCATCGGGCGGGAGATGCGGTTCCACCTGTGGTTCGACCCCACCGCCGGCTTCCACAACTACGCCATCCTGTGGAACCCGGACGCCATCACCTTCTTCGTGGACGACGTGCCCATCCGGCGGTACGAGCGCAAGACGGAGCTCACCTTCCCGGACCGCCCGATGTGGGCGTACGGCTCCATCTGGGACGCCTCCGACTGGGCCACCGACCACGGCAGGCACCGGGCCGACTACCGCTACCAGCCCTTCGTGGCCCGCTTCGACCGCTTCGTGGTCGCCGGGTGCGGGCCCGGCGCCCCGCCCTCGTGCCGCCCGGTCCGGGCGTCCCCCGTCGGCACGGGGCTCACGCGGCAGCAGTACGCGGCGATGCGGTGGGCGCAGCAGCGCCACATGGTCTACTACTACTGCCAGGACTTCCGGCGGGACCGCTCGCTCACGCCCGAGTGCTGA

>HvXTH19

ATGGCTCGCATGGCGGTCTCGGTGCTTTCGATCCTCCTCGCCACTTGCGCCCTGGCGGCGGCGAGCTTCGACAAGGAGTTCGACGTTACCTGGGGTGACGGGCGCGGCAAGATCCTCAACAATGGCCAGCTGCTGACGCTGGGCCTGGACAAGGTCTCCGGCTCCGGGTTCCAGTCCAAGCACGAGTACCTCTTCGGCAAGATCGACATGCAACTCAAGCTCGTCCCCGGCAACTCCGCCGGCACCGTAACCGCCTACTACCTGTCGTCGCAGGGTCCTACGCACGACGAGATCGACTTCGAGTTCCTGGGCAACGTCACCGGCGAGCCCTACACGCTGCACACCAACGTGTTCACGCAGGGGCAGGGCCAGCGGGAGCAGCAGTTCCGCCTCTGGTTCGATCCCACCAACGACTTCCACACCTACTCCATCCTTTGGAACCCAAAGCACATCATCTTCATGGTGGATGACATGCCGATCAGGGACTTCAAGAACCTGGAGGGAAAGGGGATCGCCTTCCCCAAGAACCAGCCCATGCGGCTCTACTCCAGCCTCTGGAACGCCGACGACTGGGCCACGCAGGGCGGCCGGGTGAAGACGGACTGGTCCCACGCGCCGTTCTCCGCCTCCTACCGCGGCTTCAAGGCCGACGCGTGCGTGGTGACCGCGGGCGGGCGGCCTCGCTGCGGCGCCAGCGTCGGCACGGACGTCGCCCCCGGCACCGGCGCGGCCGGCGAATGGTACAATCAGGAGCTGGACCTGACACGGCAGCAGCGGATGCGGTGGGTGCAGAGCAACTACATGATCTATAACTACTGCACCGACCCCAAGCGTTTCGCTCAGGGCGTCCCCGCCGAGTGCTCCATGTAG

>HvXTH15

ATGGCTTCCAGCGTGCGGCAGCCATGGCTCCTCCTCCTGCTCGTGCTCCTCCCGGTCATGGCCACGGCGGCGGTGTTCGACGACAACTACGCGCCGACGTGGGGCGCAGACGGCTACCACCTCGTCGACCAGGGGACGGAGATCCGTCTCACCATGGACAGAAACTCCGGCGCCGGGTTCCACTCCAAGTCGACGTACGGGTCGGGGTTCTTCCACATGAGGATCAAGGTGCCCGGGGGGTACACGGCCGGAGTCGTCACGGCCTTCTATCTGGCGTCGGAAACACCTTACGATGGCAGTGACCGCGACGAGGTGGACTTCGAGTTCCTGGGCAACGTGGACGGCGAGAACATCACCCTCCAGACCAACGTCTTCGTCAACGGCGACGGCGATAGGGAGCAGAGGCTGAGCCTGTGGTTCGACCCGGCAGCCGACTTCCACGAGTACAAGATACTCTGGAACCCTTACCATCTCGTCATACTGGTGGACGATGTGCCGATACGGGTGCTGAGGAACCTGACGGGGCAGGTGGCGGAGTACGAGTTCCCGGCGAAGCGGATGGCCGTGCGGGCGAGCCTGTGGGACGGCTCCGACTGGGCGACGGACGGCGGCAGGACCAAGATCGACTGGGGCCGCGCGCCCTTCACGGCGGGGTTCCGGGGCTTCGACGTCGACGCCTGCGACAACGCCAGCTCGACGCCGTGCGACTCGACGGACCTGTGGTGGAACGCCCGCAGGCACAGGCGGCTGTCCGTCCGGGAGCAGGCGGCCTACGAGAACGTGCGGAGGACGTACATGAACTACGACTACTGCGCCGACAAGGATCGGTTCCAGAACGGCAAGCTGCCGGTCGAGTGCAGCTACACTACTTAG

>HvXTH11

ATGGCGTCCAGCTCATCGTGCCCTCCTCCGTCGCCGCGCCCCTCCCGCCTCCTCCCCGTGCTCGTCGCCACGGTCGTCCTGCTTGGCCGCGGCGGCGAGGCCAGGCAGCCGGCGCCGCTCCACGGCGTCGTGCGGTCCATGGCCTTCGACGAGGGCTACACCCAGCTCTTCGGCAGCGGCAACCTCGCCCTCCGCCGCGAGGGCAAGCGCGTCCACCTCGCCCTCGACGAGTCCACCGGCTCCGGGTTCGCCTCCCAGGACCGGTTCCTCCACGGCTTCTTCAGCGCCGCAGTGAAGCTCCCTGCCGACTACGCCGCCGGCGTCGTCGTCGCGTTCTACCTGTCGAACGCCGACGTGTACGAGAAGACCCACGACGAGCTGGACTTCGAGTTCCTGGGCAACGTGCGCGGGCGCGAGTGGCGGGTGCAGACCAACGTGTACGGCAACGGCAGCACCGGCGCCGGCCGGGAGGAGCGCTACGACCTCCCCTTCGACCCCACGGACGACTTCCACCACTACTCCATCCTCTGGACCCAACACCGCATCATATTCTACGTTGATGAGACCCCGATCAGGGAGGTGGTGAGGACGGAGGCCATGGGCGCGGCGTTCCCCTCCAAGCCCATGTCCCTCTACGCCACCATCTGGGACGGCTCCGCCTGGGCCACCCTCGGCGGCCGCTACAGGGCCAACTACAAGTACGCGCCGTTCGTCGCCGAGTTCGGCGACCTCGTCCTCCACGCCTGCCCCGTCAACCGCATCTACCACTCCGCGGCGGCGGCGTGCGGCACGCCCTGGTACGAGCCTGTCGCCGCCGCCTTGTCCGGCGAGCAGCGCGCGTCGATGTCGGCGTTCAGGCGCGGGCACATGTCCTACTCCTACTGCCACGACCGCCGCCGGTACCCGGTCGCCCTGTCAGAGTGCGACGTCGCCGTGCTCCCGCGCCTGTTCGGCCCGGACGGGATGAAGTACGGCGGCGACCGCCGGCACCGCCGCGGAGGGCGCGGCCGCCGCTCCGACGTCGTCATGTGA

>HvXTH3

ATGAAAGCACCCTCTGGTCTCGGTCTAGCCTATAAGAAAGCTGTGTCCTGTGCCTTGTGCTTTGCCCCGGACCAAAGCATTAGCACGCTCCTCCACAGTCCTCCCCTCTGCCTGCCCCTGTGTGGCTTTGCCGTTTCCCGTCCCACATTCGTCGTCGGCGGTACAGTATTCGTTTCCTCTTGGGGGTGGGGAGCGATGGGACCTTGGAGGCGTCCGTGCGTCGGCGCTCTCCTGGCGTGCGCCGCCATTGCGGCTTCTTGCTGCTGCTTCCAGCTCCAGGGCGCTGATGCGGCGGCGAGCCCGTCGTTCGGGGACAACTTCGAGATCACCGGCGCCAAGGACCACGTCAAGACCTCCCCCGACGGCCAGACGTGGTACCTCTCCCTCGACAACAAGACGGGCGTCGGGTTCCAGACGAAGCAGAAGTACCTGTTCGGGTGGTTCAGCATGAAGCTCAAGCTCGTCGGAAACGACTCCGCCGGCGTCGTCACCGCCTACTACATGTGCTCGGACCTTGACGCTGCGCCGGAGCGCGACGAGCTGGACTTCGAGTTCCTGGGCAACCGCACCGGCGAGCCGTACATCATCCAGACGAACGTGTACCGCAGCGGCGTGGGCGGGCGGGAGATGCGGCACTCGCTGTGGTTCGACCCCACCGCCGACTTCCACAGCTACTCCATCCTCTGGAACCCCAAGCAGATCGTGTTTTTCGTGGACAAGGTGGCGATCAGGGAGTACCGGAACTCTGCCAAGCCCAACAAGTTCTTCCCGATCATGAAGCCCATGTACGTCTTCTCCAGCATCTGGAACGCCGACGACTGGGCGACGCGCGGGGGCCTGGAGAAGACGGACTGGACCAAGGGGCCCTTCGTCTCCTCCTACAGCGACTTCACCGCCGACGCCTGCGCCTGGCCGTCCGGCCCGGCCCCGCCGGCCTGCGCGGCCGCCACCGGGGACAGCTGGTGGGACCAGCCGCCGGCGTGGGCGCTCGACGACGGCCAGCGCCGGGACTCGGGCTGGGTGGCCAGGAACCTCGTCATATACGACTACTGCGGCGACCGCAAGAGGTTCCCGACCGTGCCGGAGGAGTGCGCGCTCAGGACCACGACTAGCTAG

>HvXTH2

ATGGGAAAGCCGGGGGCACTGGTTCCAGTGGTAGCTCTAGCTTTTGCGTTGGTTCTTGGCCTCGAGCTCGTGTCCGGCGGCAACTTTTACGAGGAGTGCGACGCTACGTGGGAGCCCCAGAACTGCTGGACCTACGACGGCGGCAACAGCCTCTCCCTCGCCCTCGTCAGCAACTCCTCAGGTCTTTCTTCAACCTCACAACTTCTGTATTTTTCATGGAGTTCTCCTCTGAATGAATCAATGTGTGGGTGCTCAGGCTCGATGATCCGGTCCAAGAGGCAGTTCATATACGGAACGGTGTCGACCATGATCCAACTCGTCAAGGGCGACTCCGCCGGCACCGTCACTACATATTACACATCGTCGGTGGGGGACGACCACGACGAGATCGACTTCGAGTTCCTGGGGAACGAGACGGGGCAGCCCTACACGCTGCACACCAACGTCTACGCCGCCGGCGTCGGCGGCAAGGAGATGCAGTTCCGCCCCTGGTTCGACCCCACCGACGGCTACCACAACTACACCATCGCCTGGACGCCCTGCGCGGTCGTCTGGTACGTGGACGGGGCGCCCATCAGGGCGTTCCGCAACTACGAGCGCACCCACGGCGTGGCCTTCCCGACGACCCGCCCCATGCACGCCTATTCCAGCATCTGGGCGGCCGAGGACTGGGCCACGCAGGGCGGCCGCGTCAGGGCAGACTGGACCCGCGCGCCATTCGTCGCCAGCTACCGCGGCATCGACCTCGACATTTGTGAGTGCTACGGCGGCGACTGCGTCTACACCTGCGCCGGGGCGTTCCGGGGCTGCGGCGGGCTCACCGGAGACCAGCGGGGGAAGATGCAGTGGGTGCAGGACAATTACAGGATCTACGACTACTGCGCCGATCACGAGGCCGGCAAGGTGCCCGGCGTCGAGTGCAGCCTGCCGCAGTACTGA

>HvXTH14

ATGGCGCCAAGGTCAGACCTCCTCGCCGCGCTAGCGTTGGCCCTCCTCGCCGCGAGCGTCCTTAGTACGGGGGCCAAGGCCGACTTCGACGACCAGTTCGAGGTGATCGGCGACCGCGACCACATCGGGTACCGGGACGACGGCAACGACAAGGGCCAGGAGTTCTCGCTGGAGCTCGACCAGGAGTCCGGCTCCGGCTTCAAGTCCAAGGCCAAGTACCTCTTCGGCGAGTTCCAGGTCCGGATGAAGCTCGTCGACGGCAACTCCGCCGGCACCGTCACCTCCTTCTACCTGACCTCCGGCGAGAGCGCCACCCACGACGAGATCGACATCGAGTTCATGGGAAACTCGAGCGGCGACCCCTACGTGATGAACACCAACGTCTGGGCCAGCGGCGACGGCAAGAAGGAGCACCAGTTCTACCTCTGGTTCGACCCCTCCGCCGACTTCCACACCTACAAGATCACATGGAACCCAAAGAACATCATATTCGAGGTGGACGGCGTGCCGGTGAGGACCTTCAAGAAGTACGACGGCCTGCCGTTCCCGTCGGCGCGGCCGATGACGGTGCACGCGACGCTGTGGGACGGCAGCTACTGGGCGACGCAGCACGGCACCGTCAAGATCCACTGGCGCCACGACCCCTTCGTCGTCCCCTACCAGGGCTACCACGCCAACGGCTGCGTCCACGACAAGGCCACCAACAAGACCTCCTGCCCCGCCGGCAGCGACGCCTGGATGCACCGCGAGCTCGACGACGGCGAGCTCAGCACCGTCGCGTGGGCCGAGCGCAACTGCCTCTCCTACAACTACTGCGCCGACGGATGGCGCTTCCCCAAGGGCTTCCCCGGCGAGTGCGGACGCAAGTGA

>HvXTH12

ATGGAGATGACGGCGAGGTTCTTGGCCGCGGCGGCGGCGTGCGTGTGGCTGGCGGCGGCGGCCTCCGCCTTCGACGTGCCGACCGTGGCCTTCGAGGAAGGGTTCTCGCCGCTGTTCGGGGACGGCAACCTCGTGCGCGCGCGGGATGATAGGGCCGCCCGCCTCTTGCTCGATCGCCGCTCCGGTTCGGGGTTCATCTCCTCGGATTACTACCTGCACGGCTTCTTCAGCGCGTCCATCAAGCTGCCCCGGGACTACACGGCCGGCGTCGTCGTCGCCTTCTACCTGTCGAACGGGGACGTGTACGAGAAGACGCACGACGAGCTGGACTTCGAGTTCCTGGGCAGCCGGTGGGGCGGGCAGTGGCGGGTGCAGACCAACGTCTACGGCAACGGCAGCACCAGCCGCGGCCGGGAGGAGCGCTACCTCCTCCCCTTCGACCCCACCCTCGCCGCCCACCGCTACTCCATCCTCTGGGCCCCCACCCACATCATATTCTACGTGGACGACACGGCGATCCGGGAGGTGGTGCGGCACCCCGGCATGGGCGGCGACTTCCCGGCGAAGCCCATGGCGGCGTACGCCACCATCTGGGACGGCTCCGCCTGGGCCACGGAGGGCGGCAAGTACAAGGTGAACTACAAGTACGCGCCCTTCGCCTCCGACTTCTCCGACCTGTCCCTCCGCGGCTGCCGCGTCGCCGACCCGGCGTCGCCGGCGCTGCGCCTCGCCGGCGGCGACGGGTGCGACCTCCTGGGGCTCATGACGGCCGACTACGCGGTCATGACCCCGCAGAAGCGCGCCGCCATGCGCGCGTTCCGGGCGCGCCGGATGACCTACACGGTGTGCTACGACGCGGCGCGGTACGCGGCCGGCCCCTTCCCGGAGTGCGACAACTCGGACGAGGAGAGGGGCACGTTCTGGGCGTGGGGCGAGTCCAAGACCGTCGTCATGAAGACGCGCGGCCGCGGCCGCCGCGGCCGGGGCAGCAGGGCCGGCGCCGGAGCGAGGGGCCGCGCCGGCGCGGCGAGCAGCTGA

>HvXTH10

ATGGCGATGATGCAGATTAGGCGGCCGCATGATGCCATCTCACATCTCATGGTGATCGTAGTAGGAGCTGTGATACTGCTGCAAGGTGAGGCGCAGCCATCCCCTGGGTACTACCCGAGCTCCAAGGTGAGCTCAACGCCATTCTCGCAGTGGTACAGCACCCTGTGGGGGCCGCAGCACCAGTCTCTGTCGCCGGACCAGACCGCCCTCACCCTCTGGATGGACCGCAGCTCAGGCAGCGGGTTCAAGTCGAAGCGGTCGTACCGGAACGGCTACTTCGGCGTCTCCATGAAGGTCCAGCCCGGCTACACCGCCGGCGTCAACACCGCCTTCTACCTGTCGAACAACGAGGTGTACCCGGGGTACCACGACGAGATCGACGTGGAGCTGCTGGGCACGGTGCCCGGCGAGCCCTACACGCTGCAGACCAACGTGTACGTCCGGGGCACGGGGGACGCCCACCCCATCGTCGGCCGGGAGATGCGGTTCCACCTCTGGTTCGACCCGGCCGCGGCGTTCCACCACTACGCCGTGCTCTGGAACCCCGACGAGATCGTCTTCCTCGTCGACGACGTGCCGGTGCGCCGATACCAAAAGAAGGTGGAGGCCACGTTCCCGGAGCGGGAGATGTGGGCGTACGGCTCCGTCTGGGACGCCTCCGACTGGGCCACCGACGGCGGCCGCTACAGGTCCGACTACCGCTACCAGCCCTTCGTGTCCGGGTTCAAGGACTTCAAGGTCGCCGGCTGCGAGGTCGGCGCGCCGGCGTCGTGCCGCCCCGTGCCGGCGGGGCCCGGAGGCGGGCTGAGCGCGCAGCAGAGCGCCGCCATGAGCTGGGCGCAGCAGAGGGCCATGGTCTACTACTACTGCCAGGATGGATCCAAGGACCGCTCCAACTACCCAGAGTGCTAG

>HvXTH13

ATGGCGCCGTCGTTGCCGTCATCCTCTTCTTGTTGGCATTCCGCGCTGCTGGTAGCCATGTTGGTGCTTGTGGTGGTCATGGATCAGGTGGCCATGGCGTACCTGGACGACGACATCGAGGTGGTGTGGGGCGACGACCACAGCTTCTTCTACATGGACGACGCCGGCGACGACGAGATCCTCGCGCTCTGCCTCGACGAGACCCACGGCTCGGGGTTCCACACCAAGGAGGCCTACCTCTACGCCCGCTTCGACGTCGACCTCATGCTCGTCCCCGACAACTCCGCCGGCACGGTCACCACGCTCTACCTGATGCCGGAGGACGTGCCGTGGGACTACCACGACGAGGTGGACCTGGAGTTCCTGGGCAACGTCACCGGCGAGCCCTACACGCTCCACACCAACATCTTCGCCAACGGCGTGGGCAACCGCGAGGAGCAGTTCCGCCTCTGGTTCGACCCCACCGCCGACTTCCACACCTACTCCATCGACTGGAACCCCAAGCGCATCACGATCCTGGTGGACGGCGTGCCGATCCGGAGCTTCAGGAACAATGAGGAGCACGGGGTGGCGTTCCCGACGTGGCAGAAGATGCGGCTGCACGGGAGCCTCTGGAACGCCGACGACTGGGCGACGCAGGGCGGCCGCGTCAAGACGGACTGGTCGGGGGCACCATTCTTCGCCCGCTATCGCAACCTCCGGGCGTCGTGGTGCCGGCCGTCGCCGGGGGTGGCGTGGTGCGGCGACGAGCCGCCGGGGTCGACGTGGTTCGAGCGCGGCCTGGACGCGGCGGCGCTGAGGCGGGCGCGCGACGCCCACATGATCTACGACTACTGCAAGGACCTCCAGCGGTACAAGGGGTCGGGGCTCCCCAAGGAATGCGTCGTGGACTGA

>HvXTH17

ATGGCTCGCATGGGGGCGTCGGTGCTGGTGATCCTGCTCGCCTCTTGTGCCCTGGCGGCGGCGAGCTTCGACAAGGAGTTCGACGTTACCTGGGGTGACGGGCGCGGCAAGATCCTCAACAATGGCCAGCTGCTGATGCTGGGGCTGGACAAGGTCTCCGGCTCCGGGTTCCAGTCCAAGCGCGAGTACCTCTTCGGCAAGATCGACATGCAGCTCAAGCTCGTCCCCGGCAACTCCGCCGGCACCGTCACCGCATATTACCTGTCGTCGCAGGGTCCGACGCACGACGAGATCGACTTCGAGTTCCTGGGCAACGTCACCGGCGAGCCATACACGCTGCACACCAACGTGTTCACGCAGGGGCAGGGCCAGCGGGAGCAGCAGTTCCGCCTCTGGTTCGATCCTACCAACGACTTCCACACCTACTCCATCCTCTGGAACCCGAAGCACATCATCTTCTTGGTTGACGACATGCCGATCAGGGACTTCAGGAACATGGAGGGAAAGGGGATCGCCTTCCCCAAGAACCAGCCTATGCGGCTGTACTCCAGCCTCTGGAACGCCGACGACTGGGCGACACAGGGTGGCCGCGTCAAGACCGACTGGTCCCACGCTCCGTTTTCCGCCTCCTACCGCGGCTTCAAGGCCGACGCGTGCGTGGTGACCGTAGGTGGCCGGCCGCGCTGCGGCGCCAGCATCGGCACGGACGCGGCCCCAGGGACCGGCGGCGCGGCCGCGGTCGGCGACTGGTACAACCAAGAGCTGGATCTTACGCGGCAGCAGCGCATGCGTTGGGTGCAGAGCAATTACATGATCTACAACTACTGCACTGACCCCAAGCGCGTCGCCAAGGGCGTCCCTGCCGAGTGCTCCATGTAG

>HvXTH21

ATGGCGTCCGGTCCCAGTAGAACAGTCCCGTGCTCTGTGCTGCCACTGCTGCTGCTGCTCGCCGGCGTGGCCCGCGCGGCCGGCAACTTCTACCAGGACGTGGACATCACGTGGGGCGACGGGCGCGGCAAGATCCTCGGCGGCGGCGACCTCCTCACGCTGTCGCTCGACAGGGCCTCCGGCTCCGGGTTCCAGTCCAAGAACCAGTACCTGTACGGCCGCTTCGACATGCAGATCAAGCTCGTCCCCGGCGACTCCGCCGGCACCGTCGCCACTTTCTACCTGTCGTCGCAGGGGTCGGCGCACGACGAGATCGACTTCGAGTTCCTGGGCAACGCGAGCGGGCAGCCCTACACGGTGCACACCAACGTGTACAGCCAGGGCAAGGGCGGCCGGGAGCAGCAGTTCCGCATGTGGTTCGACCCCACCGCCGACTTCCACACCTACTCCGTCCTCTGGAACCCCACACACATCCTGTTCTACGTGGACGGGACGCCGATACGGGAGCACCGCAACCGGGAGGCGGCGACGGGGGTTCCCTACCTGCGGAGCCAGGCGATGAGGGTGTACGCGAGCGTGTGGGACGCGGAAGAGTGGGCGACGCAGGGCGGGCGGGTGAGGACGGACTGGTCGCGGGCGCCGTTCGTGGCGTCGTACAAGGGGCTCGCCGCGAGCGGGTGCGCGTCGCAGGACGCGGCGGCGTGCGCCAACTCCAACGGCGCGTGGATGTACCAGGAGCTGGACGCCACGGCGTTGGACCGCCTCCAGTGGGTGCAGAAGAACTACATGATCTACAACTACTGCACGGACACGTGGAGGTTCAAGGACGGCGCCCCGCCCGAGTGCGCCAGCAAGTAG

>HvXTH24

ATGGGCCAGGCTAGGGCTTACCTCCTAGCCTCCCTAGCGGCGTTCTACCTCGTCGCCCTGGCCATCCCCCAGGTCACCGCCGACATGACCGACGAAGTCAATCTCCTGTGGGGCAACTGCAAGGTTCAACGCGATGGCACCGGCCGACAGACTGTCGCGATGAGTCTCGACCGCTGGACGACTTCAGGATTCTCCTCGAAAATCAAGTACCTATTCGGGAGGATTGACATGGAAATCAAGCTCATGCCCGGGAACTCAGCCGGCACAGTGACAACATTTTATATGATGTCAGAGGGACCATGGCAATTCCATGATGAAATCGACCTTGAATTCTTGGGGAACAGCACCGGCAACCCCTACACCCTGCACACCAACGTGTATGCCAGAGGTGTAGGCAGCAGAGAGAAGGGGTACCGGCTTTGGTTTGATCCCTCCCAGGACTTCCACACCTACAGCATCATTTGGACCCAACAATACATCAGATTCCTGGTCGATAACAAGCTGATCAGGCAGATCAAGAACAAGATGATGAATGGTTCCCCCTATCCAAACTATCAACCAATGAGGGTGTTCAGCACCATCTGGAATGCGGATGACTGGGCGACACAGGGTGGGCGGGTCAAGACCGACTGGACACAAGCGCCATTCACAGCATACTTCCGGAACTACAAGGCCACCAGCTGCTCTCAAGGCCAGAACTCCAACGTCTGCGGCCAGAGCTCCCCCAACGGTTTGTTCAACCAGCAGCAGGACCAGATGCAGCAACAGCAAGTGAAGGAGGTGGATGCTAAATACAAGGTCTATGATTTCTGCGATGACTCAAAGAGGAGGATTGGGTCCTCCGAGGACTGTCAATCACAGTAG

>HvXTH23

ATGAGGACGGTGGAGCTCGGCATTGTGGCCATGGCGTGCCTCGTCGCGGTGGCGCGGGCCGGCAACTTCTTCCAGGACTCGGAGATGTCCTGGGGGGACGGCCGCGGGAAGGTCGTCGACGGCGGCCGCGGGCTCGACCTCACGCTCGACAAGACCTCCGGCTCCGGCTTCCAGTCCAAGAGCGAGTACCTCTTCGGCAAGATCGACATGCAGATCAAGCTCGTCCCCGGCAACTCCGCCGGCACCGTCACCACCTTCTACCTGTCGTCGCAGGGGACGGCGCACGACGAGATCGACTTCGAGTTCCTGGGTAACGTCACCGGCGAGCCCTACACGCTGCACACCAACGTGTTCGCGCAGGGGCAGGGGCAGCGGGAGCAGCAGTTCCGCCTCTGGTTCGACCCCACCAAGGCCTTCCACACCTACTCCATCATCTGGAACCCGCAGCACGTCATATTCGCGGTGGACGGCACGGCGATCAGGGACTTCAAGAACCACGAGGCGCGGGGCGTGTCGTTCCCCAAGAGCCAGCCGATGCGGCTGTACGCGAGCCTGTGGAACGCCGACGACTGGGCCACGCAGGGCGGCCGGGTCAAGACCGACTGGAGCAAGGCGCCGTTCGTCGCCTCCTTCCGCAACTTCAACGCCGACGCCTGCGTCATGTCGGGCGGCGCGCAGCGCTGCCCCGCCGGCACCATGGAGGCCTCGGCGGCCGGCGGCGGCAGCTGGTGGAACCAGGAGCTCAGCGGCATGGGGTACCGCCGCATGCGGTGGGTGCAGAGGAAGTTCATGATCTACAACTACTGCACCGACCCCAAGCGGGTGGCGCAGGGCGTGCCCGCCGAGTGCAAGCTCCGCTGA

>HvXTH18

ATGGCTCGCATGGGGGCGTCGGTGCTTTCGATCCTGCTCGCCTCTTGCGCCCTGGCGGCGGCGAGCTTCGACAAGGAGTTCGACGTTACCTGGGGTGACGGGCGCGGCAAGATCCTCAACAACGGCCAGCTCCTGACGCTGGGACTGGACAAGGTCTCCGGCTCCGGGTTCCAGTCCAAGCACGAGTACCTCTTCGGCAAGATCGACATGCAGCTCAAGCTCGTCCCCGGCAACTCTGCCGGCACCGTAACCGCCTACTACCTGTCGTCGCAGGGGCCGACGCACGACGAGATCGACTTCGAGTTCCTGGGCAACGTCACCGGCGAGCCCTACACGCTGCACACCAACGTGTTCACGCAGGGGCAGGGCCAACGGGAGCAACAGTTCCGCCTCTGGTTCGATCCCACCAACGACTTCCACACCTACTCCATCCTCTGGAACCCAAAGCACATCATCTTCATGGTGGACGACATGCCGATCAGGGACTTCAAGAACCTGGAGGGGAAGGGGATCGCGTTCCCCAAGAACCAGCCCATGCGGCTCTACTCCAGCCTCTGGAACGCCGACGACTGGGCCACGCAGGGCGGCCGCGTCAAGACCGACTGGTCCCACGCGCCGTTCTCCGCCTCTTACCGTGGCTTCAAGGCCGACGCGTGCGTGGTGACCGCGGGCGGCCGGCCGCGCTGCGGCGCCAGCATGGGCACGGAAGCGGCCCCGGGCACGGGCGCTTCCGGCGCGGCCGGCGAGTGGTACAACCAGGAGCTGGACCTGACGCTGCAGCAGCGGATGCGGTGGGTGCAGAGCAATTACATGATCTACAACTACTGCACTGACCCCAAGCGCGTCGCCAAGGGCGTCCCCGCCGAGTGCTCCATGTAG

>HvXTH1

ATGGCGAGGCCGTCCTTCTCCCTCCACCTGTGCCTGGCCGTTCTGGCCTTGGCCGCCGCCGCGTCGGAGGCCGGGTTCTACGACCAGTTCGACGTGGTCGGCTCCGGCAACAACGTCCGCGTGAACGACGACGGCATCGCCCAGCAGGTGGCGCTCACGCTCGACCAGGGCAACGGCGGCTCCGGCTTCAGCTCCAAGGACAAGTACCTCTACGGCGAGTTCAGCGTCCAGATGAAGCTCATCGGCGGCAACTCCGCCGGCACCGTCACCTCCTTCTACCTGACGTCTGGGGAGGGCGACGGCCATGACGAGATCGACATCGAGTTCATGGGCAACCTCAGCGGCGACCCCTACGTGATGAACACCAACGTCTGGGCCAGCGGCGACGGCAAGAAGGAGCACCAGTTCTACCTCTGGTTCGACCCCACCGCCGACTTCCACACCTACAAGATCGTCTGGAACCCCAAGAACATCATATTCCAGGTGGACGACGTGCCGGTGAGGACGTTCAAGAAGTACGACGACCTGCCGTACCCGAGCAGCCAGCCGATGACGGTGCACGCCACGCTCTGGGACGGCAGCTACTGGGCCACCCGCCACGGCGACGTCAAGATCGACTGGACCCAGGCGCCCTTCGTCGTCAACTACCGCGGCTACACCTCCAACGGCTGCGTCAGCAACGGCGGCTCCTCCGCGTGCCCCGCCGGCAGCGACGCCTGGATGAGCACGGAGCTCGACGCCAAAGCCCTCGGCACCGTCGCCTGGGCCGAGAGCAAGTACATGTCCTACGACTACTGCACCGACGGCTGGCGCTTCCCCAACGGCTTCCCCGCCGAGTGCTCCCGCCGCAACTGA

>HvXTH6

ATGGCACAGCGCTTTCTGGCTGTGCTCGCCGTCGCCCTGGCGCTCTCGCAGGTCGCCTCAGCTAAGTCCTGGCTCGATAAAAGGTTCAACACCGACGGCACCGTCCGCACCGGATACGACGCCTCGGGCCAGCAGGTGGTTATGCTCAACCTCAACCAGCAATCCGGCGCCGCCGGATTCAACTCCAAGCAGCAGTACCTCTACGGCGAGTTCAGCATCCAGATGAAGCTCATCCCGGGAAACTCCGCCGGCACCGTCTCCTGCTTCTACCTTTCTTCCGGTGACGACGAGTGGCGCGACGAGATCGACATGGAGTTCATGGGCAACTCCAGCGGCCACCCGGTGGTGCTCAACACCAACGTGTGGGCCAACGGCGACGGCAAGAAGGAGCACCAGTTCGACCTCTGGTTCGACCCAGCCGCAGACTACCACACCTACACCATCATCTGGAACCCGGAGAACATCCTCTTCAAGGTGGACAACCTCTTCATCCGATCCTTCAAGCGCTTCGCCGGCCTCCCTTACCCTACCTCCAAGCCCATGAGGCTGCACGCCACGCTCTGGGACGGCAGCTACTGGGCGACCGAGAAGGGCAAGATCCCGATCAACTGGTCCAACGCGCCATTCGTCGTCTCCTACCGCAACTACTACGCCAACGCCTGCGTCAGCGGCGGCGCGTGCCATGCCGGCAGCGACAGGTGGATGAGGAAGCAGCTCGACGGCGACGAATGGGGCACCGTGAAGTGGGCGGAGCGCAGTTACATGCGCTACAACTACTGCGAGGATGGGTACAGGTTCCCGCAGGGGCTTCCCGCCGAGTGCAACCGCTACTGA

>HvXTH5

ATGGCACGGCGTCTTCTCGCTGTGCTCGCCGTGGCTCTTGCGCTCTTGCAGGCCGCCTCGGCCAAGTCCTGGCTCGACAAGAGGTTCAACACGGACGGCACCGTCCGCACGGGATACGACGCCTCGGGCCAGCAGGTGGTGATGCTCAACCTCAACCAGCAATCCGGCGCCGCCGGCTTCAACTCCAAGCAGCAGTACCTCTATGGTGAGTTCAGCATCCAGATGAAGCTCATCCCGGGGAACTCCGCTGGCACCGTCTCCTGCTTCTACCTTTCTTCCGGTGACGACGAGTGGCGCGACGAGATCGACATGGAGTTCATGGGCAACTCCAGCGGCCATCCGGTGGTGCTCAACACGAACGTGTGGGCCAACGGCGACGGCAAGAAGGAGCACCAGTTCGACCTCTGGTTCGACCCCGCCGCCGACTACCACACCTACACCATCATCTGGAACCCGGAGAACATCCTGTTCAAGGTGGACAACCTCTTCATCCGATCCTTCAAGCGCTTCGCCGGCCTGCCCTACCCTACCTCCAAGCCCATGAGGCTGCACGCCACGCTCTGGGACGGCAGCTACTGGGCGACCGAGAAGGGCAAGATCCCCATCAACTGGTCCAACGCGCCATTCGTTGTCTCGTACCGCAACTACTACGCCAACGCCTGCGTCAGCGGCGGCGCGTGCCATGCCGGCAGTGACAGGTGGATGAAGAAGCAGCTCGACGGCGCCGAATGGGGCACCGTGAAGTGGGCGGAGCGAAGTTACATGCGGTACAACTACTGCGAGGATGGGTACAGGTTCCCACAGGGGCTTCCCGCCGAGTGCAACCGCTACTGA

>HvXTH7

ATGAGCAATACCTCTACGCTTTCTTCCGGTGATGGAGACGGGCACGACGAGATCGACATGGAGTTCATGGGCAACTCCAGTGGCCCTGGCCATCCGGTAGTGCTCAACACCAACGTCTGGGTCAACGGCGATGGCAAGAAGGAGCACCAGTTCGACCTCTGGTTCGACCCCGCCGCCGACTACCACACCTACACCATCATCTGGAACCCGGAGAACATCCTCTTCAAGGTGGACAACCTCTTCATCCGGTCCTTCAAGCGCTTCGCCGGCATCCCCTACGCTGGCTCCAAGCCCATGAGGCTGCACGCCACGCTGTGGGACGGCAGCTACTGGGCGACCGAGAAGGGCAAGGTCCCCATCGACTGGTCCAACGCACCCTTCAACGTCTTGTACAAAAACTACTACGCCAACGCCTGCGCCAGCGGCGGCGCTTGCCATGCCGGCAGCGACGGGTGGATGAACAGGCAGCTCGACGGCTCCGAGTGGGGCACCGTCAAGTGGGCGGAGCAAAATTACATGCGCTACAACTACTGCGCAGATGGCTACAGGTTCCCACAGGGGTTCCCCGCCGAGTGCAGCCGCTACTGA

>HvXTH4

ATGGCGCCGGCATTGCCTTGTAGCAGGCCAAAGCTGCTGCTCCTGTGCGTGGCCCTGGCCTTCCTCCTGGCCGTGGACGTGGGCAGGGCGGACATCTACAAGGACATCCAGATCATATGGAGCGCGGACCACACCTACTACTTCATGGACGGCGACAGCGAGGCGCTGGCGCTCTCGCTCGACTTCAACCGCGGCTCCGCCTTCAAGTCCAACGACATGTACCTCTACGCCCGCATCGACATCGACATCAAGCTCGTCGAGGGCAACTCCGCCGGCACCGTCTGCACCGTCTACACCATCTCGGAGGGGCCGTGGGACATCCACGACGAGATCGACCTGGAGTTCCTGGGCAACTCCACCGGCGAGCCCTACACCCTCCACACCAACATATTCGCCTACGGCGTCGGCGGCCGGGAGCAGCAGTTCAAGCTCTGGTTCGACCCAAGCGCCGAGTACCACACCTACTCCATCGTCTGGAACCCCAGGCGCATCACGATCGAGGTGGACGGCGTGACGATCCGTTCCTACGACAACAACGAGGAGCACGGCGTGCCGTTCCCGGCGTGGCAGCAGCAGCGGGTGTACGGGAGCCTGTGGAACGCCGACGACTGGGCGACGCAGGGCGGGCGCGTCAAGACGGACTGGAAGCTGGCGCCCTTCGTCTCCTACTACCGCAACTACAACATCACCTACTGCCGGCCGTCGCCGGGTGTGTCGTGGTGCGGCGCCGAGCCCGCCGGCTCCCCGGTCTTCAACCTCGCCCCCAAGGCGCGCGCCGACATGCAGTGGGTGCGCGACATGGGCTACGTCATCTACGACTACTGCACCGACAGGAGCAACCGGTATAACGACACCACCCGGCCCAAGGAGTGCTCGCTCCCGCCACGGCCATGA

>HvXTH9

ATGGCGTGTCACTTCCTCTTGGCCGTCCTCCTGGCGTCGTCTTCTTGGGTTGCTGCGTCCTCCGGCGCCGCCGCGGACGATGTCATGGTGCCCCGCCCGACGACGGCGGCGGCGCTCACCTTCCGGGAGGGCTACACCCAGCTGTTCGGGGACTCCAACCTGAGGCTCCACGGCGACGGCAAGCGAGTCCACATCTCCCTCGACGAGAGGACAGGCTCCGGGTTCGCGTCGCAGGGCGCGTACTTCCACGGCTTCTTCAGCGCCAGCATCAAGCTGCCCTCCGACTACGCCGCCGGCGTCGTCGTCGCCTTCTACGTGTCCAACGGCGACGTGTACGAGAAGACGCACGACGAGCTGGACTTCGAGTTCCTGGGGAACGTCAGGGGGAAGGAGTGGAGGGTGCAGACCAACGTGTACGGCGACGGCAGCACGGCGGTCGGCCGGGAGGAGAGGTACGGCCTCTGGTTCGACCCCACCCACGACTTCCACCGCTACGCCATCCTCTGGACCAACCGCACCATCGTGTTCTACGTGGACGGTACGCCGATCAGGGAGGTGGTGAGGAGCGAGGCGATGGGGGCGCAGTTCCCGTCCAAGCCCATGTCGCTCTACGCCACCATCTGGGACGGCTCCAGCTGGGCCACCTCGGGGGGCCGCTACAAGGTGGAGTACAAGTACGCGCCCTACGTCGCCGAGTTCACCGACCTCGAGCTCCGCGGCTGCGCCTCCCATGATCGAGCCCAGCCGGCGTCGTGCGAGCCGGAGGGAATGCCGGCCAGGCAGCGGGCGGCGATGGAGAGGGTCCGGGCGCGGCACATGACGTACGGGTACTGCTACGACCGCGCGCGGTACCCTGCGCCGCTGCCCGAGTGCAGGGTGGGCGCCGAGGCGGCCATGTACCTCCCCTCGGGCGAGGCCAGGTCGTCGGACCGGCGCAGGCACGGCAAGCGCCACCGTCGTGCCGACTCCGCTCTCTGA

>HvXTH8

ATGAAGGCTACCGCGGGGGCCCTCCTCGCCGTGGTGGCCACGGTGCTACTGCGAGGCATCGCGGCAGCGCCGCCCCGGAAGCCGGTGGACGTGCCATTCGAGAAGAACTACGTCCCGACATGGGCGGAGGACCACATCCACTACGTGAACGGCGGACGGGAGGTGCAGCTGTCCCTCGACAAGACCACCGGCACTGGCTTCCAGACCCGGGGCTCCTACCTCTTCGGCCACTTCAGCATGCACATCAAGCTCGTCGGCGGCGACTCCGCCGGCACAGTCACCGCCTTCTACGTACCGTCGCAGAACTCGGAGCACGACGAGATCGACTTCGAGTTCTTGGGGAACAGGACGGGGCAGCCGTACATCCTGCAGACGAACGTGTTCTCCGGCGGGAAGGGCGACCGGGAACAGAGGATCTACCTCTGGTTCGACCCAACCAAGGACTACCACTCCTACTCCGTCCTCTGGAACCTCTACATGATCGCGTTCTTTGTGGACGACACGCCGATCCGGGTGTTCAAGAACAGCAAGGACCTCGGCGTGCGGTACCCCTTCGACCAGCCTATGAAGCTCTACTCGAGCCTGTGGAACGCGGACGACTGGGCGACTCGGGGAGGGCGGGAGAAGACGGACTGGTCCAAGGCGCCCTTCGTCGCCTCCTACCGGGGCTTCCACGTCGACGGCTGCGAGGCGTCGGCGGAGGCCAAGTTGTGCGCCACCCAGGGCGCCCGCTGGTGGGATCAGCCCGAGTTCCAGGACCTGGACGCCGCGCAGTACCGCCGCCTCGCCTGGGTCAGGAAGGAGCACACCATCTACAACTACTGCACAGACCGCGAACGATACGCCGCCATGTCGCCCGAGTGCAAACGCGACCGCGACGTCTGA
