# Supplementary material for: Genome-Wide Identification, Characterization and Expression Analysis of Xyloglucan Endotransglucosylase/Hydrolase Genes Family in Barley (Hordeum vulgare)
Source: Molecules. 2019 May 20;24(10):1935. doi: 10.3390/molecules24101935 (PMC6572274; doi:10.3390/molecules24101935)
Supplement: Supplementary file 1 [file molecules-24-01935-s001.zip › Supplementary File 3 Genomic sequences of HvXTHs.docx]

**Supplementary File S6：Genomic sequences of *HvXTHs***

>HvXTH20

ATGGCTCGCATGGCGGTGTCGGTGCTGGCGATCCTGCTCGCCTGGTGCGCCCTGGCGGCGGCGAGCTTCGACAAGGAGTTCGACATCACCTGGGGCGACGGGCGTGGCAAGATCCTGAACAATGGTCAGCTCCTGACGCTGGCGCTGGACAAGGTGTCCGGCTCCGGGTTCCAGTCCAAGCACGAGTACCTCTTCGGCAAGATCGACATGCAGCTCAAGCTCGTCCCCGGCAACTCTGCCGGCACCGTCACCGCATACTACGTAGGCCAACATCTGACACGGTTGCTTGCTTGGTTGATGTGCGTGCCTAAGCCTAACTTGATCGGTTTGGTGTGTATGAATGTACGTGCAGCTGTCGTCGCAGGGGCCGACGCACGACGAGATCGACTTCGAGTTCCTGGGCAACGTCACCGGCGAGCCCTACACGCTGCACACCAACGTGTTCACGCAGGGGCAAGGCCAACGGGAGCAGCAGTTTCGCCTCTGGTTCGATCCCACCAACGATTTCCACACCTACTCCATCCTCTGGAACCCAAAGCACATCATGTAAGTTGGTCGATCATGATCACCGGCCATCTAGCTGCTGCAGGTGCAGGCTGCAGCACATGGACTGACCGATTTGGTGTACACAATGCAGCTTCATGGTGGACGACATGCCGATCAGAGACTTCAAGAACCTGGAGGGAAAAGGGATCGCCTTCCCCAAGAACCAGCCCATGCGGCTCTACTCCAGCCTCTGGAACGCCGACGACTGGGCAACGCAGGGCGGCCGCGTCAAGACGGACTGGTCCCACGCCCCGTTCTCGGCCTCCTATCGCGGCTTCAAGGCAGACGCGTGCGTGGTGACCGCGGGCGGGCGGCCTCACTGCGGCGCCAGCGTCGGCACGGACGTCGCGCCCGGCACAGGCGCGGCCGGCGAGTGGTACAACCAGGAGCTGGACCTGACGCGGCAGCAGCGGATGCGGTGGGTACAGAGCAACTACATGATCTACAACTACTGCACCGACCCCAAGAGGTTCGCTCAGGGCGTCCCCGCCGAGTGCTCCATGTAG

>HvXTH16

ATGGCCAGCCTCTCCTTGCTCCCGGCCATGGCGCTGCTGCTCCTGGCAATGGCGGTTGCCTCCTCCGACGCGCAGCCTTCTCCCGGCTACTACCCGAGCTCGAGGTTCAGGCCTGTGGCGTTCAACCGCGGGTACAGTAACAAGTGGGGCCCGCAGCACCAGACGGTCTCTGGCGACCATTCGGCCATCACCATCTGGCTCGACAGGACCTGCGGTACGCGTAATTTTCTTTCTGCACATCTCCGGAAAGACTGCGAGCTGTGGTTTTTAAATTCCAGTTTAGGTGCATGTATATTGGGTCTTGTTTTTTCTTAACAGTGTCACCGACGCACGTACTACGTGAATTCACTGTTCAAGCTTGTGTGCTAGCGAAAGAGACGTCACTAGCGCCATGTTGGATCGTGGGTGTCACTTATGTCGAATCTGAGACACGGACACAGTGCGTTTTTTTCCTTTTGGTAATGGAGGAGCCCTGGGCCTGTGCATCGCACAGCGATAAACACAGTGCGTTTTGGGCCCACGTACGTGGCAGCAGTAACGGACCACTGTTGTCTACTCTTATGCTGATCTAGACCATGGGAGGATTAATTTGCTGCTGCTTGATTATGCTGGTGAACATTCCCTCACCCCTCGCATCTGATTCTGATTTCTGATGTATGCAGGGAGTGGGTTCAAGTCGAAGCATGCGTACAGGAACGGCTACTTCGCCACCCGCATCAAGCTCCCCGCCGGCTACACCGCCGGCACCAACACCGCTTTCTACGTAAGCACCATATATAGTATACATTTCTGAGATTTCTCTAGATAATGCAACGTACGTTAACCGGTAGTCGGTAGAGTAGTAAAGAGTTGACGATTGATACGCGCATGCAGCTGTCCAACAACGAGGCGCACCCTGGGTTCCACGACGAGGTGGACATGGAGTTCCTGGGCACCATCCCCGGCGAGCCCTACACGCTGCAGACGAACGTGTACGTCCGGGGCAGCGGCGACGGGCGGATCATCGGGCGGGAGATGCGGTTCCACCTGTGGTTCGACCCCACCGCCGGCTTCCACAACTACGCCATCCTGTGGAACCCGGACGCCATCACCTTCTTCGTGGACGACGTGCCCATCCGGCGGTACGAGCGCAAGACGGAGCTCACCTTCCCGGACCGCCCGATGTGGGCGTACGGCTCCATCTGGGACGCCTCCGACTGGGCCACCGACCACGGCAGGCACCGGGCCGACTACCGCTACCAGCCCTTCGTGGCCCGCTTCGACCGCTTCGTGGTCGCCGGGTGCGGGCCCGGCGCCCCGCCCTCGTGCCGCCCGGTCCGGGCGTCCCCCGTCGGCACGGGGCTCACGCGGCAGCAGTACGCGGCGATGCGGTGGGCGCAGCAGCGCCACATGGTCTACTACTACTGCCAGGACTTCCGGCGGGACCGCTCGCTCACGCCCGAGTGCTGA

>HvXTH22

ATGCTGCGCGGCTCCCTCCGGTGGCTGCTGGTGCTGGCCGTGGTGGTGGCGGCGTCCGCCGGGAAGGCCGGCCGGGGCCTGCACCGGGACTTCGACGCCGTGTGGGGGAAGCGCAATGCGCGCTTCTTCGACGAGGGCCGGGTGGTGGAGCTGGCGCTGGACCGGGAGACGGGGTCCAGGCTGCAGTCCAAGGACCGGTACCTCTTCGGGCGGTTCGACCTCGACATCAGGCTCGTCGCCGGCGAGTCCGCCGGGACCATCACCTCCTTCTACGTAAGTGCACATGGATCCCTCTGCTGGTCGACTGCCTTTTCTGTTCTCGTTTCCGGCGGCCTCATTAACCGGCATTGGCCATTTCGTTAACTGGTACAGTAAAAAAGAGATTTTCTTTTGCGACTGTTCTTCGGACAACGCCTACGAGTTGGACACTTGTCATTCCTCGAGTGAATTTTGGTTCAGAAAGCAAGGGGTAAAAAAATGCCGAGCAAATTAAGTTGAGAAATGTCTTGCTTGGTCCCCTGCATTTGTCGCCCAACGTAGTCCCCGGAGGAAACAAGTGTGAATTCAGAAGCCGATTCGGAGGTTAACTGACGCCCAACTGCGATGAATTCTGTTCTGGTTGACGCATAAATGTTAGTAACTGATGAATTCGACGGGGTTTCTGCAATGAATGGGGCGGGTGGTAGTACCAGTAGCTACACAGATCTGCCGTGCTACAATTATTGTGTGTATGTACATGTCCTAGATCAAAGGATACGAAGAGCNNTCGTCCTCGCAGCTATTCCAGCAGTTAATACATGGTGTACAATGGCTTGATTAAGTCATGAATTGCATTAAAGAAACGTTCATGGTGAACTAGCCANNNNNNNNNNNNNNNNNNNNNNNNNNNNNNNNNNNNNNNNNNNNNNNNNNNNNNNNNNNNNNNNNNNNNNNNNNNNNNNNNNNNNNNNNNNNNNNNNNNNNNNNNNNNNNNNNNNNNNNNNNNNNNNNNNNNNNNNNNNNNNNNNNNNNNNNNNNNNNNNNNNNNNNNNNNNNNNNNNNNNNNNNCATGGTGAACTAGCCAGGACTGTCTTAGACGTGAATTCCACTAAGGCCGACTAGACACCATCTGAATACTTTATTTTTCAGGAGATAGAGTCTGGATTCTGCACTCTTCAGGACACCAAAACTGAAATTATTCTGCCGTTTTGAGGAGGGAAAAACAATAATTTTCTCGAGCATGGTGAACTAGCCAGGTTTTTTTTAGAAGTGATTTCCACTAAGGCCAACTAAACACCATCTGAATACTGTATTCTTCAAGGATATAGAATCTGAACTCCGTGTTCTTCAGGCGACAGAATCTGGAATTATTCTGCCCCTTTGAGCATATGTAGTCATGGTGTCCACGTTTAAATCTAGTCTGCGTTCCTTTTTTATACTTGTATGACTATGAATTAAGAAAGAGGATGGACGTTGCAGATCTGCACGGGTGGCGCGCGGCACGACGAGGTGGACTTCGAGTTCCTGGGCAACGTGAGCGGCGAGCCCTACATCCTGCACACCAACATCTTCAGCGACGGCAAGGGCGAGCGGGAGCAGCAGTTCGTGCTCTGGTTCGACCCCACCGCCGACTTCCACACCTACTCCATCCTCTGGAACCCGCTCAACATCATGTACTACCCACTACGCCCTCAGATCTCTTCTCCACAGCATCACCATCGTCGTCGTCCTGACACGCATGATCTTGTTGACTGAATGCAGCCTGTACATCGACGGGACGCCGATCAGGGTGTTCAAGAACAACGAGGCCAACGGGGTGCCGTTCCCGACGAGGCAGCCGGTGCACGTCTTCGCCAGCATCTGGAACGCCGAGGAGTGGGCGACGCAGGGCGGCCGCGTCAAGACGGACTGGTCGGAGGCGCCGTTCGTGGCCGCGTACCGGCGCTTCGACGCCAGCAGCGCCTGCGTCTGGCATGGCGGGGCGTCGCCGACGCGGTGCGGCGGCGACCACCTGCCGTCGTCGGCGTCGTCGTGGATGGGGCAGCGGCTGGACTGGTGGAGCTGGATGACGCTCAACTGGGTGCGCATGAACTACATGACCTACGACTACTGCGCCGACCGGAAGCGGTACCCCCACGGGTTCCCCGCCGAGTGCATCATCCCCATCGGGAGGATCTGA

>HvXTH19

ATGGCTCGCATGGCGGTCTCGGTGCTTTCGATCCTCCTCGCCACTTGCGCCCTGGCGGCGGCGAGCTTCGACAAGGAGTTCGACGTTACCTGGGGTGACGGGCGCGGCAAGATCCTCAACAATGGCCAGCTGCTGACGCTGGGCCTGGACAAGGTCTCCGGCTCCGGGTTCCAGTCCAAGCACGAGTACCTCTTCGGCAAGATCGACATGCAACTCAAGCTCGTCCCCGGCAACTCCGCCGGCACCGTAACCGCCTACTACGTAAGCGACCCAACATCCATTCATTCCACTGCTTCGAGGTCTTGATCGATTGACAAGATTGCCTAACCCGATCGGTTTGGCGTGTGTGAATGTGCAGCTGTCGTCGCAGGGTCCTACGCACGACGAGATCGACTTCGAGTTCCTGGGCAACGTCACCGGCGAGCCCTACACGCTGCACACCAACGTGTTCACGCAGGGGCAGGGCCAGCGGGAGCAGCAGTTCCGCCTCTGGTTCGATCCCACCAACGACTTCCACACCTACTCCATCCTTTGGAACCCAAAGCACATCATGTAAGCTATTGATCGCCATTGCCCAACTTGCTTTTTTCTTTCAAGAAGGCTGGCCAACATGGACTGACGGATCGCTTTTGATGTACTTAAATGCAGCTTCATGGTGGATGACATGCCGATCAGGGACTTCAAGAACCTGGAGGGAAAGGGGATCGCCTTCCCCAAGAACCAGCCCATGCGGCTCTACTCCAGCCTCTGGAACGCCGACGACTGGGCCACGCAGGGCGGCCGGGTGAAGACGGACTGGTCCCACGCGCCGTTCTCCGCCTCCTACCGCGGCTTCAAGGCCGACGCGTGCGTGGTGACCGCGGGCGGGCGGCCTCGCTGCGGCGCCAGCGTCGGCACGGACGTCGCCCCCGGCACCGGCGCGGCCGGCGAATGGTACAATCAGGAGCTGGACCTGACACGGCAGCAGCGGATGCGGTGGGTGCAGAGCAACTACATGATCTATAACTACTGCACCGACCCCAAGCGTTTCGCTCAGGGCGTCCCCGCCGAGTGCTCCATGTAG

>HvXTH15

ATGGCTTCCAGCGTGCGGCAGCCATGGCTCCTCCTCCTGCTCGTGCTCCTCCCGGTCATGGCCACGGCGGCGGTGTTCGACGACAACTACGCGCCGACGTGGGGCGCAGACGGCTACCACCTCGTCGACCAGGGGACGGAGATCCGTCTCACCATGGACAGAAACTCCGGCGCCGGGTTCCACTCCAAGTCGACGTACGGGTCGGGGTTCTTCCACATGAGGATCAAGGTGCCCGGGGGGTACACGGCCGGAGTCGTCACGGCCTTCTATGTGAGTCGCTAGCTGATCCGTGCTTGCTTGATTGCTGCTCATGCGTAGTAGCTAGGTCTCGTTGATTAGAATTTGTGCATGCAGAGCTATAACCACTGAGTTTACGTTGCATGTATCATGGATGGATGCATGCAGCTGGCGTCGGAAACACCTTACGATGGCAGTGACCGCGACGAGGTGGACTTCGAGTTCCTGGGCAACGTGGACGGCGAGAACATCACCCTCCAGACCAACGTCTTCGTCAACGGCGACGGCGATAGGGAGCAGAGGCTGAGCCTGTGGTTCGACCCGGCAGCCGACTTCCACGAGTACAAGATACTCTGGAACCCTTACCATCTCGTGTACGTACGTATGCCCCCTAAAACCTAGCCCCATGCATCATGCATGCCTCCATTAATTCCAAGAAAACATCTTGATTTTCTCAACCTGCATCGAAATATATTCGCAGCATACTGGTGGACGATGTGCCGATACGGGTGCTGAGGAACCTGACGGGGCAGGTGGCGGAGTACGAGTTCCCGGCGAAGCGGATGGCCGTGCGGGCGAGCCTGTGGGACGGCTCCGACTGGGCGACGGACGGCGGCAGGACCAAGATCGACTGGGGCCGCGCGCCCTTCACGGCGGGGTTCCGGGGCTTCGACGTCGACGCCTGCGACAACGCCAGCTCGACGCCGTGCGACTCGACGGACCTGTGGTGGAACGCCCGCAGGCACAGGCGGCTGTCCGTCCGGGAGCAGGCGGCCTACGAGAACGTGCGGAGGACGTACATGAACTACGACTACTGCGCCGACAAGGATCGGTTCCAGAACGGCAAGCTGCCGGTCGAGTGCAGCTACACTACTTAG

>HvXTH11

ATGGCGTCCAGCTCATCGTGCCCTCCTCCGTCGCCGCGCCCCTCCCGCCTCCTCCCCGTGCTCGTCGCCACGGTCGTCCTGCTTGGCCGCGGCGGCGAGGCCAGGCAGCCGGCGCCGCTCCACGGCGTCGTGCGGTCCATGGCCTTCGACGAGGGCTACACCCAGCTCTTCGGCAGCGGCAACCTCGCCCTCCGCCGCGAGGGCAAGCGCGTCCACCTCGCCCTCGACGAGTCCACCGGTGAGTGCTGCCTTGCCCGGCTTAGCGCCCCCGTTTCTGCGATTTCGATGTAATATGCGGGTGCGCATTGCACTTGTTCGTTTCACGAACGGGTCCCTCCACTTGAGCCTCCTAAGGTGGCAGGAGGGCGGTTCTGTGATTTTGCCAAGTTCAGGGTGTTGTTCACAAGAACATGTCTTCGTCTTCCTCTTCCCTGTGCAGGCTCCGGGTTCGCCTCCCAGGACCGGTTCCTCCACGGCTTCTTCAGCGCCGCAGTGAAGCTCCCTGCCGACTACGCCGCCGGCGTCGTCGTCGCGTTCTACGTACGTGCGCCGTCTCCTCACCATCTCCGCTACCAAACCGCCGCCCGTAAAGTTAACATCCGTGACGAAATCATGAATCACTCGTTCCGAAGCTAGCTAAAATGAGATGCTTTCTTTGTTTGTGTTCTCGCATGCAGCTGTCGAACGCCGACGTGTACGAGAAGACCCACGACGAGCTGGACTTCGAGTTCCTGGGCAACGTGCGCGGGCGCGAGTGGCGGGTGCAGACCAACGTGTACGGCAACGGCAGCACCGGCGCCGGCCGGGAGGAGCGCTACGACCTCCCCTTCGACCCCACGGACGACTTCCACCACTACTCCATCCTCTGGACCCAACACCGCATCATGTGAGGCACCGTCTCATCTCATCACATCCTTCCCCATCTGCGTTCCACCGTTCATTCCTCTGCTTCTCTCGTTTCCATGTGCTTCCTGGCGGAACAAGCGCACCATAGAAATTAATTTGCTGGGATTTGGATTCCTGGGCGCTCGTGGGGAGGGCCAGGCAAATATTGCTCCTTTTCTTGCTCTGCTTTTTGGCGTGTTTCTTGCTCTGCCCGCTGCTCGTGTCACCACCCTTCTGACCTTTGCAAATTTGGGTCATTCCGAGATCTTTGCGCCTGTTTCAATGCGCATTGCCTGGATTCTTTTACCAAAAGAATCCATGACGTTTCTGCACATAAAGAAATAGAGAAGAAAAATAATTTATAATCTGTAGTTTCTCTTTTAGTGGACAAATCCATGGGAGGGGAGCTGCACTGACATTTGTGTGCTGGCTGCAGATTCTACGTTGATGAGACCCCGATCAGGGAGGTGGTGAGGACGGAGGCCATGGGCGCGGCGTTCCCCTCCAAGCCCATGTCCCTCTACGCCACCATCTGGGACGGCTCCGCCTGGGCCACCCTCGGCGGCCGCTACAGGGCCAACTACAAGTACGCGCCGTTCGTCGCCGAGTTCGGCGACCTCGTCCTCCACGCCTGCCCCGTCAACCGCATCTACCACTCCGCGGCGGCGGCGTGCGGCACGCCCTGGTACGAGCCTGTCGCCGCCGCCTTGTCCGGCGAGCAGCGCGCGTCGATGTCGGCGTTCAGGCGCGGGCACATGTCCTACTCCTACTGCCACGACCGCCGCCGGTACCCGGTCGCCCTGTCAGAGTGCGACGTCGCCGTGCTCCCGCGCCTGTTCGGCCCGGACGGGATGAAGTACGGCGGCGACCGCCGGCACCGCCGCGGAGGGCGCGGCCGCCGCTCCGACGTCGTCATGTGA

>HvXTH3

ATGAAAGCACCCTCTGGTCTCGGTCTAGCCTATAAGAAAGCTGTGTCCTGTGCCTTGTGCTTTGCCCCGGACCAAAGCATTAGCACGCTCCTCCACAGTCCTCCCCTCTGCCTGCCCCTGTGTGGCTTTGCCGTTTCCCGTCCCACATTCGTCGTCGGCGGTACAGTATTCGTTTCCTCTTGGGGGTGGGGAGCGATGGGACCTTGGAGGCGTCCGTGCGTCGGCGCTCTCCTGGCGTGCGCCGCCATTGCGGCTTCTTGCTGCTGCTTCCAGCTCCAGGGCGCTGATGCGGCGGCGAGCCCGTCGTTCGGGGACAACTTCGAGATCACCGGCGCCAAGGACCACGTCAAGACCTCCCCCGACGGCCAGACGTGGTACCTCTCCCTCGACAACAAGACGGGTATATTGATTGGCGCACGCACGCATGCATATAGGAGAGGTAAAAGGATCGTCGTCTCTTGTCTGAGCTTCTCTTTGCTTGCTTCTTCCAGGCGTCGGGTTCCAGACGAAGCAGAAGTACCTGTTCGGGTGGTTCAGCATGAAGCTCAAGCTCGTCGGAAACGACTCCGCCGGCGTCGTCACCGCCTACTACGTACGTACTACTGTACTTACTATCCGGTATCCTTTTCCCAGCCCGGCAGGCGGCAGCAGCGCCCGGCCGATTAAACCAACTCGATGGAAGGGAAGGGAGGAAACCAAACTAGTAACATGTTAATGGAGTGCGTTTGATCATCATCAGATGTGCTCGGACCTTGACGCTGCGCCGGAGCGCGACGAGCTGGACTTCGAGTTCCTGGGCAACCGCACCGGCGAGCCGTACATCATCCAGACGAACGTGTACCGCAGCGGCGTGGGCGGGCGGGAGATGCGGCACTCGCTGTGGTTCGACCCCACCGCCGACTTCCACAGCTACTCCATCCTCTGGAACCCCAAGCAGATCGTGTAAGCAACCCATGGATTCCTTTGCTGCAACTTGCAATCAATTAAGCTTCCGATTGATCTAGCGTTAACGGAGAGTGTAATCTGGGATGGGAGGGTGGGGTTGCCGGCCGGATTTAGTAGCTGCGGGGGCAGTTGAATTGAATGGATCCACCCACTCACCCCTGCATTTGGGTCACTCCGAGTCCGATAGATAGATAGATACCCACTGGACCCGACCCTTGGACTTGGTACGTGCTAGTAGCAAAACTAGGCCGTGTCGCCGTGCTCTTGATACGTGCAGCACCCTGTTTGGGACCAAACAATGCGTGTCGCCGTGCACTGTGTGTACATTGTAGACTGTACCACATGCCGCCCAATCTTCTTCTTTTCTTTCCTCTCTTCTCGCGCGGCCTCGGATGCACAGCATGTACTACGTATACTGCTGAAAGAGAAACTTTCCGCTCTCCAAACTGCGGCAACAGTGGCGTGGGCGCACATGCTTTCGTCCCGTCCGTGCTCGCTTGCTGTAGTATATTATATAGTGTACTGGGATCGAGATCCGACTTGCATTTGCAGCTGGCCAGCTGCCAAGTTGCCATTGCCTTCACTGCGAGAGACATCGTCGCATCGAAAAATTGATGTCGATATTTTATGTACTCTACTGCTGTTTGCAGGTTTTTCGTGGACAAGGTGGCGATCAGGGAGTACCGGAACTCTGCCAAGCCCAACAAGTTCTTCCCGATCATGAAGCCCATGTACGTCTTCTCCAGCATCTGGAACGCCGACGACTGGGCGACGCGCGGGGGCCTGGAGAAGACGGACTGGACCAAGGGGCCCTTCGTCTCCTCCTACAGCGACTTCACCGCCGACGCCTGCGCCTGGCCGTCCGGCCCGGCCCCGCCGGCCTGCGCGGCCGCCACCGGGGACAGCTGGTGGGACCAGCCGCCGGCGTGGGCGCTCGACGACGGCCAGCGCCGGGACTCGGGCTGGGTGGCCAGGAACCTCGTCATATACGACTACTGCGGCGACCGCAAGAGGTTCCCGACCGTGCCGGAGGAGTGCGCGCTCAGGACCACGACTAGCTAG

>HvXTH2

ATGGGAAAGCCGGGGGCACTGGTTCCAGTGGTAGCTCTAGCTTTTGCGTTGGTTCTTGGCCTCGAGCTCGTGTCCGGCGGCAACTTTTACGAGGAGTGCGACGCTACGTGGGAGCCCCAGAACTGCTGGACCTACGACGGCGGCAACAGCCTCTCCCTCGCCCTCGTCAGCAACTCCTCAGGTCTTTCTTCAACCTCACAACTTCTGTATTTTTCATGGAGTTCTCCTCTGAATGAATCAATGTGTGGGTGCTCAGGCTCGATGATCCGGTCCAAGAGGCAGTTCATATACGGAACGGTGTCGACCATGATCCAACTCGTCAAGGGCGACTCCGCCGGCACCGTCACTACATATTACGTAAGAGATGGCTGCTCTGCTCCTAATCGATCATACTCTACGTACATATCTGCATTGACGAACCCTTCGATTAATTCTTCAAGGACTAATTGATATACTGTTGTTCGATTGACTGCATGCAGACATCGTCGGTGGGGGACGACCACGACGAGATCGACTTCGAGTTCCTGGGGAACGAGACGGGGCAGCCCTACACGCTGCACACCAACGTCTACGCCGCCGGCGTCGGCGGCAAGGAGATGCAGTTCCGCCCCTGGTTCGACCCCACCGACGGCTACCACAACTACACCATCGCCTGGACGCCCTGCGCGGTCGTCTGGTACGTGGACGGGGCGCCCATCAGGGCGTTCCGCAACTACGAGCGCACCCACGGCGTGGCCTTCCCGACGACCCGCCCCATGCACGCCTATTCCAGCATCTGGGCGGCCGAGGACTGGGCCACGCAGGGCGGCCGCGTCAGGGCAGACTGGACCCGCGCGCCATTCGTCGCCAGCTACCGCGGCATCGACCTCGACATTTGTGAGTGCTACGGCGGCGACTGCGTCTACACCTGCGCCGGGGCGTTCCGGGGCTGCGGCGGGCTCACCGGAGACCAGCGGGGGAAGATGCAGTGGGTGCAGGACAATTACAGGATCTACGACTACTGCGCCGATCACGAGGCCGGCAAGGTGCCCGGCGTCGAGTGCAGCCTGCCGCAGTACTGA

>HvXTH14

ATGGCGCCAAGGTCAGACCTCCTCGCCGCGCTAGCGTTGGCCCTCCTCGCCGCGAGCGTCCTTAGTACGGGGGCCAAGGCCGACTTCGACGACCAGTTCGAGGTGATCGGCGACCGCGACCACATCGGGTACCGGGACGACGGCAACGACAAGGGCCAGGAGTTCTCGCTGGAGCTCGACCAGGAGTCCGGCTCCGGCTTCAAGTCCAAGGCCAAGTACCTCTTCGGCGAGTTCCAGGTCCGGATGAAGCTCGTCGACGGCAACTCCGCCGGCACCGTCACCTCCTTCTACGTACGAATTAAGCCATCTCATCTTCGTCTTTGTGCTGTGCTGTGGTGTGCTGGAGCCTGGATCTAGCTTGGTTGCTAAATAGTTAAAATGTTTGTTCAGCTGACCTCCGGCGAGAGCGCCACCCACGACGAGATCGACATCGAGTTCATGGGAAACTCGAGCGGCGACCCCTACGTGATGAACACCAACGTCTGGGCCAGCGGCGACGGCAAGAAGGAGCACCAGTTCTACCTCTGGTTCGACCCCTCCGCCGACTTCCACACCTACAAGATCACATGGAACCCAAAGAACATCATGTAAGAATATGCCTCATTTCCCTCCCAACTAATCACGGATTAACTCCAACTAATTCATGGCATGGCAAATTGGATGTCAGATTCGAGGTGGACGGCGTGCCGGTGAGGACCTTCAAGAAGTACGACGGCCTGCCGTTCCCGTCGGCGCGGCCGATGACGGTGCACGCGACGCTGTGGGACGGCAGCTACTGGGCGACGCAGCACGGCACCGTCAAGATCCACTGGCGCCACGACCCCTTCGTCGTCCCCTACCAGGGCTACCACGCCAACGGCTGCGTCCACGACAAGGCCACCAACAAGACCTCCTGCCCCGCCGGCAGCGACGCCTGGATGCACCGCGAGCTCGACGACGGCGAGCTCAGCACCGTCGCGTGGGCCGAGCGCAACTGCCTCTCCTACAACTACTGCGCCGACGGATGGCGCTTCCCCAAGGGCTTCCCCGGCGAGTGCGGACGCAAGTGA

>HvXTH12

ATGGAGATGACGGCGAGGTTCTTGGCCGCGGCGGCGGCGTGCGTGTGGCTGGCGGCGGCGGCCTCCGCCTTCGACGTGCCGACCGTGGCCTTCGAGGAAGGGTTCTCGCCGCTGTTCGGGGACGGCAACCTCGTGCGCGCGCGGGATGATAGGGCCGCCCGCCTCTTGCTCGATCGCCGCTCCGGTAATCCATCGAGACTTTCTTTGTTTTTCTTACGTATTGCTCTGTTCCTGTTGTTCTTATGGCCCTCCTTTGTGTGTTATTACAAAAAAAATACAGGTTCGGGGTTCATCTCCTCGGATTACTACCTGCACGGCTTCTTCAGCGCGTCCATCAAGCTGCCCCGGGACTACACGGCCGGCGTCGTCGTCGCCTTCTACGTGAGTGCTTCAACACTGCCCCCGATTATCTCGTGAGCGTTTCTGTCCAAAAGTTAATTAAGCAGCGGTTTTAGCTTAGGTTATTATTATCGTCCGTTAATCCATGGCCTTGTTTCCAACCGTTGGTCCTTGCTTCGTCAAACGTCACATGGGCATGGCGTGTCAGCTGAGATTTACTACCTGCCATGCCACTGCTCATCCTCTTAAAGAGGAAAAACCATGGCAAGTTGCACACCCATACTCTGGCTACTGTGGCGCAGAGTAGGCAGGCAACAGGGCGTGCCACATGGGCTCGTTCGTTTTTGGTCCTTCACGGCCACAAAAAGGCTGCGCCGCCTGCTCCTCCGGAATAAATGTTGCCATCCATCCCTCTCACCCGCTCTCACCCGCCCGCATTCATCAGCCTCACTAAAACAAAAACAAAATCAAAACAAAGTTATTAGTAATACGTGACACCGTGACAGTTCTAAAGATTGTACGTGTGTATTAGTATTTTTCTGATGTGCGTGTGTAATAAATAAACTAAAAAAATGTGTGTAATGTTCGTGTTGCAGCTGTCGAACGGGGACGTGTACGAGAAGACGCACGACGAGCTGGACTTCGAGTTCCTGGGCAGCCGGTGGGGCGGGCAGTGGCGGGTGCAGACCAACGTCTACGGCAACGGCAGCACCAGCCGCGGCCGGGAGGAGCGCTACCTCCTCCCCTTCGACCCCACCCTCGCCGCCCACCGCTACTCCATCCTCTGGGCCCCCACCCACATCATGTACGCAGGCATCATCACTCCCACTCCCACTCCCACTGCCAGTGCACGTAGCCGTAGAATAGCATGGTGACTGACTGACGTGACGACGTACGTGCAGATTCTACGTGGACGACACGGCGATCCGGGAGGTGGTGCGGCACCCCGGCATGGGCGGCGACTTCCCGGCGAAGCCCATGGCGGCGTACGCCACCATCTGGGACGGCTCCGCCTGGGCCACGGAGGGCGGCAAGTACAAGGTGAACTACAAGTACGCGCCCTTCGCCTCCGACTTCTCCGACCTGTCCCTCCGCGGCTGCCGCGTCGCCGACCCGGCGTCGCCGGCGCTGCGCCTCGCCGGCGGCGACGGGTGCGACCTCCTGGGGCTCATGACGGCCGACTACGCGGTCATGACCCCGCAGAAGCGCGCCGCCATGCGCGCGTTCCGGGCGCGCCGGATGACCTACACGGTGTGCTACGACGCGGCGCGGTACGCGGCCGGCCCCTTCCCGGAGTGCGACAACTCGGACGAGGAGAGGGGCACGTTCTGGGCGTGGGGCGAGTCCAAGACCGTCGTCATGAAGACGCGCGGCCGCGGCCGCCGCGGCCGGGGCAGCAGGGCCGGCGCCGGAGCGAGGGGCCGCGCCGGCGCGGCGAGCAGCTGA

>HvXTH10

ATGGCGATGATGCAGATTAGGCGGCCGCATGATGCCATCTCACATCTCATGGTGATCGTAGTAGGAGCTGTGATACTGCTGCAAGGTGAGGCGCAGCCATCCCCTGGGTACTACCCGAGCTCCAAGGTGAGCTCAACGCCATTCTCGCAGTGGTACAGCACCCTGTGGGGGCCGCAGCACCAGTCTCTGTCGCCGGACCAGACCGCCCTCACCCTCTGGATGGACCGCAGCTCAGGTCAGTAACCTTCTTTTTCTGCATGATTCTTGATTAATCACATGTAGTACGTGGTGCTCATGTCTCTGTGCAACAGTTGGATGAGTAATCAGTTTCACCTAACTAATCATGCTCCTAATAATTAATTAACTCCAGGCAGCGGGTTCAAGTCGAAGCGGTCGTACCGGAACGGCTACTTCGGCGTCTCCATGAAGGTCCAGCCCGGCTACACCGCCGGCGTCAACACCGCCTTCTACGTCAGTACTTTTCCACCTCCGGCGACCATGCATTTTTGCTTCTTGTATCCATCTCAAGTTCTTAGCTGACGTACGTGAGCAAAAATGTATATATGGTGCAGCTGTCGAACAACGAGGTGTACCCGGGGTACCACGACGAGATCGACGTGGAGCTGCTGGGCACGGTGCCCGGCGAGCCCTACACGCTGCAGACCAACGTGTACGTCCGGGGCACGGGGGACGCCCACCCCATCGTCGGCCGGGAGATGCGGTTCCACCTCTGGTTCGACCCGGCCGCGGCGTTCCACCACTACGCCGTGCTCTGGAACCCCGACGAGATCGTCTTCCTCGTCGACGACGTGCCGGTGCGCCGATACCAAAAGAAGGTGGAGGCCACGTTCCCGGAGCGGGAGATGTGGGCGTACGGCTCCGTCTGGGACGCCTCCGACTGGGCCACCGACGGCGGCCGCTACAGGTCCGACTACCGCTACCAGCCCTTCGTGTCCGGGTTCAAGGACTTCAAGGTCGCCGGCTGCGAGGTCGGCGCGCCGGCGTCGTGCCGCCCCGTGCCGGCGGGGCCCGGAGGCGGGCTGAGCGCGCAGCAGAGCGCCGCCATGAGCTGGGCGCAGCAGAGGGCCATGGTCTACTACTACTGCCAGGATGGATCCAAGGACCGCTCCAACTACCCAGAGTGCTAG

>HvXTH13

ATGGCGCCGTCGTTGCCGTCATCCTCTTCTTGTTGGCATTCCGCGCTGCTGGTAGCCATGTTGGTGCTTGTGGTGGTCATGGATCAGGTGGCCATGGCGTACCTGGACGACGACATCGAGGTGGTGTGGGGCGACGACCACAGCTTCTTCTACATGGACGACGCCGGCGACGACGAGATCCTCGCGCTCTGCCTCGACGAGACCCACGGCTCGGGGTTCCACACCAAGGAGGCCTACCTCTACGCCCGCTTCGACGTCGACCTCATGCTCGTCCCCGACAACTCCGCCGGCACGGTCACCACGCTCTACGTAAGTCGATCGACCTTCCACCACCGCCACGCCAGTGCGCCATGCCAGGGCGCGGTCACCGTGCCCAGCCCACGGCACGTTGGCTGTGAATTGACGTATGCATGCGTGAATTTGATCGGTCGATGCGCGCAGCTGATGCCGGAGGACGTGCCGTGGGACTACCACGACGAGGTGGACCTGGAGTTCCTGGGCAACGTCACCGGCGAGCCCTACACGCTCCACACCAACATCTTCGCCAACGGCGTGGGCAACCGCGAGGAGCAGTTCCGCCTCTGGTTCGACCCCACCGCCGACTTCCACACCTACTCCATCGACTGGAACCCCAAGCGCATCACGTAAGCAAATCTATCAGAGCTCAGAGATCAACCTGCAGATACAGTATAGCTCATCGACGGAGAGCTCAAATCAACCATATGGCTGGTGCCATCAGGATCCTGGTGGACGGCGTGCCGATCCGGAGCTTCAGGAACAATGAGGAGCACGGGGTGGCGTTCCCGACGTGGCAGAAGATGCGGCTGCACGGGAGCCTCTGGAACGCCGACGACTGGGCGACGCAGGGCGGCCGCGTCAAGACGGACTGGTCGGGGGCACCATTCTTCGCCCGCTATCGCAACCTCCGGGCGTCGTGGTGCCGGCCGTCGCCGGGGGTGGCGTGGTGCGGCGACGAGCCGCCGGGGTCGACGTGGTTCGAGCGCGGCCTGGACGCGGCGGCGCTGAGGCGGGCGCGCGACGCCCACATGATCTACGACTACTGCAAGGACCTCCAGCGGTACAAGGGGTCGGGGCTCCCCAAGGAATGCGTCGTGGACTGA

>HvXTH17

ATGGCTCGCATGGGGGCGTCGGTGCTGGTGATCCTGCTCGCCTCTTGTGCCCTGGCGGCGGCGAGCTTCGACAAGGAGTTCGACGTTACCTGGGGTGACGGGCGCGGCAAGATCCTCAACAATGGCCAGCTGCTGATGCTGGGGCTGGACAAGGTCTCCGGCTCCGGGTTCCAGTCCAAGCGCGAGTACCTCTTCGGCAAGATCGACATGCAGCTCAAGCTCGTCCCCGGCAACTCCGCCGGCACCGTCACCGCATATTACGTAAGCAAACATCCTTACATTCCTCTGCTTCTCGGTCTCCATCCGATGACACGGTTGCCTAACTCGATTGTTTTGGTGTGTATGAATGTACGTGCAGCTGTCGTCGCAGGGTCCGACGCACGACGAGATCGACTTCGAGTTCCTGGGCAACGTCACCGGCGAGCCATACACGCTGCACACCAACGTGTTCACGCAGGGGCAGGGCCAGCGGGAGCAGCAGTTCCGCCTCTGGTTCGATCCTACCAACGACTTCCACACCTACTCCATCCTCTGGAACCCGAAGCACATCATGTAAGCTGGTCCCTCGCCATTCCTCGACCTTCTTTTCTCTTTCAAAACGCCGGCAATCTCATGGACTGACTGATTTGGTGGGGTAAACAATGCAGCTTCTTGGTTGACGACATGCCGATCAGGGACTTCAGGAACATGGAGGGAAAGGGGATCGCCTTCCCCAAGAACCAGCCTATGCGGCTGTACTCCAGCCTCTGGAACGCCGACGACTGGGCGACACAGGGTGGCCGCGTCAAGACCGACTGGTCCCACGCTCCGTTTTCCGCCTCCTACCGCGGCTTCAAGGCCGACGCGTGCGTGGTGACCGTAGGTGGCCGGCCGCGCTGCGGCGCCAGCATCGGCACGGACGCGGCCCCAGGGACCGGCGGCGCGGCCGCGGTCGGCGACTGGTACAACCAAGAGCTGGATCTTACGCGGCAGCAGCGCATGCGTTGGGTGCAGAGCAATTACATGATCTACAACTACTGCACTGACCCCAAGCGCGTCGCCAAGGGCGTCCCTGCCGAGTGCTCCATGTAG

>HvXTH21

ATGGCGTCCGGTCCCAGTAGAACAGTCCCGTGCTCTGTGCTGCCACTGCTGCTGCTGCTCGCCGGCGTGGCCCGCGCGGCCGGCAACTTCTACCAGGACGTGGACATCACGTGGGGCGACGGGCGCGGCAAGATCCTCGGCGGCGGCGACCTCCTCACGCTGTCGCTCGACAGGGCCTCCGGCTCCGGGTTCCAGTCCAAGAACCAGTACCTGTACGGCCGCTTCGACATGCAGATCAAGCTCGTCCCCGGCGACTCCGCCGGCACCGTCGCCACTTTCTACGTACGTTTTCTTGCACGACCGCACGTGTGCACGTGCGTGTTGACTCGCATCATGCATGGGGAGTACTGTACTGATTCGAGCCTACTATATTGCAGCTGTCGTCGCAGGGGTCGGCGCACGACGAGATCGACTTCGAGTTCCTGGGCAACGCGAGCGGGCAGCCCTACACGGTGCACACCAACGTGTACAGCCAGGGCAAGGGCGGCCGGGAGCAGCAGTTCCGCATGTGGTTCGACCCCACCGCCGACTTCCACACCTACTCCGTCCTCTGGAACCCCACACACATCCTGTACGTAGTATGCAGACGGCGTCGGTCTCTCTCTCTCTCTCTCTCTTCAACCTCTTGTCGACGCAGGACAAGACCTCATGCATGAATGAACCCGTTTCTCCGCATGCAGGTTCTACGTGGACGGGACGCCGATACGGGAGCACCGCAACCGGGAGGCGGCGACGGGGGTTCCCTACCTGCGGAGCCAGGCGATGAGGGTGTACGCGAGCGTGTGGGACGCGGAAGAGTGGGCGACGCAGGGCGGGCGGGTGAGGACGGACTGGTCGCGGGCGCCGTTCGTGGCGTCGTACAAGGGGCTCGCCGCGAGCGGGTGCGCGTCGCAGGACGCGGCGGCGTGCGCCAACTCCAACGGCGCGTGGATGTACCAGGAGCTGGACGCCACGGCGTTGGACCGCCTCCAGTGGGTGCAGAAGAACTACATGATCTACAACTACTGCACGGACACGTGGAGGTTCAAGGACGGCGCCCCGCCCGAGTGCGCCAGCAAGTAG

>HvXTH24

ATGGGCCAGGCTAGGGCTTACCTCCTAGCCTCCCTAGCGGCGTTCTACCTCGTCGCCCTGGCCATCCCCCAGGTCACCGCCGACATGACCGACGAAGTCAATCTCCTGTGGGGCAACTGCAAGGTTCAACGCGATGGCACCGGCCGACAGACTGTCGCGATGAGTCTCGACCGCTGGACGACTTCAGGATTCTCCTCGAAAATCAAGTACCTATTCGGGAGGATTGACATGGAAATCAAGCTCATGCCCGGGAACTCAGCCGGCACAGTGACAACATTTTATGTAAGTGATTCTGATTAACGTCGCTCATGTCAACTTGTTAATACTAGGACTAACCATCCTTCATTGCAATCTGGGAATAGATGATGTCAGAGGGACCATGGCAATTCCATGATGAAATCGACCTTGAATTCTTGGGGAACAGCACCGGCAACCCCTACACCCTGCACACCAACGTGTATGCCAGAGGTGTAGGCAGCAGAGAGAAGGGGTACCGGCTTTGGTTTGATCCCTCCCAGGACTTCCACACCTACAGCATCATTTGGACCCAACAATACATCAGGTGAGCCCCACTCCTATCTACCCCATTCGGGTTCAACATGCCTGCTTAGATAAACTACCCCCTCCTATCTAAATAGCATTTAATTCCAAATGAAGCATGAATGCTCAGTTTAGTCAGACGTGTTAAATAATTTTTAGACCAAACAAAAAGGTATTTGCAAGCACTAAGCCTAGCTCATATGGATGTTGATTGGTTGCAGATTCCTGGTCGATAACAAGCTGATCAGGCAGATCAAGAACAAGATGATGAATGGTTCCCCCTATCCAAACTATCAACCAATGAGGGTGTTCAGCACCATCTGGAATGCGGATGACTGGGCGACACAGGGTGGGCGGGTCAAGACCGACTGGACACAAGCGCCATTCACAGCATACTTCCGGAACTACAAGGCCACCAGCTGCTCTCAAGGCCAGAACTCCAACGTCTGCGGCCAGAGCTCCCCCAACGGTTTGTTCAACCAGCAGCAGGACCAGATGCAGCAACAGCAAGTGAAGGAGGTGGATGCTAAATACAAGGTCTATGATTTCTGCGATGACTCAAAGAGGAGGATTGGGTCCTCCGAGGACTGTCAATCACAGTAG

>HvXTH23

ATGAGGACGGTGGAGCTCGGCATTGTGGCCATGGCGTGCCTCGTCGCGGTGGCGCGGGCCGGCAACTTCTTCCAGGACTCGGAGATGTCCTGGGGGGACGGCCGCGGGAAGGTCGTCGACGGCGGCCGCGGGCTCGACCTCACGCTCGACAAGACCTCCGGCTCCGGCTTCCAGTCCAAGAGCGAGTACCTCTTCGGCAAGATCGACATGCAGATCAAGCTCGTCCCCGGCAACTCCGCCGGCACCGTCACCACCTTCTACGTAAGCTTCACTAGCGTACATTCTCGGTTACTCGCCGTTGCCGTCTCCGTATGTGATGGAAGCTGATGGTTTTTTGGCCTGTGCGTGTGTGAATGTGCAGCTGTCGTCGCAGGGGACGGCGCACGACGAGATCGACTTCGAGTTCCTGGGTAACGTCACCGGCGAGCCCTACACGCTGCACACCAACGTGTTCGCGCAGGGGCAGGGGCAGCGGGAGCAGCAGTTCCGCCTCTGGTTCGACCCCACCAAGGCCTTCCACACCTACTCCATCATCTGGAACCCGCAGCACGTCATGTAAAGCCTCCTCCCGCTGCCTTCACGGATCCTTTTCTTCTCTTCTTCCGCTCTGGAGACAGAGTGTTTGGGCTTGTCGCTGACGTGAAGGGTTTCGGTGACGGGCGTGCAGATTCGCGGTGGACGGCACGGCGATCAGGGACTTCAAGAACCACGAGGCGCGGGGCGTGTCGTTCCCCAAGAGCCAGCCGATGCGGCTGTACGCGAGCCTGTGGAACGCCGACGACTGGGCCACGCAGGGCGGCCGGGTCAAGACCGACTGGAGCAAGGCGCCGTTCGTCGCCTCCTTCCGCAACTTCAACGCCGACGCCTGCGTCATGTCGGGCGGCGCGCAGCGCTGCCCCGCCGGCACCATGGAGGCCTCGGCGGCCGGCGGCGGCAGCTGGTGGAACCAGGAGCTCAGCGGCATGGGGTACCGCCGCATGCGGTGGGTGCAGAGGAAGTTCATGATCTACAACTACTGCACCGACCCCAAGCGGGTGGCGCAGGGCGTGCCCGCCGAGTGCAAGCTCCGCTGA

>HvXTH18

ATGGCTCGCATGGGGGCGTCGGTGCTTTCGATCCTGCTCGCCTCTTGCGCCCTGGCGGCGGCGAGCTTCGACAAGGAGTTCGACGTTACCTGGGGTGACGGGCGCGGCAAGATCCTCAACAACGGCCAGCTCCTGACGCTGGGACTGGACAAGGTCTCCGGCTCCGGGTTCCAGTCCAAGCACGAGTACCTCTTCGGCAAGATCGACATGCAGCTCAAGCTCGTCCCCGGCAACTCTGCCGGCACCGTAACCGCCTACTACGTAAGCGACCCAACATCCATTCATTCCCCTGCTTCGAGGTCTTGACAAGATTGCCTGACTCGATCGGTTTGGTGTGTATGAATGTACGTGCAGCTGTCGTCGCAGGGGCCGACGCACGACGAGATCGACTTCGAGTTCCTGGGCAACGTCACCGGCGAGCCCTACACGCTGCACACCAACGTGTTCACGCAGGGGCAGGGCCAACGGGAGCAACAGTTCCGCCTCTGGTTCGATCCCACCAACGACTTCCACACCTACTCCATCCTCTGGAACCCAAAGCACATCATGTAAGCTGGTCCATCGCCATTGCCAAACCTTCGTTTTCCTTTCAAGAAGGCTGGCCATTTCCATTTGTGGCTTCTGCGACACATGGACTGACTGAGTTGGTGGTGTAAACAATGCAGCTTCATGGTGGACGACATGCCGATCAGGGACTTCAAGAACCTGGAGGGGAAGGGGATCGCGTTCCCCAAGAACCAGCCCATGCGGCTCTACTCCAGCCTCTGGAACGCCGACGACTGGGCCACGCAGGGCGGCCGCGTCAAGACCGACTGGTCCCACGCGCCGTTCTCCGCCTCTTACCGTGGCTTCAAGGCCGACGCGTGCGTGGTGACCGCGGGCGGCCGGCCGCGCTGCGGCGCCAGCATGGGCACGGAAGCGGCCCCGGGCACGGGCGCTTCCGGCGCGGCCGGCGAGTGGTACAACCAGGAGCTGGACCTGACGCTGCAGCAGCGGATGCGGTGGGTGCAGAGCAATTACATGATCTACAACTACTGCACTGACCCCAAGCGCGTCGCCAAGGGCGTCCCCGCCGAGTGCTCCATGTAG

>HvXTH1

ATGGCGAGGCCGTCCTTCTCCCTCCACCTGTGCCTGGCCGTTCTGGCCTTGGCCGCCGCCGCGTCGGAGGCCGGGTTCTACGACCAGTTCGACGTGGTCGGCTCCGGCAACAACGTCCGCGTGAACGACGACGGCATCGCCCAGCAGGTGGCGCTCACGCTCGACCAGGGCAACGGCGGCTCCGGCTTCAGCTCCAAGGACAAGTACCTCTACGGCGAGTTCAGCGTCCAGATGAAGCTCATCGGCGGCAACTCCGCCGGCACCGTCACCTCCTTCTACGTAAGTCTCGTCTTTTTCAGAGGACCAAGCGAGGGAAGACTATGGTCTGACTCTGGCTGACCTGTGCTTGTGTGGTGCACAGCTGACGTCTGGGGAGGGCGACGGCCATGACGAGATCGACATCGAGTTCATGGGCAACCTCAGCGGCGACCCCTACGTGATGAACACCAACGTCTGGGCCAGCGGCGACGGCAAGAAGGAGCACCAGTTCTACCTCTGGTTCGACCCCACCGCCGACTTCCACACCTACAAGATCGTCTGGAACCCCAAGAACATCATGTACGCACCCCGCCCATCCATTCCCCAAACCATCTTGGTCTTGCATCCAAGTTCCATGTCTAATAACTCTTCCATTGATTGATCTTAAAACCAGATTCCAGGTGGACGACGTGCCGGTGAGGACGTTCAAGAAGTACGACGACCTGCCGTACCCGAGCAGCCAGCCGATGACGGTGCACGCCACGCTCTGGGACGGCAGCTACTGGGCCACCCGCCACGGCGACGTCAAGATCGACTGGACCCAGGCGCCCTTCGTCGTCAACTACCGCGGCTACACCTCCAACGGCTGCGTCAGCAACGGCGGCTCCTCCGCGTGCCCCGCCGGCAGCGACGCCTGGATGAGCACGGAGCTCGACGCCAAAGCCCTCGGCACCGTCGCCTGGGCCGAGAGCAAGTACATGTCCTACGACTACTGCACCGACGGCTGGCGCTTCCCCAACGGCTTCCCCGCCGAGTGCTCCCGCCGCAACTGA

>HvXTH6

ATGGCACAGCGCTTTCTGGCTGTGCTCGCCGTCGCCCTGGCGCTCTCGCAGGTCGCCTCAGCTAAGTCCTGGCTCGATAAAAGGTTCAACACCGACGGCACCGTCCGCACCGGATACGACGCCTCGGGCCAGCAGGTGGTTATGCTCAACCTCAACCAGCAATCCGGCGCCGCCGGATTCAACTCCAAGCAGCAGTACCTCTACGGCGAGTTCAGCATCCAGATGAAGCTCATCCCGGGAAACTCCGCCGGCACCGTCTCCTGCTTCTACGTAAGTCCACTTATATATTTGTTCCGTTGATCAGCCTCCGATTGACATAGACAAAAACCTCCCTCTACCGATCTTGAGCTGAACCAGCGTTTACTATTAATGGCAGCTTTCTTCCGGTGACGACGAGTGGCGCGACGAGATCGACATGGAGTTCATGGGCAACTCCAGCGGCCACCCGGTGGTGCTCAACACCAACGTGTGGGCCAACGGCGACGGCAAGAAGGAGCACCAGTTCGACCTCTGGTTCGACCCAGCCGCAGACTACCACACCTACACCATCATCTGGAACCCGGAGAACATCCTCTTCAAGGTGGACAACCTCTTCATCCGATCCTTCAAGCGCTTCGCCGGCCTCCCTTACCCTACCTCCAAGCCCATGAGGCTGCACGCCACGCTCTGGGACGGCAGCTACTGGGCGACCGAGAAGGGCAAGATCCCGATCAACTGGTCCAACGCGCCATTCGTCGTCTCCTACCGCAACTACTACGCCAACGCCTGCGTCAGCGGCGGCGCGTGCCATGCCGGCAGCGACAGGTGGATGAGGAAGCAGCTCGACGGCGACGAATGGGGCACCGTGAAGTGGGCGGAGCGCAGTTACATGCGCTACAACTACTGCGAGGATGGGTACAGGTTCCCGCAGGGGCTTCCCGCCGAGTGCAACCGCTACTGA

>HvXTH5

ATGGCACGGCGTCTTCTCGCTGTGCTCGCCGTGGCTCTTGCGCTCTTGCAGGCCGCCTCGGCCAAGTCCTGGCTCGACAAGAGGTTCAACACGGACGGCACCGTCCGCACGGGATACGACGCCTCGGGCCAGCAGGTGGTGATGCTCAACCTCAACCAGCAATCCGGCGCCGCCGGCTTCAACTCCAAGCAGCAGTACCTCTATGGTGAGTTCAGCATCCAGATGAAGCTCATCCCGGGGAACTCCGCTGGCACCGTCTCCTGCTTCTACGTAAGTTCATGAGCCAAGTTAATTTTGACCAGGACTAACATTAATTAACCTCGCTCTGTCGATCTTCATTTGTCTTGAGCTGAGCCAGCGTTGCTATTGGTTGCAGCTTTCTTCCGGTGACGACGAGTGGCGCGACGAGATCGACATGGAGTTCATGGGCAACTCCAGCGGCCATCCGGTGGTGCTCAACACGAACGTGTGGGCCAACGGCGACGGCAAGAAGGAGCACCAGTTCGACCTCTGGTTCGACCCCGCCGCCGACTACCACACCTACACCATCATCTGGAACCCGGAGAACATCCTGTTCAAGGTGGACAACCTCTTCATCCGATCCTTCAAGCGCTTCGCCGGCCTGCCCTACCCTACCTCCAAGCCCATGAGGCTGCACGCCACGCTCTGGGACGGCAGCTACTGGGCGACCGAGAAGGGCAAGATCCCCATCAACTGGTCCAACGCGCCATTCGTTGTCTCGTACCGCAACTACTACGCCAACGCCTGCGTCAGCGGCGGCGCGTGCCATGCCGGCAGTGACAGGTGGATGAAGAAGCAGCTCGACGGCGCCGAATGGGGCACCGTGAAGTGGGCGGAGCGAAGTTACATGCGGTACAACTACTGCGAGGATGGGTACAGGTTCCCACAGGGGCTTCCCGCCGAGTGCAACCGCTACTGA

>HvXTH7

ATGAGCAATACCTCTACGGTGAGTTCAGCATCCAGATGAAGCTCATCCCGGGAAACTCGGCCGGCACCGTATCCTGCTTCTACGTAAGTTAATGAGCTATCTTAATTTCGAGTACAGAGTTCCTCGAATGTTATTCGATCATCAAACTTTGCAAGCATCGATAACATTTGTTCATAATCATTCCTTCAAAGTTTTAGATTTTTAAAAATGTTTTGTTATGTTTTTTCTTAAGGGGTATAGCTAGAAAAACCACTCTCATATATAATTGATCGCAGCTTTCTTCCGGTGATGGAGACGGGCACGACGAGATCGACATGGAGTTCATGGGCAACTCCAGTGGCCCTGGCCATCCGGTAGTGCTCAACACCAACGTCTGGGTCAACGGCGATGGCAAGAAGGAGCACCAGTTCGACCTCTGGTTCGACCCCGCCGCCGACTACCACACCTACACCATCATCTGGAACCCGGAGAACATCCTCTTCAAGGTGGACAACCTCTTCATCCGGTCCTTCAAGCGCTTCGCCGGCATCCCCTACGCTGGCTCCAAGCCCATGAGGCTGCACGCCACGCTGTGGGACGGCAGCTACTGGGCGACCGAGAAGGGCAAGGTCCCCATCGACTGGTCCAACGCACCCTTCAACGTCTTGTACAAAAACTACTACGCCAACGCCTGCGCCAGCGGCGGCGCTTGCCATGCCGGCAGCGACGGGTGGATGAACAGGCAGCTCGACGGCTCCGAGTGGGGCACCGTCAAGTGGGCGGAGCAAAATTACATGCGCTACAACTACTGCGCAGATGGCTACAGGTTCCCACAGGGGTTCCCCGCCGAGTGCAGCCGCTACTGA

>HvXTH4

ATGGCGCCGGCATTGCCTTGTAGCAGGCCAAAGCTGCTGCTCCTGTGCGTGGCCCTGGCCTTCCTCCTGGCCGTGGACGTGGGCAGGGCGGACATCTACAAGGACATCCAGATCATATGGAGCGCGGACCACACCTACTACTTCATGGACGGCGACAGCGAGGCGCTGGCGCTCTCGCTCGACTTCAACCGCGGCTCCGCCTTCAAGTCCAACGACATGTACCTCTACGCCCGCATCGACATCGACATCAAGCTCGTCGAGGGCAACTCCGCCGGCACCGTCTGCACCGTCTACGTAAGATCTCGATCTAGTAATCAGTTTGCATCAGTGCAGCTTAGCTTGCCAGTGTCACAGTGTGTGTGTGTGCGTCCGTCGAGTTGACTTTTGACATGGGCGGTGTACGCATACAGACCATCTCGGAGGGGCCGTGGGACATCCACGACGAGATCGACCTGGAGTTCCTGGGCAACTCCACCGGCGAGCCCTACACCCTCCACACCAACATATTCGCCTACGGCGTCGGCGGCCGGGAGCAGCAGTTCAAGCTCTGGTTCGACCCAAGCGCCGAGTACCACACCTACTCCATCGTCTGGAACCCCAGGCGCATCACGTAATGAACCTATTTTCTTGCGTGATACTCCGTATATGTTATATGTACGTACGTGGAGCATGTAGGTCGAGGTCGATCGTGCCGATCTTCAGAGAGCACATAGACTGACTGACGTGAACTGGACTTTTAATTGCAGGATCGAGGTGGACGGCGTGACGATCCGTTCCTACGACAACAACGAGGAGCACGGCGTGCCGTTCCCGGCGTGGCAGCAGCAGCGGGTGTACGGGAGCCTGTGGAACGCCGACGACTGGGCGACGCAGGGCGGGCGCGTCAAGACGGACTGGAAGCTGGCGCCCTTCGTCTCCTACTACCGCAACTACAACATCACCTACTGCCGGCCGTCGCCGGGTGTGTCGTGGTGCGGCGCCGAGCCCGCCGGCTCCCCGGTCTTCAACCTCGCCCCCAAGGCGCGCGCCGACATGCAGTGGGTGCGCGACATGGGCTACGTCATCTACGACTACTGCACCGACAGGAGCAACCGGTATAACGACACCACCCGGCCCAAGGAGTGCTCGCTCCCGCCACGGCCATGA

>HvXTH9

ATGGCGTGTCACTTCCTCTTGGCCGTCCTCCTGGCGTCGTCTTCTTGGGTTGCTGCGTCCTCCGGCGCCGCCGCGGACGATGTCATGGTGCCCCGCCCGACGACGGCGGCGGCGCTCACCTTCCGGGAGGGCTACACCCAGCTGTTCGGGGACTCCAACCTGAGGCTCCACGGCGACGGCAAGCGAGTCCACATCTCCCTCGACGAGAGGACAGGTACCCACCATGCCGTGCTTCATCGATCGATCCTCACGTTCTTGTCGCCGCCTTCCGTTCCTCATGGCGCGGCGCCTGCCTCTGTTGGTTTGGTGTATCCAGGCTCCGGGTTCGCGTCGCAGGGCGCGTACTTCCACGGCTTCTTCAGCGCCAGCATCAAGCTGCCCTCCGACTACGCCGCCGGCGTCGTCGTCGCCTTCTACGTGAGTACCCCTGCAGGCCTGCACTGCACCGCGCTTCTTGCAAACGAAACGAACCGACATAATAACGCACTGCTAACAGACTTTGGCATTGCGATCGTTGGATTTTCCGGCAGATGTCCAACGGCGACGTGTACGAGAAGACGCACGACGAGCTGGACTTCGAGTTCCTGGGGAACGTCAGGGGGAAGGAGTGGAGGGTGCAGACCAACGTGTACGGCGACGGCAGCACGGCGGTCGGCCGGGAGGAGAGGTACGGCCTCTGGTTCGACCCCACCCACGACTTCCACCGCTACGCCATCCTCTGGACCAACCGCACCATCGTGTAAGCTAGCCCCTGCTCTCCCTCCCATCTCATCCCCGCCAATCTTACTTACTTGCTCCGTTGCTTGTCATCGATCGGCACCACACTGCATTGCGTGCACGGTTTGCATTTCGCTGCAAGGTCTGATCGGACTGAACTTTGAACAGTAAACTAGTACGGAGTAGTTCAGAACTTCAGACCAGGAGCCTCAAGTGATGGCTTCCGGCACAATGTAGAGTGGTTTTAGCTGCAACAGCCGTAGCCAATTACTTCACCTGGCATTCGATTAGCTCGGATTGCTCCAGGGCTATCACTATACAGGATCAGTGTTGCTTGAACGGCGTTTCTGAATCATTATTACAACCCGTAGGCTGAATTCTGTGGATAATCCTTGTGGATGCCTGCACCACAAGTGTCGCTGTGTTACTTGTGTACTCTGTATATACAAGTTCCAAGCACACATGTGGTACCTGCCAGCAAATTAGTGATCCTTAAAGTACATACCAATATCTTCAGTGCTTGCAGAACACAGTACCATCTCCGTGTCCTTTGATAAAACAAAATAGTGTCAGTACCTGACCTGCGCTCTTTGTCATGGTAGTTCAGCTTAGCAATGTTGCATGTGCTACAACAATGTAGCAAGATAACCTCTGCTGCTAGTATTAGCTTCAGGTAGGAGTACTTTCTGTTGTTTGTTGCTTCTTTCCCTTTCCTTCTCTCCCTTTCTTTTTTCACCCTCATGACATGATTGAGAGAAATTGGCTCCTCTTCTTTACAGCTTACAGATTCCTCATGCCGTTGCCGCTTTAATAATTTCAAGTACCGAGGGCCCGAAATCGAGTAGATGGCCTTGCCGCAACGGGCATGCAGCAGATAGCGCTGCCCCCGCTAGCATGCTTCAAGATTGCTCCCTTGAAAGAAGAAACGTGTCGACATCAAGATCGTTTCTTCTTCCACCTGATCGAGTTCAGACCACGGATCCATGAGCATGACTCACAGTCTTACTCACAGAGTCTCACACAGCTACTCACGCAGCCACACTGCATGTTGGTGTGGGTGAGTAAAGTTGGCATGAGAAATGGGGGGCGTCCGCTTTTACAGTTTCCATCCACTGAAACTGAGCATCGCATTCATTCATCACAGCATGTGCATGCATGGCCATGGCTGCGCCTGAACCTGATCCTTCCACCAGGATCCCCGTGCCGCTGCCTTTTCCTCATGTAACTGCGTCGCGCACCTGCTTTCGACCCTACCCTGCAAGTCCTGTGTGATCACTCGCACGCAGGCCAACTTTCGAATTGATGGGGAAGAAAGCTACCACCACCTCCTCCACTTTTCACCGTGCGTTTTCGCATGAGGCAGCCCGGCCTGTTCAGGTTCACCGTTCACATGCCCAAAGTCCCAACTCGCCCAAGTTTTCTCTGCAAATATCTTCGCATAAATGCTTGCTACCTACCTCTACTTTTCACCAAGTGTTTTCACATGAGCTAACCCGGTCTGTTCAGGTTCAGCGTTCGCATGCTCAAGTTTTTACTGCAAACATGTCCACATGAAATGCTTCAAACTGTGACTAACACGACTCTGGAGTCATGCAATCATGGGGTCTTGCATCGCGCTGATTTGGCGGTGCATTCGCCGTTTTTGTGCGTGCAGGTTCTACGTGGACGGTACGCCGATCAGGGAGGTGGTGAGGAGCGAGGCGATGGGGGCGCAGTTCCCGTCCAAGCCCATGTCGCTCTACGCCACCATCTGGGACGGCTCCAGCTGGGCCACCTCGGGGGGCCGCTACAAGGTGGAGTACAAGTACGCGCCCTACGTCGCCGAGTTCACCGACCTCGAGCTCCGCGGCTGCGCCTCCCATGATCGAGCCCAGCCGGCGTCGTGCGAGCCGGAGGGAATGCCGGCCAGGCAGCGGGCGGCGATGGAGAGGGTCCGGGCGCGGCACATGACGTACGGGTACTGCTACGACCGCGCGCGGTACCCTGCGCCGCTGCCCGAGTGCAGGGTGGGCGCCGAGGCGGCCATGTACCTCCCCTCGGGCGAGGCCAGGTCGTCGGACCGGCGCAGGCACGGCAAGCGCCACCGTCGTGCCGACTCCGCTCTCTGA

>HvXTH8

ATGAAGGCTACCGCGGGGGCCCTCCTCGCCGTGGTGGCCACGGTGCTACTGCGAGGCATCGCGGCAGCGCCGCCCCGGAAGCCGGTGGACGTGCCATTCGAGAAGAACTACGTCCCGACATGGGCGGAGGACCACATCCACTACGTGAACGGCGGACGGGAGGTGCAGCTGTCCCTCGACAAGACCACCGGCACTGGCTTCCAGACCCGGGGCTCCTACCTCTTCGGCCACTTCAGCATGCACATCAAGCTCGTCGGCGGCGACTCCGCCGGCACAGTCACCGCCTTCTACGTACGCCCTATATTCCCTCGCATTCATCTACATCTCGTCCTTGTGCAGTTTCGCTTGTTCGTGTGCAGCCAGCAGCAGTATTTTTTTTTTCTTTTTTGAAAAGGAGGATTGCCCCCGCCTCTCTGCATCACGATGATGCATGCAGCCATAGCCAGCAGCAGTATATATAGTACTCCCTCTTTACCTAAATATTTGTAGTCGGAGAGTACTAGTTTAGTTCTTCCTAACTTCAAGTATTTTTGGTACGGAGGGAGTATAATGTAATCTTTGCATCTGGAATGACGGATGGATTGCATTTGCAGCTGTCGTCGCAGAACTCGGAGCACGACGAGATCGACTTCGAGTTCTTGGGGAACAGGACGGGGCAGCCGTACATCCTGCAGACGAACGTGTTCTCCGGCGGGAAGGGCGACCGGGAACAGAGGATCTACCTCTGGTTCGACCCAACCAAGGACTACCACTCCTACTCCGTCCTCTGGAACCTCTACATGATCGCGTACCTCTTCCATCCCCACTTTCGTATTTTTACTCGTAAAATTATGTTACTTTTTCCTTTCCTTTGCTTTGTTGGTGACAAATGAGAAAACGACTATAGGTAACTCATAGGCGACTAGTACTAGAACTAGATTTAGCATCTGACAGGTAAACTTTTTTTTAAACTATATACAGACGCATACATATCTTATCTCTATCAATATCTTTATCTTTGAAAGACTGAGGCGGAATATCTCCTTCACCATTGAAAACGTATTCTTAATTTTTTTAAAATAAATTCAATAATAATGCAAGCAGCATGACTTTAACCATGATAGGCTCTGTTATTCCAACCATCCAACATCGACCCAGACCCACACGTTGGTTTGCTCTCACAGGTAAACTTTAATGTGCCGTACATGCTTGGAACATGATGTTTTAAAATCTGTACTGCCCAAACAAAGCGTAATATCCGTAACTAGAACAGCAGCTAAGATCGGTGCGCATGTGCAATCGATGCGAATACCAAGGGTGGAGGTCGGTCTGCCCCTGGGCCGCTGCTCCAGGCAAGGCAATTATGCCTCAGCGTCTCATTGGCGCGTGCAATACAGTTCACTTCACCGCGGAATTAGGGCATCTTCAATGGTTGTAAGATAGTTGTTGGTAATTTTGACACATAAGATTTTTGATGATGTGTCAAGCAATAAATGAGGAAAGAGAGGAATGTTGTATGTAAATTAACCAACACCTTTGCACAAGCTCCAATGTAGAATGAGAGAGCACCTTATTTATTACCTTACATCTTATTGAGCAAACTAGATACTACCCATTGGAGTAGTTGTATGTTAAGGTGTTGGTTGATGACATGGCATATTTTATCAACAAGCTAACCAACATACTATTGGAGATGCCCTTACTGCAAAGTTGAACACTGGGCACGATGTCTGACGCGCTGTCTCATAGACACGACACGCCACTACACTACTAGTAATACGTCCTCGCAAAAAAATATCGAAATTTATAGAAGAAAAAAAGGTACTGTTGTGACGTGCCGTGCCGTGACATTGTGCAGGTTCTTTGTGGACGACACGCCGATCCGGGTGTTCAAGAACAGCAAGGACCTCGGCGTGCGGTACCCCTTCGACCAGCCTATGAAGCTCTACTCGAGCCTGTGGAACGCGGACGACTGGGCGACTCGGGGAGGGCGGGAGAAGACGGACTGGTCCAAGGCGCCCTTCGTCGCCTCCTACCGGGGCTTCCACGTCGACGGCTGCGAGGCGTCGGCGGAGGCCAAGTTGTGCGCCACCCAGGGCGCCCGCTGGTGGGATCAGCCCGAGTTCCAGGACCTGGACGCCGCGCAGTACCGCCGCCTCGCCTGGGTCAGGAAGGAGCACACCATCTACAACTACTGCACAGACCGCGAACGATACGCCGCCATGTCGCCCGAGTGCAAACGCGACCGCGACGTCTG
