# Supplementary material for: Genome-Wide Identification, Characterization and Expression Analysis of Xyloglucan Endotransglucosylase/Hydrolase Genes Family in Barley (Hordeum vulgare)
Source: Molecules. 2019 May 20;24(10):1935. doi: 10.3390/molecules24101935 (PMC6572274; doi:10.3390/molecules24101935)
Supplement: Supplementary file 1 [file molecules-24-01935-s001.zip › Supplementary File 4 Table S1. Pairwise identities between paralogous pairs of HvXTH genes.docx]

| **Paralogous pairs** | **Score** | **Identities** | **Similarity** | **Gaps** | **Distance** |
| --- | --- | --- | --- | --- | --- |
| HvXTH6/7 | 935.5 | 164/279 (58.8%) | 174/279 (62.4%) | 86/279 (30.8%) | <100kb |
| HvXTH17/18 | 1513 | 277/292 (94.9%) | 284/292 (97.3%) | 0/292 ( 0.0%) | <100kb |
| HvXTH19/20 | 1544 | 284/289 (98.3%) | 286/289 (99.0%) | 0/289 ( 0.0%) | <100kb |
| HvXTH21/22 | 779.5 | 149/302 (49.3%) | 196/302 (64.9%) | 25/302 ( 8.3%) | <100kb |

**Table S2.** Pairwise identities between paralogous pairs of HvXTH genes.
