# Supplementary material for: Genome-Wide Identification, Characterization and Expression Analysis of Xyloglucan Endotransglucosylase/Hydrolase Genes Family in Barley (Hordeum vulgare)
Source: Molecules. 2019 May 20;24(10):1935. doi: 10.3390/molecules24101935 (PMC6572274; doi:10.3390/molecules24101935)
Supplement: Supplementary file 1 [file molecules-24-01935-s001.zip › Supplementary File 5 The multiple alignment of deduced amino acid sequences of HvXTHs 22-28and 2UWA .pdf]

```
2UWA      η1  α1  β1
          1        10        20        30        40        50
2UWA      MPPNILSIF......LHLLPILMFSSSCL.GQGPPSPGYYPSSQ...ITSLGFDQGYTNL
HvXTH22   ..MAMMQIRRPHDAISHLMVIVVGAVILLQGEAQPSPGYYPSSK...VSSTPFSQWYSTL
HvXTH23   ..MASLSL.......LPAMALLLLAMAVASSDAQPSPGYYPSSR...FRPVAFNRGYSNK
HvXTH24   ...MEMTA.......RFLA..AAAACVWLAAAASAFD.........VPTVAFEEGFSPL
HvXTH25   ..MAAASA...........LLMAALAVFAAAAAAALD.........TSPVPFDAGYAPL
HvXTH26   .....MAC.......HFLLAVLLASSSWVAASSGAAADDVMVPRPTTAAALTFREGYTQL
HvXTH27   .....MAC.......HFLLAVLLASSSWVAASSGAAADDVMVPRPTTAAALTFREGYTQL
HvXTH28   .MASSSSCPPPSPRPSRLLPVLVATVVLLGRGGEARQ...PAPLHGVVRSMAFDEGYTQL
HvXTH29   ...MASSVR......QPWLLLLLVLLPVM.................ATAAVFDDNYAPT

2UWA      η2 β2 β3 β4 β5
          60        70        80        90        100
2UWA      WGPQHQRV..DQGSLTIWLDSTSGSGFKSINRYRSGYFGANIKLQSGYTAGVITSFYLSN
HvXTH22   WGPQHQSLSPDQTALTLWMDRSSGSGFKSKRSYRNGYFGVSMKVQPGYTAGVNTAFYLSN
HvXTH23   WGPQHQTVSGDHSAITIWLDRTCGSGFKSKHAYRNGYFATRIKLPAGYTAGTNTAFYLSN
HvXTH24   FGDGNLVRARDDRAARLLLDRRSGSGFISSDYYLHGFFSASIKLPRDYTAGVVVAFYLSN
HvXTH25   FGGDNLVRSADGRSVTLKLDRYTGSGFVSKSAYRHGFFGASIKLPGDYTAGVVVAFYLSN
HvXTH26   FGDSNLRLHGDGKRVHISLDERTGSGFASQGAYFHGFFSASIKLPSDYAAGVVVAFYVSN
HvXTH27   FGDSNLRLHGDGKRVHISLDERTGSGFASQGAYFHGFFSASIKLPSDYAAGVVVAFYMSN
HvXTH28   FGSGNLALRREGKRVHLALDESTGSGFASQDRFLHGFFSAAVKLPADYAAGVVVAFYLSN
HvXTH29   WGADGYHLVDQGTEIRLTMDRNSGAGFHSKSTYGSGFFHMRIKVPGGYTAGVVTAFYLAS

2UWA      β6 β7 β8
          110       120       130       140       150       160
2UWA      NQDYPGK.HDEIDIEFLGTIPGKPYTLQTNVFIEGSGDYN.IIGREMRIHLWFDPT.QDY
HvXTH22   NEVYPGY.HDEIDVELLGTVPGEPYTLQTNVYVRGTGDAHPIVGREMRFHLWFDPA.AAF
HvXTH23   NEAHPGF.HDEVDMEFLGTIPGEPYTLQTNVYVRGSGDGR.IIGREMRFHLWFDPT.AGF
HvXTH24   GDVYEKT.HDELDFEFLGSRWGGQWRVQTNVY....GNGSTSRGREERYLLPFDPT.LAA
HvXTH25   WDEYPKN.HDELDFELLGNRRGHGWRVQTNMY....GNGSTARGREERYHLPVEPTVTGV
HvXTH26   GDVYEKT.HDELDFEFLGNVRGKEWRVQTNVY....GDGSTAVGREERYGLWFDPT.HDF
HvXTH27   GDVYEKT.HDELDFEFLGNVRGKEWRVQTNVY....GDGSTAVGREER............
HvXTH28   ADVYEKT.HDELDFEFLGNVRGREWRVQTNVY....GNGSTGAGREERYDLPFDPT.DDF
HvXTH29   ETPYDGSDRDEVDFEFLGNVDGENITLQTNVFVNGDGD......REQRLSLWFDPA.ADF

2UWA      β9 β10 β11 η3 β12 η4
          170       180       190       200       210       220
2UWA      HNYAIYWTPSEIIFFVDDVPIR..RYPRK..SDATFPLRPLWVYGSVWDASSWATENGKY
HvXTH22   HHYAVLWNPDEIVFLVDDVPVR..RYQKK..VEATFPEREMWAYGSVWDASDWATDGGRY
HvXTH23   HNYAILWNPDAITFFVDDVPIR..RYERK..TELTFPDRPMWAYGSIWDASDWATDHGRH
HvXTH24   HRYSILWAPTHIIFYVDDTAIR..EVVRHPGMGGDFPAKPMAAYATIWDGSAWATEGGKY
HvXTH25   HRYAIAWTPNNIVFYLDGVPIR..EVVRVPSMGGDFPSKPMSVYATIWDGSAWATDGGKY
HvXTH26   HRYAILWTNRTIVFYVDGTPIR..EVVRSEAMGAQFPSKPMSLYATIWDGSSWATSGGRY
HvXTH27   ..............FYVDGTPIR..EVVRSEAMGAQFPSKPMSLYATIWDGSSWATSGGRY
HvXTH28   HHYSILWTQHRIIFYVDETPIR..EVVRTEAMGAAFPSKPMSLYATIWDGSAWATLGGRY
HvXTH29   HEYKILWNPYHLVILVDDVPIRVLRNLTGQVAEYEFPAKRMAVRASLWDGSDWATDGGRT

2UWA      η5 β13 α2
          230       240       250       260
2UWA      KADYRYQPFVGKYEDFKLGSCTVEA...AS.......SCNPASV.SP.YGQLSQQQVAAM
HvXTH22   RSDYRYQPFVSGFKDFKVAGCEVGA...PA.......SCRPVPA.GP.GGGLSAQQSAAM
HvXTH23   RADYRYQPFVARFDRFVVAGCGPGA...PP.......SCRPVRA.SPVGTGLTRQQYAAM
HvXTH24   KVNYKYAPFASDFSDLSLRGCRVADPASPALRLAGGDGCDLLGLMTADYAVMTPQKRAAM
HvXTH25   KVDYAYAPFAAEFSDLVLSGCGAGNVADPE.......GCQVDLL.THDVAVMAPAKRAAM
HvXTH26   KVEYKYAPYVAEFTDLELRGCASHDRAQPA.......SCEP........EGMPARQRAAM
HvXTH27   KVEYKYAPYVAEFTDLELRGCASHDRAQPA.......SCEP........EGMPARQRAAM
HvXTH28   RANYKYAPFVAEFGDLVLHACPVNRIYHSA.....AAACGTPWY.EPVAAALSGEQRASM
HvXTH29   KIDWGRAPFTAGFRGFDVDACDNAS.STPC.......DSTDLWWNARRHRRLSVREQAAY
                              1             1

2UWA      β14 η6 η7
          270       280       290
2UWA      EWVQKNYMVYNYCDDPTRDHT.LTP.EC.............................
HvXTH22   SWAQQRAMVYYYCQDGSKDRS.NYP.EC.............................
HvXTH23   RWAQQRHMVYYYCQDFRRDRS.LTP.EC.............................
HvXTH24   RAFRARRMTYTVCYDAARYAAGPFP.ECDNSDEERGTFWAWGESKTVVMKTRGRGRRGRG
HvXTH25   RGFREQHLTYTACRDRVRYKTTVFP.ECDDLADGDSSFHLWGESKK.........RRRRS
HvXTH26   ERVRARHMTYGYCYDRARYPA.PLP.ECRVGAEAA....MYLPSGE....ARSSDRR.RH
HvXTH27   ERVRARHMTYGYCYDRARYPA.PLP.ECRVGAEAA....MYLPSGE....ARSSDRR.RH
HvXTH28   SAFRRGHMSYSYCHDRRRYPV.ALS.ECDVAVLPR....LFGPDGM....KYGGDRRHRR
HvXTH29   ENVRRTYMNYDYCADKDRFQNGKLPVECSYTT.............................
                       2              2
```

2UWA

**2UWA**	....
**HvXTH22**	....
**HvXTH23**	....
**HvXTH24**	SRAGAGARGRAGAASS
**HvXTH25**	S.....SPLQYSSSMQ
**HvXTH26**	G.....KRHRRADSAL
**HvXTH27**	G.....KRHRRADSAL
**HvXTH28**	G.....GRGRRSDVVM
**HvXTH29**	....
