# Supplementary material for: Genome-Wide Identification, Characterization and Expression Analysis of Xyloglucan Endotransglucosylase/Hydrolase Genes Family in Barley (Hordeum vulgare)
Source: Molecules. 2019 May 20;24(10):1935. doi: 10.3390/molecules24101935 (PMC6572274; doi:10.3390/molecules24101935)
Supplement: Supplementary file 1 [file molecules-24-01935-s001.zip › Supplementary File 6 The multiple alignment of deduced amino acid sequences of HvXTHs and 2UWA 1-21.pdf]

```
1UN1

1UN1     ............................................................
HvXTH21  ............................................................
HvXTH6   ............................................................
HvXTH7   ............................................................
HvXTH1   MKAPSGLGLAYKKAVSCALCFAPDQSISTLLHSPPLCLPLCGFAVSRPTFVVGGTVFVSS
HvXTH12  ............................................................
HvXTH16  ............................................................
HvXTH5   .........................................................MAP
HvXTH8   ............................................................
HvXTH11  ............................................................
HvXTH3   ............................................................
HvXTH10  ............................................................
HvXTH9   ............................................................
HvXTH15  ............................................................
HvXTH20  ............................................................
HvXTH19  ............................................................
HvXTH18  ............................................................
HvXTH4   .........................................................MAP
HvXTH2   ............................................................
HvXTH14  ..................MRDSSVDMHGWRAQLDGLNTIKRAHNARTVPTRSCYNIALER
HvXTH13  ............................................................
HvXTH17  ...........MAGASAYQTNHTGAEPTAVSRPVPLPLHLPPHFLLLPKKQSRAEQSRAE
HvXTH29  ............................................................
```

```
                                                   α1   β1        η1
1UN1
           1        10             20            30       40
1UN1     ..MAAAYPWTLFLGMLVMVSG.....TMGAA...LRKPVDVAFGRNYVPTWA.....FDH
HvXTH21  .MLRGSLRWLLVLAVVVAASA.....GKAGR..........GLHRDFDAVWG.....KRN
HvXTH6   .......MARMAVSVLAILLA.....WCALA........AASFDKEFDITWG.....DGR
HvXTH7   .......MARMAVSVLSILLA.....TCALA........AASFDKEFDVTWG.....DGR
HvXTH1   WGWGAMGPWRRPCVGALLACAAIAASCCCFQLQGADAAASPSFGDNFEITGA.....KDH
HvXTH12  ...MGKPGALVPVVALAFALV.....LGLEL......VSGGNFYEECDATWE.....PQN
HvXTH16  ...MAPRSDLLAALALALLAA.....SVLST......GAKADFDDQFEVIGD.....RDH
HvXTH5   SLPSSSSCWHSALLVAMLVLV.....VVMDQ......VAMAYLDDDIEVVWG.....DDH
HvXTH8   .......MARMGASVLVILLA.....SCALA........AASFDKEFDVTWG.....DGR
HvXTH11  ..MASGPSRTVPCSVLPLLLL.....LAGVA......RAAGNFYQDVDITWG.....DGR
HvXTH3   ...MGQARAYLLASLAAFYLV.....ALAIP......QVTADMTDEVNLLWG.....NCK
HvXTH10  .......MRTVELGIVAMACL.....VAVAR........AGNFFQDSEMSWG.....DGR
HvXTH9   .......MARMGASVLSILLA.....SCALA........AASFDKEFDVTWG.....DGR
HvXTH15  ...MARPSFSLHLCLAVLALA.....AAASE.........AGFYDQFDVVGS.....GNN
HvXTH20  .....MAQRFLAVLAVALALS.....QVASA........KSWLDKRFNTDGT.....VRT
HvXTH19  .....MARRLLAVLAVALALL.....QAASA........KSWLDKRFNTDGT.....VRT
HvXTH18  .........................................................MSN
HvXTH4   ALPCSRPKLLLLCVALAFLLA.....VDVGR.........ADIYKDIQIIWS.....ADH
HvXTH2   ..MKATAGALLAVVATVLLRG.....IAAAP...PRKPVDVPFEKNYVPTWA.....EDH
HvXTH14  VENMSRPLLVVMAAAAIVVVA.....CCFAACPVGAGASAGGFYENFEVKWGTDPDPDRR
HvXTH13  ..MGMSMRALVAVVAFAAVLK.....LGLVG.........ANFADLCDITWE.....PQN
HvXTH17  QGVMAMAPRAVSLSVAVVVAL.....LATAD.........SWLYEEFTTDGN.....VRA
HvXTH29  MASSVRQPWLLLLLVLLPVMA.................TAAVFDDNYAPTWG.....ADG
```

```
           β2      η2     β3                              β4
1UN1
                 50         60                               70
1UN1     IKYF....NGG..NEIQLHLDK.Y.........................TGTGFQSKGSY
HvXTH21  ARFF....DEG..RVVELALDR.E.........................TGSRLQSKDRY
HvXTH6   GKIL....NNG..QLLTLALDK.V.........................SGSGFQSKHEY
HvXTH7   GKIL....NNG..QLLTLGLDK.V.........................SGSGFQSKHEY
HvXTH1   VKTS....PDG..QTWYLSLDN.K.........................TGVGFQTKQKY
HvXTH12  CWTY....DGG..NSLSLALVSNSSGLSSTSQLLYFSWSSPLNESMCGCSGSMIRSKRQF
HvXTH16  IGYRDDGNDKG..QEFSLELDQ.E.........................SGSGFKSKAKY
HvXTH5   SFFYMD..DAGDDEILALCLDE.T.........................HGSGFHTKEAY
HvXTH8   GKIL....NNG..QLLMLGLDK.V.........................SGSGFQSKREY
HvXTH11  GKIL....GGG..DLLTLSLDR.A.........................SGSGFQSKNQY
HvXTH3   VQRDG...TGR..QTVAMSLDR.W.........................TTSGFSSKIKY
HvXTH10  GKVV....DGG..RGLDLTLDK.T.........................SGSGFQSKSEY
HvXTH9   GKIL....NNG..QLLTLGLDK.V.........................SGSGFQSKHEY
HvXTH15  VRVN....DDGIAQQVALTLDQGN.........................GGSGFSSKDKY
HvXTH20  GYDA....SGQ..QVVMLNLNQQS.........................GAAGFNSKQQY
HvXTH19  GYDA....SGQ..QVVMLNLNQQS.........................GAAGFNSKQQY
HvXTH18  TSTL....SSG......................................DGDG.......
HvXTH4   TYYFMD..GDS..EALALSLDF.N.........................RGSAFKSNDMY
HvXTH2   IHYV....NGG..REVQLSLDK.T.........................TGTGFQTRGSY
HvXTH14  VEIV....DGG..RLVTLTLNN.V.........................SGSGFQSRDAF
HvXTH13  AAMT....DGG..EHLTLSLVSNI.........................SGSMLRTKKTF
HvXTH17  DYNA....QGQ..QVASLILTQQS.........................GGGAFGSRRKY
HvXTH29  YHLV....DQG..TEIRLTMDR.N.........................SGAGFHSKSTY
```

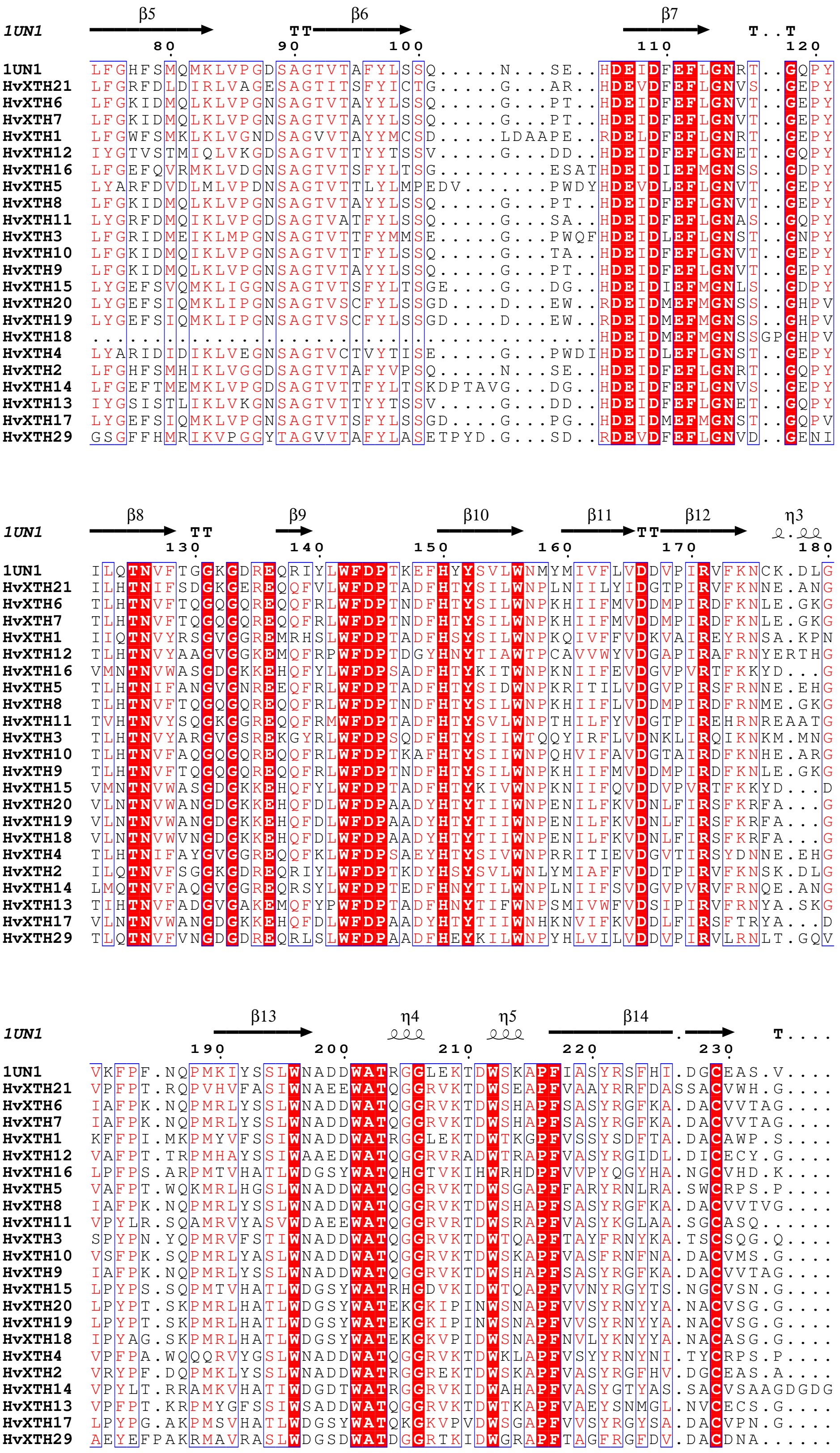

1UN1 β5 TT β6 β7 T..T
80 90 100 110 120
1UN1 LFGHFSMQMKLVPGDSAGTVTAFYLSSQ.....N...SE..HDEIDFEFLGNRT..GQPY
HvXTH21 LFGRFDLDIRLVAGESAGTITSFYICTG.....G...AR..HDEVDFEFLGNVS..GEPY
HvXTH6 LFGKIDMQLKLVPGNSAGTVTAYYLSSQ.....G...PT..HDEIDFEFLGNVT..GEPY
HvXTH7 LFGKIDMQLKLVPGNSAGTVTAYYLSSQ.....G...PT..HDEIDFEFLGNVT..GEPY
HvXTH1 LFGWFSMKLKLVGNDSAGVVTAYYMCSD.....LDAAPE..RDELDFEFLGNRT..GEPY
HvXTH12 IYGTVSTMIQLVKGDSAGTVTTYYTSSV.....G...DD..HDEIDFEFLGNET..GQPY
HvXTH16 LFGEFQVRMKLVDGNSAGTVTSFYLTSG.........ESATHDEIDIEFMGNSS..GDPY
HvXTH5 LYARFDVDLMLVPDNSAGTVTTLYLMPEDV......PWDYHDEVDLEFLGNVT..GEPY
HvXTH8 LFGKIDMQLKLVPGNSAGTVTAYYLSSQ.....G...PT..HDEIDFEFLGNVT..GEPY
HvXTH11 LYGRFDMQIKLVPGDSAGTVATFYLSSQ.....G...SA..HDEIDFEFLGNAS..GQPY
HvXTH3 LFGRIDMEIKLMPGNSAGTVTTFYMMSE.....G...PWQFHDEIDLEFLGNST..GNPY
HvXTH10 LFGKIDMQIKLVPGNSAGTVTTFYLSSQ.....G...TA..HDEIDFEFLGNVT..GEPY
HvXTH9 LFGKIDMQLKLVPGNSAGTVTAYYLSSQ.....G...PT..HDEIDFEFLGNVT..GEPY
HvXTH15 LYGEFSVQMKLIGGNSAGTVTSFYLTSGE....G...DG..HDEIDIEFMGNLS..GDPY
HvXTH20 LYGEFSIQMKLIPGNSAGTVSCFYLSSGD....D...EW..RDEIDMEFMGNSS..GHPV
HvXTH19 LYGEFSIQMKLIPGNSAGTVSCFYLSSGD....D...EW..RDEIDMEFMGNSS..GHPV
HvXTH18 ....HDEIDMEFMGNSSGPGHPV
HvXTH4 LYARIDIDIKLVEGNSAGTVCTVYTISE.....G...PWDIHDEIDLEFLGNST..GEPY
HvXTH2 LFGHFSMHIKLVGGDSAGTVTAFYVPSQ.....N...SE..HDEIDFEFLGNRT..GQPY
HvXTH14 LFGEFTMEMKLVPGDSAGTVTTFYLTSKDPTAVG...DG..HDEIDFEFLGNVS..GEPY
HvXTH13 IYGSISTLIKLVKGNSAGTVTTYYTSSV.....G...DD..HDEIDFEFLGNET..GQPY
HvXTH17 LYGEFSIQMKLVPGNSAGTVTSFYLSSGD....G...PG..HDEIDMEFMGNST..GQPV
HvXTH29 GSGFFHMRIKVPGGYTAGVVTAFYLASETPYD.G...SD..RDEVDFEFLGNVD..GENI
1UN1 β8 TT β9 β10 β11 TT β12 η3
130 140 150 160 170 180
1UN1 ILQTNVFTGGKGDREQRIYLWFDPTKEFHYYSVLWNMYMIVFLVDDVPIRVFKNCK.DLG
HvXTH21 ILHTNIFSDGKGEREQQFVLWFDPTADFHTYSILWNPLNIILYIDGTPIRVFKNNE.ANG
HvXTH6 TLHTNVFTQGQGQREQQFRLWFDPTNDFHTYSILWNPKHIIFMVDDMPIRDFKNLE.GKG
HvXTH7 TLHTNVFTQGQGQREQQFRLWFDPTNDFHTYSILWNPKHIIFMVDDMPIRDFKNLE.GKG
HvXTH1 IIQTNVYRSGVGGREMRHSLWFDPTADFHSYSILWNPKQIVFFVDKVAIREYRNSA.KPN
HvXTH12 TLHTNVYAAGVGGKEMQFRPWFDPTDGYHNYTIAWTPCAVVWYVDGAPIRAFRNYERTHG
HvXTH16 VMNTNVWASGDGKKEHQFYLWFDPSADFHTYKITWNPKNIIFEVDGVPVRTFKKYD...G
HvXTH5 TLHTNIFANGVGNREEQFRLWFDPTADFHTYSIDWNPKRITILVDGVPIRSFRNNE.EHG
HvXTH8 TLHTNVFTQGQGQREQQFRLWFDPTNDFHTYSILWNPKHIIFLVDDMPIRDFRNME.GKG
HvXTH11 TVHTNVYSQGKGGREQQFRMWFDPTADFHTYSVLWNPTHILFYVDGTPIREHRNREAATG
HvXTH3 TLHTNVYARGVGSREKGYRLWFDPSQDFHTYSIIWTQQYIRFLVDNKLIRQIKNKM.MNG
HvXTH10 TLHTNVFAQGQGQREQQFRLWFDPTKAFHTYSIIWNPQHVIFAVDGTAIRDFKNHE.ARG
HvXTH9 TLHTNVFTQGQGQREQQFRLWFDPTNDFHTYSILWNPKHIIFMVDDMPIRDFKNLE.GKG
HvXTH15 VMNTNVWASGDGKKEHQFYLWFDPTADFHTYKIVWNPKNIIFQVDDVPVRTFKKYD...D
HvXTH20 VLNTNVWANGDGKKEHQFDLWFDPAADYHTYTIIWNPENILFKVDNLFIRSFKRFA...G
HvXTH19 VLNTNVWANGDGKKEHQFDLWFDPAADYHTYTIIWNPENILFKVDNLFIRSFKRFA...G
HvXTH18 VLNTNVWVNGDGKKEHQFDLWFDPAADYHTYTIIWNPENILFKVDNLFIRSFKRFA...G
HvXTH4 TLHTNIFAYGVGGREQQFKLWFDPSAEYHTYSIVWNPRRITIEVDGVTIRSYDNNE.EHG
HvXTH2 ILQTNVFSGGKGDREQRIYLWFDPTKDYHSYSVLWNLYMIAFFVDDTPIRVFKNSK.DLG
HvXTH14 LMQTNVFAQGVGGREQRSYLWFDPTEDFHNYTILWNPLNIIFSVDGVPVRVFRNQE.ANG
HvXTH13 TIHTNVFADGVGAKEMQFYPWFDPTADFHNYTIFWNPSMIVWFVDSIPIRVFRNYA.SKG
HvXTH17 VLNTNVWANGDGKKEHQFDLWFDPAADYHTYTIIWNHKNVIFKVDDLFIRSFTRYA...D
HvXTH29 TLQTNVFVNGDGDREQRLSLWFDPAADFHEYKILWNPYHLVILVDDVPIRVLRNLT.GQV
1UN1 β13 η4 η5 β14 T....
190 200 210 220 230
1UN1 VKFPF.NQPMKIYSSLWNADDWATRGGLEKTDWSKAPFIASYRSFHI.DGCEAS.V....
HvXTH21 VPFPT.RQPVHVFASIWNAEEWATQGGRVKTDWSEAPFVAAYRRFDASSACVWH.G....
HvXTH6 IAFPK.NQPMRLYSSLWNADDWATQGGRVKTDWSHAPFSASYRGFKA.DACVVTAG....
HvXTH7 IAFPK.NQPMRLYSSLWNADDWATQGGRVKTDWSHAPFSASYRGFKA.DACVVTAG....
HvXTH1 KFFPI.MKPMYVFSSIWNADDWATRGGLEKTDWTKGPFVSSYSDFTA.DACAWP.S....
HvXTH12 VAFPT.TRPMHAYSSIWAAEDWATQGGRVRADWTRAPFVASYRGIDL.DICECY.G....
HvXTH16 LPFPS.ARPMTVHATLWDGSYWATQHGTVKIHWRHDPFVVPYQGYHA.NGCVHD.K....
HvXTH5 VAFPT.WQKMRLHGSLWNADDWATQGGRVKTDWSGAPFFARYRNLRA.SWCRPS.P....
HvXTH8 IAFPK.NQPMRLYSSLWNADDWATQGGRVKTDWSHAPFSASYRGFKA.DACVVTVG....
HvXTH11 VPYLR.SQAMRVYASVWDAEEWATQGGRVRTDWSRAPFVASYKGLAA.SGCASQ......
HvXTH3 SPYPN.YQPMRVFSTIWNADDWATQGGRVKTDWTQAPFTAYFRNYKA.TSCSQG.Q....
HvXTH10 VSFPK.SQPMRLYASLWNADDWATQGGRVKTDWSKAPFVASFRNFNA.DACVMS.G....
HvXTH9 IAFPK.NQPMRLYSSLWNADDWATQGGRVKTDWSHAPFSASYRGFKA.DACVVTAG....
HvXTH15 LPYPS.SQPMTVHATLWDGSYWATRHGDVKIDWTQAPFVVNYRGYTS.NGCVSN.G....
HvXTH20 LPYPT.SKPMRLHATLWDGSYWATEKGKIPINWSNAPFVVSYRNYYA.NACVSG.G....
HvXTH19 LPYPT.SKPMRLHATLWDGSYWATEKGKIPINWSNAPFVVSYRNYYA.NACVSG.G....
HvXTH18 IPYAG.SKPMRLHATLWDGSYWATEKGKVPIDWSNAPFNVLYKNYYA.NACASG.G....
HvXTH4 VPFPA.WQQRVYGSLWNADDWATQGGRVKTDWKLAPFVSYYRNYNI.TYCRPS.P....
HvXTH2 VRYPF.DQPMKLYSSLWNADDWATRGGREKTDWSKAPFVASYRGFHV.DGCEAS.A....
HvXTH14 VPYLT.RRAMKVHATIWDGDTWATRGGRVKIDWAHAPFVASYGTYAS.SACVSAAGDGDG
HvXTH13 VPFPT.KRPMYGFSSIWSADDWATQGGRVKTDWTKAPFVAEYSNMGL.NVCECS.G....
HvXTH17 LPYPG.AKPMSVHATLWDGSYWATQKGKVPVDWSGAPFVVSYRGYSA.DACVPN.G....
HvXTH29 AEYEFPAKRMAVRASLWDGSDWATDGGRTKIDWGRAPFTAGFRGFDV.DACDNA......

```
                                                                η6   η7          α2              β15
1UN1      ..TT
                   240                                           250            260             270
1UN1      ..EAK..FCATQG..............ARWWDQKEFQDLDAFQYRRLSWVRQK.YTIYNY
HvXTH21   ..GASPTRCGGDHLPSSA.........SSWMG....QRLDWWSWMTLNWVRMN.YMTYDY
HvXTH6    ..GRP..HCGASVGTDVAPGTGAA...GEWYN....QELDLTRQQRMRWVQSN.YMIYNY
HvXTH7    ..GRP..RCGASVGTDVAPGTGAA...GEWYN....QELDLTRQQRMRWVQSN.YMIYNY
HvXTH1    ..GPAPPACAAATG.............DSWWDQPPAWALDDGQRRDSGWVARN.LVIYDY
HvXTH12   ..GDCVYTCA.................GAFRG...CGGLTGDQRGKMQWVQDN.YRIYDY
HvXTH16   ..ATNKTSCPAGS..............DAWMH....RELDDGELSTVAWAERN.CLSYNY
HvXTH5    ..GVA..WCGDEPPG............STWFE....RGLDAAALRRARDA....HMIYDY
HvXTH8    ..GRP..RCGASIGTDAAPGTGGAAAVGDWYN....QELDLTRQQRMRWVQSN.YMIYNY
HvXTH11   ..DAA..ACANSN..............GAWMY....QELDATALDRLQWVQKN.YMIYNY
HvXTH3    ..NSN..VCGQSSP.............NGLFN....QQQDQMQQQQVKEVDAK.YKVYDF
HvXTH10   ..GAQ..RCPAGTMEASAAGG......GSWWN....QELSGMGYRRMRWVQRK.FMIYNY
HvXTH9    ..GRP..RCGASMGTEAAPGTGASGAAGEWYN....QELDLTLQQRMRWVQSN.YMIYNY
HvXTH15   ..GSS..ACPAGS..............DAWMS....TELDAKALGTVAWAESK.YMSYDY
HvXTH20   ......ACHAGS...............DRWMR....KQLDGDEWGTVKWAERS.YMRYNY
HvXTH19   ......ACHAGS...............DRWMK....KQLDGAEWGTVKWAERS.YMRYNY
HvXTH18   ......ACHAGS...............DGWMN....RQLDGSEWGTVKWAEQN.YMRYNY
HvXTH4    ..GVS..WCGAEPAG............SPVFN......LAPKARADMQWVRDMGYVIYDY
HvXTH2    ..EAK..LCATQG..............ARWWDQPEFQDLDAAQYRRLAWVRKE.HTIYNY
HvXTH14   DEDVPSAFCCPGDA.............ASWMA....RRLGPDGERAVAWARDK.YMVMDY
HvXTH13   ..ADA..ECAAGCNA............STAPE...PSQLTKEQMRQLRAVQLG.YTIYDY
HvXTH17   ..DGRPLSCPAGT..............GRWMD....RQLDDAERGTVAWARRD.YMRYNY
HvXTH29   ..SST..PCDST...............DLWWNARRHRRLSVREQAAYENVRRT.YMNYDY

          η8                 α3
1UN1
              280          290
1UN1      CTD.RSRYP..SMPP.ECKRDRDI...
HvXTH21   CAD.RKRYPH.GFPA.ECIIPIGRI..
HvXTH6    CTD.PKRFAQ.GVPA.ECSM.......
HvXTH7    CTD.PKRFAQ.GVPA.ECSM.......
HvXTH1    CGD.RKRFP..TVPE.ECALRTTTS..
HvXTH12   CAD....HEAGKVPGVECSLPQY....
HvXTH16   CAD.GWRFPK.GFPG.ECGRK......
HvXTH5    CKD.LQRYKGSGLPK.ECVVD......
HvXTH8    CTD.PKRVAK.GVPA.ECSM.......
HvXTH11   CTD.TWRFKD.GAPP.ECASK......
HvXTH3    CDDSKRRI...GSSE.DCQSQ......
HvXTH10   CTD.PKRVAQ.GVPA.ECKLR......
HvXTH9    CTD.PKRVAK.GVPA.ECSM.......
HvXTH15   CTD.GWRFPN.GFPA.ECSRRN.....
HvXTH20   CED.GYRFPQ.GLPA.ECNRY......
HvXTH19   CED.GYRFPQ.GLPA.ECNRY......
HvXTH18   CAD.GYRFPQ.GFPA.ECSRY......
HvXTH4    CTDRSNRYNDTTRPK.ECSLPPRP...
HvXTH2    CTD.RERYA..AMSP.ECKRDRDV...
HvXTH14   CDD.PWNL...GRPA.ECDMDRLASAV
HvXTH13   CAS.AREDGKGPVPP.ECDMEQY....
HvXTH17   CDD.GWRFPQ.GFPA.ECSRG......
HvXTH29   CAD.KDRFQNGKLPV.ECSYTT.....
          1                1
```
