# Supplementary material for: Genome-Wide Identification, Characterization and Expression Analysis of Xyloglucan Endotransglucosylase/Hydrolase Genes Family in Barley (Hordeum vulgare)
Source: Molecules. 2019 May 20;24(10):1935. doi: 10.3390/molecules24101935 (PMC6572274; doi:10.3390/molecules24101935)
Supplement: Supplementary file 1 [file molecules-24-01935-s001.zip › Supplementary File 7 The multiple alignment of deduced amino acid sequences of HvXTHs and EG16.pdf]

```
                         1                                                   10
HvEG16_Hordeum_vul     ..MASER............................................DQHEHLR..
TaEG16_Triticum_ae     ..MASDSESS...................................DVA.PDDQHEHLR..
TaEG16_Triticum_ae     ..MASDSESS...................................DVA.PDDQHEHLR..
OsEG16_Oryza_sativ     ..MASESECV...................................AVAEPPHVHVHLH..
OsEG16_Oryza_sativ     ..MASESECV...................................AVAEPPHVHVHLH..
OsEG16_Oryza_sativ     ..MASESECV...................................AVAEPPHVHVHLH..
TaEG16_Triticum_ae     ..MASECE......................................VT.PDDQHEHLR..
ZmEG16_Zea_mays_GR     ............................................................
ZmEG16_Zea_mays_NP     ..MASESEAC.SWE...................................PHLLH......
ZmEG16_Zea_mays_Zm     ..MASESEAC.SWE...................................PHLLH......
VvEG16                 ..MAD............................................PSLHHEA....
PtEG16                 ..MAD............................................PAIHHET....
1UN1                   ..MAAAYPWT..................................................
HvXTH8                 ..MKATAGAL..................................................
HvXTH22                ..M.........................................................
HvXTH20                ..MA........................................................
HvXTH19                ..MA........................................................
HvXTH18                ..MA........................................................
HvXTH17                ..MA........................................................
HvXTH23                ..MRTVE.....................................................
HvXTH21                ..MASG...........................................PS.........
HvXTH24                ..MG........................................................
HvXTH3                 ..MKAPSGLGLAYKKAVSCALCFAPDQSISTLLHSPPLCLPLCGFAVSRPTFVVGGTVFV
HvXTH13                ..MAPSL..........................................PS.........
HvXTH4                 ..MAPAL..........................................P..........
HvXTH14                ..MA........................................................
HvXTH1                 ..MA........................................................
HvXTH6                 ..MA........................................................
HvXTH5                 ..MA........................................................
HvXTH7                 ..MS........................................................
HvXTH2                 ..MG........................................................
HvXTH15                ..MA........................................................
2UWA                   .....M..........................................PPN.........
HvXTH10                ..MAMMQ.......................................IRRPH.........
HvXTH16                ..MASL......................................................
HvXTH12                MEMTARF.....................................................
HvXTH9                 ..MACHF.....................................................
HvXTH11                ..MASSSSCP.........................PP.........SPRPS.........
HORVU4Hr1G085940.2     ..MGS.......................................................
```

```
HvEG16_Hordeum_vul     .............................................PDGTEPL........
TaEG16_Triticum_ae     .............................................PEGTEPL........
TaEG16_Triticum_ae     .............................................PEGTEPL........
OsEG16_Oryza_sativ     .............................................PDGTEPL........
OsEG16_Oryza_sativ     .............................................PDGTEPL........
OsEG16_Oryza_sativ     .............................................PDGTEPL........
TaEG16_Triticum_ae     .............................................PEGTEPL........
ZmEG16_Zea_mays_GR     ............................................................
ZmEG16_Zea_mays_NP     .............................................PNGTEPL........
ZmEG16_Zea_mays_Zm     .............................................PNGTEPL........
VvEG16                 .................................................QPL........
PtEG16                 .................................................QPI........
1UN1                   ....................LFLGMLVMVS....GTMGAALR.......KPV........
HvXTH8                 ....................LAVVATVLLR....GIAAAPPR.......KPV........
HvXTH22                ...........LRGSLRWL.LVLAVVVAAS......AGKAGR..................
HvXTH20                ..........RMAVSVLAI.LLAWCALA........AAS.....................
HvXTH19                ..........RMAVSVLSI.LLATCALA........AAS.....................
HvXTH18                ..........RMGASVLSI.LLASCALA........AAS.....................
HvXTH17                ..........RMGASVLVI.LLASCALA........AAS.....................
HvXTH23                ................LGI.VAMACLVAVAR.....AGN.....................
HvXTH21                .........RTVPCSVLPL.LLLLAGVARA......AGN.....................
HvXTH24                ..........QARAYLLAS.LAAFYLVALAI..PQVTAD.....................
HvXTH3                 SSWGWGAMGPWRRPCVGAL.LACAAIAASCCCFQLQGADAAA.......SPS........
HvXTH13                .......SSSCWHSALL...VAMLVLVVVM....DQVAMAY...................
HvXTH4                 ..........CSRPKLLLLCVALAFLLAVD.....VGRAD....................
HvXTH14                ...........PRSDLLAA.LALALLAASVL...STGAKAD...................
HvXTH1                 ..........RPSFSLHLC.LAVLALAAA.......ASEAG...................
HvXTH6                 ...........QRFLAVLA.VALALSQVA.......SAKSW...................
HvXTH5                 ...........RRLLAVLA.VALALLQAA.......SAKSW...................
HvXTH7                 ............................................................
HvXTH2                 ...........KPGALVPV.VALAFALVLGL...ELVSGGN...................
HvXTH15                ........SSVRQPWLLLL.LVLLPVMATAAV............................
2UWA                   ...........ILSIFLHL.LPILMFSSS.CL..GQGP.......P...SPGYYPSSQIT
HvXTH10                .............DAISHL.MVIVVGAVI.LL.QGEAQ.......P...SPGYYPSSKVS
HvXTH16                ..............SLLPA.MALLLLAMA.VA.SSDAQ.......P...SPGYYPSSRFR
HvXTH12                .......................LAAAAACVW.LAAAASAFDV.......PT........
HvXTH9                 ..................L.LAVLLASSSWVA.ASSGAAADDVMVP...RPT.....TAA
HvXTH11                .................RL.LPVLVATV..VL.LGRGGEARQ...P...APLH...GVVR
HORVU4Hr1G085940.2     ...........LEATRVELGLAVLYLNKAE..............................
```

```
                     20        30        40
HvEG16_Hordeum_vul   .....ARIAVDYTPDACRHAPE..SG..EIHVTYDD........................
TaEG16_Triticum_ae   .....ARIAVDYTPDACRHAPE..TG..EIHVTYDH........................
TaEG16_Triticum_ae   .....ASIAVDYIPDACRHAPA..SG..EIHVTYDQ........................
OsEG16_Oryza_sativ   .....AHIAVDYCPEACHHASE..DG..EIHVTYDD........................
OsEG16_Oryza_sativ   .....AHIAVDYCPEACHHASE..DG..EIHVTYDD........................
OsEG16_Oryza_sativ   .....AHIAVDYCPEACHHASE..DG..EIHVTYDD........................
TaEG16_Triticum_ae   .....ARIAVDYTPD..........G..EIHVTYDH........................
ZmEG16_Zea_mays_GR   ............................................................
ZmEG16_Zea_mays_NP   .....HRIAVDYCPEACLHERH..AG..EIHVTYDD........................
ZmEG16_Zea_mays_Zm   .....HRIAVDYCPEACLHERH..AG..EIHVTYDD........................
VvEG16               .....KFIAVDYCPESCTHSPE..SS..TITLTFDH........................
PtEG16               .....NQIAIDYTPEACTHCPE..SN..SITLTYDH........................
1UN1                 DVAFGRNYVPTWAFDHIKYFNG..GN..EIQLHLDK........................
HvXTH8               DVPFEKNYVPTWAEDHIHYVNG..GR..EVQLSLDK........................
HvXTH22              ..GLHRDFDAVWGKRNARFFDE..GR..VVELALDR........................
HvXTH20              ...FDKEFDITWGDGRGKILNN..GQ..LLTLALDK........................
HvXTH19              ...FDKEFDVTWGDGRGKILNN..GQ..LLTLGLDK........................
HvXTH18              ...FDKEFDVTWGDGRGKILNN..GQ..LLTLGLDK........................
HvXTH17              ...FDKEFDVTWGDGRGKILNN..GQ..LLMLGLDK........................
HvXTH23              ...FFQDSEMSWGDGRGKVVDG..GR..GLDLTLDK........................
HvXTH21              ...FYQDVDITWGDGRGKILGG..GD..LLTLSLDR........................
HvXTH24              ...MTDEVNLLWGNCKVQRDGT..GR.QTVAMSLDR........................
HvXTH3               ...FGDNFEITGAKDHVKTSPD..GQ..TWYLSLDN........................
HvXTH13              ...LDDDIEVVWGDDHSFFYMDDAGDDEILALCLDE........................
HvXTH4               ...IYKDIQIIWSADHTYYFMD..GDSEALALSLDF........................
HvXTH14              ...FDDQFEVIGDRDHIGYRDDGNDKGQEFSLELDQ........................
HvXTH1               ...FYDQFDVVGSGNNVRVNDDGIAQ.QV.ALTLDQ........................
HvXTH6               ...LDKRFNTDGT...VRTGYDASGQ.QVVMLNLNQ........................
HvXTH5               ...LDKRFNTDGT...VRTGYDASGQ.QVVMLNLNQ........................
HvXTH7               ............................................................
HvXTH2               ...FYEECDATWEPQNCWTYDG..GN..SLSLALVSNSSGLSSTSQLLYFSWSSPLNESM
HvXTH15              ...FDDNYAPTWGADGYHLVDQ..GT..EIRLTMDR........................
2UWA                 SLGFDQGYTNLWGPQHQRV..D..QG..SLTIWLDS........................
HvXTH10              STPFSQWYSTLWGPQHQSLSPD..QT..ALTLWMDR........................
HvXTH16              PVAFNRGYSNKWGPQHQTVSGD..HS..AITIWLDR........................
HvXTH12              .VAFEEGFSPLFGDGNLVRARD..DR..AARLLLDR........................
HvXTH9               ALTFREGYTQLFGDSNLRLHGD..GK..RVHISLDE........................
HvXTH11              SMAFDEGYTQLFGSGNLALRRE..GK..RVHLALDE........................
HORVU4Hr1G085940.2   ........................AR........DKICRAI...................

                     50        60        70        80        90
HvEG16_Hordeum_vul   ..RGGARWRSRSRFLPGGAVAAAVRAPAGDTTGLNYNLYLSSLEGS...GDMDEI.....
TaEG16_Triticum_ae   ..RGGARWRSRSRFLPGGAVAAAVRAPAGDTAGLNYNLYLSSLEGS...GDMDEI.....
TaEG16_Triticum_ae   ..RGGARWRSRSRFLPGGAVAAAVRAPAGDTAGLNYNLYLSSLEGS...GDMDEI.....
OsEG16_Oryza_sativ   ..RGGARWRSRCRFLPGGAVAATIRAPAGDTAGLNYNLYLSSLEGS...RDMDEI.....
OsEG16_Oryza_sativ   ..RGGARWRSRCRFLPGGAVAATIRAPAGDTAGLNYNLYLSSLEGS...RDMDEI.....
OsEG16_Oryza_sativ   ..RGGARWRSRCRFLPGGAVAATIRAPAGDTAGLNYNLYLSSLEGS...RDMDEI.....
TaEG16_Triticum_ae   ....GARWRSRGRFLPGCAVAATVRAPAGDTAGLNYNLYLSSMEGS...SDMDEI.....
ZmEG16_Zea_mays_GR   ............................................................
ZmEG16_Zea_mays_NP   ..RGGARWRSCCRFLPGSAVATTIRAPAGDTSGLNYNIYLSSLEGS...ADQDEI.....
ZmEG16_Zea_mays_Zm   ..RGGARWRSCCRFLPGSAVATTIRAPAGDTSGLNYNIYLSSLEGS...ADQDEI.....
VvEG16               ..RGGSRWRSTTRFQYG.TFSSLIQCPKGNTSGLNFNIYLSSLEGD...KSQDEI.....
PtEG16               ..RGGARWRSTTRFLYG.TFSSLIQCPKGNTSGLNFNIYLSSLEGD...KSQDEI.....
1UN1                 ..YTGTGFQSKGSYLFG.HFSMQMKLVPGDSAGTVTAFYLSSQN.....SEHDEI.....
HvXTH8               ..TTGTGFQTRGSYLFG.HFSMHIKLVGGDSAGTVTAFYVPSQN.....SEHDEI.....
HvXTH22              ..ETGSRLQSKDRYLFG.RFDLDIRLVAGESAGTITSFYICTGG.....ARHDEV.....
HvXTH20              ..VSGSGFQSKHEYLFG.KIDMQLKLVPGNSAGTVTAYYLSSQG.....PTHDEI.....
HvXTH19              ..VSGSGFQSKHEYLFG.KIDMQLKLVPGNSAGTVTAYYLSSQG.....PTHDEI.....
HvXTH18              ..VSGSGFQSKHEYLFG.KIDMQLKLVPGNSAGTVTAYYLSSQG.....PTHDEI.....
HvXTH17              ..VSGSGFQSKREYLFG.KIDMQLKLVPGNSAGTVTAYYLSSQG.....PTHDEI.....
HvXTH23              ..TSGSGFQSKSEYLFG.KIDMQIKLVPGNSAGTVTTFYLSSQG.....TAHDEI.....
HvXTH21              ..ASGSGFQSKNQYLYG.RFDMQIKLVPGDSAGTVATFYLSSQG.....SAHDEI.....
HvXTH24              ..WTTSGFSSKIKYLFG.RIDMEIKLMPGNSAGTVTTFYMMSEG.PW..QFHDEI.....
HvXTH3               ..KTGVGFQTKQKYLFG.WFSMKLKLVGNDSAGVVTAYYMCSDLDAA..PERDEL.....
HvXTH13              ..THGSGFHTKEAYLYA.RFDVDLMLVPDNSAGTVTTLYLMPEDVPW..DYHDEV.....
HvXTH4               ..NRGSAFKSNDMYLYA.RIDIDIKLVEGNSAGTVCTVYTISEG.PW..DIHDEI.....
HvXTH14              ..ESGSGFKSKAKYLFG.EFQVRMKLVDGNSAGTVTSFYLTSGESA....THDEI.....
HvXTH1               .GNGGSGFSSKDKYLYG.EFSVQMKLIGGNSAGTVTSFYLTSGEGD....GHDEI.....
HvXTH6               .QSGAAGFNSKQQYLYG.EFSIQMKLIPGNSAGTVSCFYLSSGDDE....WRDEI.....
HvXTH5               .QSGAAGFNSKQQYLYG.EFSIQMKLIPGNSAGTVSCFYLSSGDDE....WRDEI.....
HvXTH7               ...................................NTSTLSSGDGD....GHDEI.....
HvXTH2               CGCSGSMIRSKRQFIYG.TVSTMIQLVKGDSAGTVTTYYTSSVG.....DDHDEI.....
HvXTH15              ..NSGAGFHSKSTYGSG.FFHMRIKVPGGYTAGVVTAFYLASET.PYDGSDRDEV.....
2UWA                 ..TSGSGFKSINRYRSG.YFGANIKLQSGYTAGVITSFYLSNNQDYP..GKHDEI.....
HvXTH10              ..SSGSGFKSKRSYRNG.YFGVSMKVQPGYTAGVNTAFYLSNNEVYP..GYHDEI.....
HvXTH16              ..TCGSGFKSKHAYRNG.YFATRIKLPAGYTAGTNTAFYLSNNEAHP..GFHDEV.....
HvXTH12              ..RSGSGFISSDYYLHG.FFSASIKLPRDYTAGVVVAFYLSNGDVYE..KTHDEL.....
HvXTH9               ..RTGSGFASQGAYFHG.FFSASIKLPSDYAAGVVVAFYVSNGDVYE..KTHDEL.....
HvXTH11              ..STGSGFASQDRFLHG.FFSAAVKLPADYAAGVVVAFYLSNADVYE..KTHDEL.....
HORVU4Hr1G085940.2   ..QYGSKFISNGQPGTAQNVDKSTTL....ARKVFRLLKVSSLAHSRPHSHHDNLATSPS
```

```
                                       100                                    110
HvEG16_Hordeum_vul      ..............DFEFLGNDKR.....AV........................QTNFF
TaEG16_Triticum_ae      ..............DFEFLGNHKR.....AV........................QTNFF
TaEG16_Triticum_ae      ..............DFEFLGNDKR.....AV........................QTNFF
OsEG16_Oryza_sativ      ..............DFEFLGHDKC.....AV........................QTNFH
OsEG16_Oryza_sativ      ..............DFEFLGHDKC.....AV........................QTNFH
OsEG16_Oryza_sativ      ..............DFEFLGHDKC.....AV........................QTNFH
TaEG16_Triticum_ae      ..............DFEFLGNDKR.....AV........................QTNLF
ZmEG16_Zea_mays_GR      ............................................................
ZmEG16_Zea_mays_NP      ..............DFEFLGHDKR.....AV........................QTNYY
ZmEG16_Zea_mays_Zm      ..............DFEFLGHDKR.....AV........................QTNYY
VvEG16                  ..............DFEFLGKDKR.....IV........................QTNYY
PtEG16                  ..............DFEFLGKDKT.....IV........................QTNYY
1UN1                    ..............DFEFLGNRT..GQPYIL........................QTNVF
HvXTH8                  ..............DFEFLGNRT..GQPYIL........................QTNVF
HvXTH22                 ..............DFEFLGNVS..GEPYIL........................HTNIF
HvXTH20                 ..............DFEFLGNVT..GEPYTL........................HTNVF
HvXTH19                 ..............DFEFLGNVT..GEPYTL........................HTNVF
HvXTH18                 ..............DFEFLGNVT..GEPYTL........................HTNVF
HvXTH17                 ..............DFEFLGNVT..GEPYTL........................HTNVF
HvXTH23                 ..............DFEFLGNVT..GEPYTL........................HTNVF
HvXTH21                 ..............DFEFLGNAS..GQPYTV........................HTNVY
HvXTH24                 ..............DLEFLGNST..GNPYTL........................HTNVY
HvXTH3                  ..............DFEFLGNRT..GEPYII........................QTNVY
HvXTH13                 ..............DLEFLGNVT..GEPYTL........................HTNIF
HvXTH4                  ..............DLEFLGNST..GEPYTL........................HTNIF
HvXTH14                 ..............DIEFMGNSS..GDPYVM........................NTNVW
HvXTH1                  ..............DIEFMGNLS..GDPYVM........................NTNVW
HvXTH6                  ..............DMEFMGNSS..GHPVVL........................NTNVW
HvXTH5                  ..............DMEFMGNSS..GHPVVL........................NTNVW
HvXTH7                  ..............DMEFMGNSSGPGHPVVL........................NTNVW
HvXTH2                  ..............DFEFLGNET..GQPYTL........................HTNVY
HvXTH15                 ..............DFEFLGNVD..GENITL........................QTNVF
2UWA                    ..............DIEFLGTIP..GKPYTL........................QTNVF
HvXTH10                 ..............DVELLGTVP..GEPYTL........................QTNVY
HvXTH16                 ..............DMEFLGTIP..GEPYTL........................QTNVY
HvXTH12                 ..............DFEFLGSRW..GGQWRV........................QTNVY
HvXTH9                  ..............DFEFLGNVR..GKEWRV........................QTNVY
HvXTH11                 ..............DFEFLGNVR..GREWRV........................QTNVY
HORVU4Hr1G085940.2      QCYVNLVLVLQWVNDLHALISPAAKGTPLTLVLLGKSKNALLSTFLFLDQFVWLGRSGIY
```

```
                                  120        130        140            150        160
HvEG16_Hordeum_vul      VAGSG......GREAVHELPFDSSDGFHHYAVAWDAEAIE..WRVDGEVLRREERRD...
TaEG16_Triticum_ae      VAGCG......GREAVHELPFDSSDGFHHYAVAWGAEAIE..WRVDGE...........
TaEG16_Triticum_ae      VAGCG......GREAIHDLPFDSSDGFHHYAVAWGAEAIE..WRVDGEVLRREERRD...
OsEG16_Oryza_sativ      VAGGG......GREQIHVLPFDSSDGFHHYAIAWGADAIE..WRIDGELIRREERVA...
OsEG16_Oryza_sativ      VAGGG......GREQIHVLPFDSSDGFHHYAIAWGADAIE..WRIDGELIRREERVA...
OsEG16_Oryza_sativ      VAGGG......GREQIHVLPFDSSDGFHHYAIAWGADAIE..WRIDGELIRREERVA...
TaEG16_Triticum_ae      VAGGG......GREMVHQLPFDSSDGFHHYAVAWSAEAVE..WRVDGEVIRREERRD...
ZmEG16_Zea_mays_GR      ......................................................MRE...
ZmEG16_Zea_mays_NP      VGGNG......GREQIHALPFDSSDGFHHYAIAWDAAAIE..WRVDGELVRRDKRRE...
ZmEG16_Zea_mays_Zm      VGGNG......GREQIHALPFDSSDGFHHYAIAWDAAAIEWRWRVDGELVRRDKRRE...
VvEG16                  TAGTG......NREAIHDLGFDCSDGFHEYVIKWGPDLIQ..WLIDGKVIRSVVRAD...
PtEG16                  ASGTG......NREEIHDLGFDCSDAFHEYVIKWCPNFIE..WLIDGKVVRKVEKRE...
1UN1                    TGGKG......DREQRIYLWFDPTKEFHYYSVLWNMYMIV..FLVDDVPIRVFKNCK..D
HvXTH8                  SGGKG......DREQRIYLWFDPTKDYHSYSVLWNLYMIA..FFVDDTPIRVFKNSK..D
HvXTH22                 SDGKG......EREQQFVLWFDPTADFHTYSILWNPLNII..LYIDGTPIRVFKNNE..A
HvXTH20                 TQGQG......QREQQFRLWFDPTNDFHTYSILWNPKHII..FMVDDMPIRDFKNLE..G
HvXTH19                 TQGQG......QREQQFRLWFDPTNDFHTYSILWNPKHII..FMVDDMPIRDFKNLE..G
HvXTH18                 TQGQG......QREQQFRLWFDPTNDFHTYSILWNPKHII..FMVDDMPIRDFKNLE..G
HvXTH17                 TQGQG......QREQQFRLWFDPTNDFHTYSILWNPKHII..FLVDDMPIRDFRNME..G
HvXTH23                 AQGQG......QREQQFRLWFDPTKAFHTYSIIWNPQHVI..FAVDGTAIRDFKNHE..A
HvXTH21                 SQGKG......GREQQFRMWFDPTADFHTYSVLWNPTHIL..FYVDGTPIREHRNREA.A
HvXTH24                 ARGVG......SREKGYRLWFDPSQDFHTYSIIWTQQYIR..FLVDNKLIRQIKNKM..M
HvXTH3                  RSGVG......GREMRHSLWFDPTADFHSYSILWNPKQIV..FFVDKVAIREYRNSA..K
HvXTH13                 ANGVG......NREEQFRLWFDPTADFHTYSIDWNPKRIT..ILVDGVPIRSFRNNE..E
HvXTH4                  AYGVG......GREQQFKLWFDPSAEYHTYSIVWNPRRIT..IEVDGVTIRSYDNNE..E
HvXTH14                 ASGDG......KKEHQFYLWFDPSADFHTYKITWNPKNII..FEVDGVPVRTFKKYD...
HvXTH1                  ASGDG......KKEHQFYLWFDPTADFHTYKIVWNPKNII..FQVDDVPVRTFKKYD...
HvXTH6                  ANGDG......KKEHQFDLWFDPAADYHTYTIIWNPENIL..FKVDNLFIRSFKRFA...
HvXTH5                  ANGDG......KKEHQFDLWFDPAADYHTYTIIWNPENIL..FKVDNLFIRSFKRFA...
HvXTH7                  VNGDG......KKEHQFDLWFDPAADYHTYTIIWNPENIL..FKVDNLFIRSFKRFA...
HvXTH2                  AAGVG......GKEMQFRPWFDPTDGYHNYTIAWTPCAVV..WYVDGAPIRAFRNYER.T
HvXTH15                 VNGDG......DREQRLSLWFDPAADFHEYKILWNPYHLV..ILVDDVPIRVLRNLTGQV
2UWA                    IEGSGDYN.IIGREMRIHLWFDPTQDYHNYAIYWTPSEII..FFVDDVPIRRYPRK....
HvXTH10                 VRGTGDAHPIVGREMRFHLWFDPAAAFHHYAVLWNPDEIV..FLVDDVPVRRYQKK....
HvXTH16                 VRGSGDGR.IIGREMRFHLWFDPTAGFHNYAILWNPDAIT..FFVDDVPIRRYERK....
HvXTH12                 GNGST....SRGREERYLLPFDPTLAAHRYSILWAPTHII..FYVDDTAIREVVRHP..G
HvXTH9                  GDGST....AVGREERYGLWFDPTHDFHRYAILWTNRTIV..FYVDGTPIREVVRSE..A
HvXTH11                 GNGST....GAGREERYDLPFDPTDDFHHYSILWTQHRII..FYVDETPIREVVRTE..A
HORVU4Hr1G085940.2      QNKER.....TDRIARISLY............CWMASSVC.ASLVE...LGELKRLS...
```

```
                                      170       180       190           200       210
HvEG16_Hordeum_vul    .GEEPWPE.KPMFLYASFWDASGVDEGRWTGTYHGR.......DAPYVCSYRDVRVP.VA
TaEG16_Triticum_ae    ............................................................
TaEG16_Triticum_ae    .G.EPWPE.KPMFLYASFWDASGVDEGRWTGTYHGR.......DAPYVCSYRDVRVP.VA
OsEG16_Oryza_sativ    .G.EPWPE.KPMFLYASVWDASHINDGKWTGTYHGR.......DAPYVCSYRDIRVP.LA
OsEG16_Oryza_sativ    .G.EPWPE.KPMFLYASVWDASHINDGKWTGTYHGR.......DAPYVCSYRDIRVP.LA
OsEG16_Oryza_sativ    .G.EPWPE.KPMFLYASVWDASHINDGKWTGTYHGR.......DAPYVCSYRDIRVP.LA
TaEG16_Triticum_ae    .G.EPWPE.KPMYLYASLWDASDVDEGRWTGTYHGR.......DAPYVFSYRDVRVPAVA
ZmEG16_Zea_mays_GR    .G.EPWPE.KPMYLYASVWDASHIADGAWTGTYHGR.......DEPYVCSYKDVRVPTAE
ZmEG16_Zea_mays_NP    .G.EPWPE.KPMYLYASVWDASHIADGAWTGTYHGR.......DAPYVCSYKDVRVPTAE
ZmEG16_Zea_mays_Zm    .G.EPWPE.KPMYLYASVWDASHIADGAWTGTYHGR.......DAPYVCSYKDVRVPTAE
VvEG16                .G.EGFPQ.KPMFLYASVWDASYIDEGRWTGPYVGC.......DAPYICLYKNVNVP.VG
PtEG16                .G.EGFPE.KPMFLYASIWDASYICDATWTGPYMGC.......DAPYVCLYKDICVP.VG
1UN1                  LG.VKFPFNQPMKIYSSLWNADD.....W.ATRGGLEK.TDWSKAPFIASYRSFHI..DG
HvXTH8                LG.VRYPFDQPMKLYSSLWNADD.....W.ATRGGREK.TDWSKAPFVASYRGFHV..DG
HvXTH22               NG.VPFPTRQPVHVFASIWNAEE.....W.ATQGGRVK.TDWSEAPFVAAYRRFDAS.SA
HvXTH20               KG.IAFPKNQPMRLYSSLWNADD.....W.ATQGGRVK.TDWSHAPFSASYRGFKA..DA
HvXTH19               KG.IAFPKNQPMRLYSSLWNADD.....W.ATQGGRVK.TDWSHAPFSASYRGFKA..DA
HvXTH18               KG.IAFPKNQPMRLYSSLWNADD.....W.ATQGGRVK.TDWSHAPFSASYRGFKA..DA
HvXTH17               KG.IAFPKNQPMRLYSSLWNADD.....W.ATQGGRVK.TDWSHAPFSASYRGFKA..DA
HvXTH23               RG.VSFPKSQPMRLYASLWNADD.....W.ATQGGRVK.TDWSKAPFVASFRNFNA..DA
HvXTH21               TG.VPYLRSQAMRVYASVWDAEE.....W.ATQGGRVR.TDWSRAPFVASYKGLAA..SG
HvXTH24               NG.SPYPNYQPMRVFSTIWNADD.....W.ATQGGRVK.TDWTQAPFTAYFRNYKA..TS
HvXTH3                PN.KFFPIMKPMYVFSSIWNADD.....W.ATRGGLEK.TDWTKGPFVSSYSDFTA..DA
HvXTH13               HG.VAFPTWQKMRLHGSLWNADD.....W.ATQGGRVK.TDWSGAPFFARYRNLRA..SW
HvXTH4                HG.VPFPAWQQQRVYGSLWNADD.....W.ATQGGRVK.TDWKLAPFVSYYRNYNI..TY
HvXTH14               .G.LPFPSARPMTVHATLWDGSY.....W.ATQHGTVK.IHWRHDPFVVPYQGYHA..NG
HvXTH1                .D.LPYPSSQPMTVHATLWDGSY.....W.ATRHGDVK.IDWTQAPFVVNYRGYTS..NG
HvXTH6                .G.LPYPTSKPMRLHATLWDGSY.....W.ATEKGKIP.INWSNAPFVVSYRNYYA..NA
HvXTH5                .G.LPYPTSKPMRLHATLWDGSY.....W.ATEKGKIP.INWSNAPFVVSYRNYYA..NA
HvXTH7                .G.IPYAGSKPMRLHATLWDGSY.....W.ATEKGKVP.IDWSNAPFNVLYKNYYA..NA
HvXTH2                HG.VAFPTTRPMHAYSSIWAAED.....W.ATQGGRVR.ADWTRAPFVASYRGIDL..DI
HvXTH15               AE.YEFPAKR.MAVRASLWDGSD.....W.ATDGGRTK.IDWGRAPFTAGFRGFDV..DA
2UWA                  SD.ATFPL.RPLWVYGSVWDASS.....W.ATENGKYK.ADYRYQPFVGKYEDFKL..GS
HvXTH10               VE.ATFPE.REMWAYGSVWDASD.....W.ATDGGRYR.SDYRYQPFVSGFKDFKV..AG
HvXTH16               TE.LTFPD.RPMWAYGSIWDASD.....W.ATDHGRHR.ADYRYQPFVARFDRFVV..AG
HvXTH12               MG.GDFPA.KPMAAYATIWDGSA.....W.ATEGGKYK.VNYKYAPFASDFSDLSL..RG
HvXTH9                MG.AQFPS.KPMSLYATIWDGSS.....W.ATSGGRYK.VEYKYAPYVAEFTDLEL..RG
HvXTH11               MG.AAFPS.KPMSLYATIWDGSA.....W.ATLGGRYR.ANYKYAPFVAEFGDLVL..HA
HORVU4Hr1G085940.2    .........KAMRKRAKELRGADKYED...EQYLGKMKQSDDRLLALVKAGMDVVV....

                          220                 230           240
HvEG16_Hordeum_vul    LSTEEEEEECQ.................DDADAGDEAD.......AAGAAEE....EEE.
TaEG16_Triticum_ae    ............................................................
TaEG16_Triticum_ae    LSAEEEEEEE..................................................
OsEG16_Oryza_sativ    LSLEDEEDPYKCACVGDASAAIAAADAAEQVDAGDAPA.......AAAAADA....AEEV
OsEG16_Oryza_sativ    LSLEDEEDPYKCACVGDASAAIAAADAAEQVDAGDAPA.......AAAAADA....AEEV
OsEG16_Oryza_sativ    LSLEDEEDPYKCACVGYASAAIAAADAAEQVDAGDAPA.......AAAAADA....AEEV
TaEG16_Triticum_ae    LSAGEEEEWQ..................DDTNAGDEAD.......AAATTCE....D...
ZmEG16_Zea_mays_GR    HSVED......................AAHAPAGDPIA.......AEAAAAA....EE..
ZmEG16_Zea_mays_NP    HSVED......................AAHAPAGDPVA.......ADAAAAA....EE..
ZmEG16_Zea_mays_Zm    HSVED......................AAHAPAGDPVA.......ADAAAAA....EE..
VvEG16                TAVE........................................................
PtEG16                TAVEC.......................................................
1UN1                  C..............................EASVEAK.......FCATQG.....ARWW
HvXTH8                C..............................EASAEAK.......LCATQG.....ARWW
HvXTH22               CVW............................HGGASPT.......RCGGDHLPSSASSWM
HvXTH20               CVVTAGGRPH...CG.........A.....SVGTDVAP.......GTGAA......GEWY
HvXTH19               CVVTAGGRPR...CG.........A.....SVGTDVAP.......GTGAA......GEWY
HvXTH18               CVVTAGGRPR...CG.........A.....SMGTEAAP.......GTGASGA...AGEWY
HvXTH17               CVVTVGGRPR...CG.........A.....SIGTDAAP.......GTGGAAA...VGDWY
HvXTH23               CVMS.GGAQR...C................PAGTMEAS.......AAGG.......GSWW
HvXTH21               C...............................ASQDAA.......ACANSN.....GAWM
HvXTH24               C..............................SQGQNSN.......VCGQSSP....NGLF
HvXTH3                CAW............................PSGPAPP.......ACAAATG....DSWW
HvXTH13               C..............................RPSPGVA.......WCGDEPPG...STWF
HvXTH4                C..............................RPSPGVS.......WCGAEPAG...SPVF
HvXTH14               CVH............................DKATNKT.......SCPAGS.....DAWM
HvXTH1                CVS............................NGGS..S.......ACPAGS.....DAWM
HvXTH6                CVS............................G.....G.......ACHAGS.....DRWM
HvXTH5                CVS............................G.....G.......ACHAGS.....DRWM
HvXTH7                CAS..................................G.....G.ACHAGS.....DGWM
HvXTH2                C........E...C.................YGGDCVY.......TCA........GAFR
HvXTH15               C.................................DNAS.......STPCDST....DLWW
2UWA                  CTV............................EAAS..........SCN........PASV
HvXTH10               CEV............................GAPA..........SCR........PVPA
HvXTH16               CGP............................GAPP..........SCR........PVRA
HvXTH12               CRV............................ADPASPALRLAGGDGCDLLGL...MTADY
HvXTH9                CAS............................HDRAQPA.......SC.............
HvXTH11               CPVNR....................IY....HSAA..A.......ACG........TPWY
HORVU4Hr1G085940.2    .............................................AVGLLQLA.......
```

```
                                                                        250
HvEG16_Hordeum_vul   ...............................................EMDAGDGED....
TaEG16_Triticum_ae   ............................................................
TaEG16_Triticum_ae   ............................................................
OsEG16_Oryza_sativ   D....AGDAPAATAAADVAEQ.........VDAGDVPASAAAADAVKEVDAGAGKD....
OsEG16_Oryza_sativ   D....AGDAPAATAAADVAEQ.........VDAGDVPASAAAADAVKEVDAGAGKD....
OsEG16_Oryza_sativ   D....AGDAPAATAATDVAEQ.........VDAGDVPASAAAADAVKEVDAGAGKD....
TaEG16_Triticum_ae   ............................................................
ZmEG16_Zea_mays_GR   ...............................................EKDAGASEV....
ZmEG16_Zea_mays_NP   ...............................................EKDAGAGEV....
ZmEG16_Zea_mays_Zm   ...............................................EKDAGA.......
VvEG16               ............................................................
PtEG16               ...............................................SCDS........
1UN1                 DQKEFQDLDAFQYRRLSWVRQ.KYTIYNYCTDR....SRYPS..MPPECKRD........
HvXTH8               DQPEFQDLDAAQYRRLAWVRK.EHTIYNYCTDR....ERYAA..MSPECKRD........
HvXTH22              G....QRLDWWSWMTLNWVRM.NYMTYDYCADR....KRYPH.GFPAECIIPIGRI....
HvXTH20              N....QELDLTRQQRMRWVQS.NYMIYNYCTDP....KRFAQ.GVPAECSM.........
HvXTH19              N....QELDLTRQQRMRWVQS.NYMIYNYCTDP....KRFAQ.GVPAECSM.........
HvXTH18              N....QELDLTLQQRMRWVQS.NYMIYNYCTDP....KRVAK.GVPAECSM.........
HvXTH17              N....QELDLTRQQRMRWVQS.NYMIYNYCTDP....KRVAK.GVPAECSM.........
HvXTH23              N....QELSGMGYRRMRWVQR.KFMIYNYCTDP....KRVAQ.GVPAECKLR........
HvXTH21              Y....QELDATALDRLQWVQK.NYMIYNYCTDT....WRFKD.GAPPECASK........
HvXTH24              N....QQQDQMQQQQVKEVDA.KYKVYDFCDDS....KRRI..GSSEDCQSQ........
HvXTH3               DQPPAWALDDGQRRDSGWVAR.NLVIYDYCGDR....KRFPT..VPEECALRTTTS....
HvXTH13              E....RGLDA...AALRRARD.AHMIYDYCKDL....QRYKGSGLPKECVVD........
HvXTH4               N......LAPKARADMQWVRDMGYVIYDYCTDR...SNRYNDTTRPKECSLPPRP.....
HvXTH14              H....RELDDGELSTVAWAER.NCLSYNYCADG....WRFPK.GFPGECGRK........
HvXTH1               S....TELDAKALGTVAWAES.KYMSYDYCTDG....WRFPN.GFPAECSRRN.......
HvXTH6               R....KQLDGDEWGTVKWAER.SYMRYNYCEDG....YRFPQ.GLPAECNRY........
HvXTH5               K....KQLDGAEWGTVKWAER.SYMRYNYCEDG....YRFPQ.GLPAECNRY........
HvXTH7               N....RQLDGSEWGTVKWAEQ.NYMRYNYCADG....YRFPQ.GFPAECSRY........
HvXTH2               G...CGGLTGDQRGKMQWVQD.NYRIYDYCADHE..AGKVPG....VECSLPQY......
HvXTH15              NARRHRRLSVREQAAYENVRR.TYMNYDYCADK....DRFQNGKLPVECSYTT.......
2UWA                 S.P.YGQLSQQQVAAMEWVQK.NYMVYNYCDDP....TRDHT..LTPEC...........
HvXTH10              G.P.GGGLSAQQSAAMSWAQQ.RAMVYYYCQDG....SKDRS..NYPEC...........
HvXTH16              S.PVGTGLTRQQYAAMRWAQQ.RHMVYYYCQDF....RRDRS..LTPEC...........
HvXTH12              .....AVMTPQKRAAMRAFRA.RRMTYTVCYDA....ARYAA.GPFPECDNSDEERGTFW
HvXTH9               E.P..EGMPARQRAAMERVRA.RHMTYGYCYDR....ARYPA..PLPECRVGAEAA.MYL
HvXTH11              E.PVAAALSGEQRASMSAFRR.GHMSYSYCHDR....RRYPV..ALSECDVAVLPR.LFG
HORVU4Hr1G085940.2   ..P..KKVTPRVTGAFGFVTS.....LISCYQQ..LPARAPL..................
```

```
HvEG16_Hordeum_vul   .....................................
TaEG16_Triticum_ae   .....................................
TaEG16_Triticum_ae   .....................................
OsEG16_Oryza_sativ   .....................................
OsEG16_Oryza_sativ   .....................................
OsEG16_Oryza_sativ   .....................................
TaEG16_Triticum_ae   .....................................
ZmEG16_Zea_mays_GR   .....................................
ZmEG16_Zea_mays_NP   .....................................
ZmEG16_Zea_mays_Zm   .....................................
VvEG16               .....................................
PtEG16               .....................................
1UN1                 ...................RDI...............
HvXTH8               ...................RDV...............
HvXTH22              .....................................
HvXTH20              .....................................
HvXTH19              .....................................
HvXTH18              .....................................
HvXTH17              .....................................
HvXTH23              .....................................
HvXTH21              .....................................
HvXTH24              .....................................
HvXTH3               .....................................
HvXTH13              .....................................
HvXTH4               .....................................
HvXTH14              .....................................
HvXTH1               .....................................
HvXTH6               .....................................
HvXTH5               .....................................
HvXTH7               .....................................
HvXTH2               .....................................
HvXTH15              .....................................
2UWA                 .....................................
HvXTH10              .....................................
HvXTH16              .....................................
HvXTH12              AWGESKTVVMKTRGRGRRGRGSRAGAGARGRAGAASS
HvXTH9               PSGEA.......RSSDRR.R.....HGKRHRRADSAL
HvXTH11              PDGMK.......YGGDRRHR.....RGGRGRRSDVVM
HORVU4Hr1G085940.2   ................................AKVKA
```
