# Supplementary material for: Genome-Wide Identification, Characterization and Expression Analysis of Xyloglucan Endotransglucosylase/Hydrolase Genes Family in Barley (Hordeum vulgare)
Source: Molecules. 2019 May 20;24(10):1935. doi: 10.3390/molecules24101935 (PMC6572274; doi:10.3390/molecules24101935)
Supplement: Supplementary file 1 [file molecules-24-01935-s001.zip › Supplementary File 8 Table S2. Plant tissues for RNA.docx]

**Supplementary file S2: Table S2 Plant tissues for RNA-seq**

| Plant organization | Tissues (abbreviation) | | | Details |  |
| --- | --- | --- | --- | --- | --- |
| Vegetative tissues | | LEA | Leaf tissue (10 cm shoot stage) | |  |
|  |  | ROO | Root tissue (10 cm shoot stage) | |  |
|  |  | ROO2 | Root tissue | |  |
|  |  | NOD | Third stem internode (developing tillers at six-leaf stage) | |  |
|  |  | SEN | Senescing leaf | |  |
|  |  | ETI | Isolate etiolated leaf (grown to 10 dap in the dark) | |  |
|  |  | EPI | Epidermal strips | |  |
| Inflorescence tissues | | INF1 | Whole developing inflorescence tissue (5mm) | |  |
|  |  | INF2 | Whole developing inflorescence tissue (1-1.5cm) | |  |
|  |  | LOD | Isolate lodicule dissected from inflorescences | |  |
|  |  | LEM | Lemma dissected from inflorescences | |  |
|  |  | PAL | Palea dissected from inflorescences | |  |
|  |  | RAC | Rachis | |  |
| Developing grain tissues | | CAR5 | Whole developing grain (5 DPA, days post-anthesis) | |  |
|  |  | CAR15 | Whole developing grain (15 DPA) | |  |
| Germinating grain tissues | | EMB | 4-day embryos tissue from geminating grains | |  |
