# Supplementary material for: Genome-Wide Identification, Characterization and Expression Analysis of Xyloglucan Endotransglucosylase/Hydrolase Genes Family in Barley (Hordeum vulgare)
Source: Molecules. 2019 May 20;24(10):1935. doi: 10.3390/molecules24101935 (PMC6572274; doi:10.3390/molecules24101935)
Supplement: Supplementary file 1 [file molecules-24-01935-s001.zip › Supplementary File 1 Protein sequences of XTHs employed in the phylogenetic analysis .docx]

**Supplementary File S4: Protein sequences of XTHs employed in the phylogenetic analysis**

>1GBG

MSYRVKRMLMLLVTGLFLSLSTFAASASAQTGGSFYEPFNNYNTGLWQKADGYSNGNMFNCTWRANNVSMTSLGEMRLSLTSPSYNKFDCGENRSVQTYGYGLYEVNMKPAKNVGIVSSFFTYTGPTDGTPWDEIDIEFLGKDTTKVQFNYYTNGVGNHEKIVNLGFDAANSYHTYAFDWQPNSIKWYVDGQLKHTATTQIPQTPGKIMMNLWNGAGVDEWLGSYNGVTPLSRSLHWVRYTKR

>HvXTH22

MLRGSLRWLLVLAVVVAASAGKAGRGLHRDFDAVWGKRNARFFDEGRVVELALDRETGSRLQSKDRYLFGRFDLDIRLVAGESAGTITSFYICTGGARHDEVDFEFLGNVSGEPYILHTNIFSDGKGEREQQFVLWFDPTADFHTYSILWNPLNIILYIDGTPIRVFKNNEANGVPFPTRQPVHVFASIWNAEEWATQGGRVKTDWSEAPFVAAYRRFDASSACVWHGGASPTRCGGDHLPSSASSWMGQRLDWWSWMTLNWVRMNYMTYDYCADRKRYPHGFPAECIIPIGRI

>HvXTH20

MARMAVSVLAILLAWCALAAASFDKEFDITWGDGRGKILNNGQLLTLALDKVSGSGFQSKHEYLFGKIDMQLKLVPGNSAGTVTAYYLSSQGPTHDEIDFEFLGNVTGEPYTLHTNVFTQGQGQREQQFRLWFDPTNDFHTYSILWNPKHIIFMVDDMPIRDFKNLEGKGIAFPKNQPMRLYSSLWNADDWATQGGRVKTDWSHAPFSASYRGFKADACVVTAGGRPHCGASVGTDVAPGTGAAGEWYNQELDLTRQQRMRWVQSNYMIYNYCTDPKRFAQGVPAECSM

>HvXTH16

MASLSLLPAMALLLLAMAVASSDAQPSPGYYPSSRFRPVAFNRGYSNKWGPQHQTVSGDHSAITIWLDRTCGSGFKSKHAYRNGYFATRIKLPAGYTAGTNTAFYLSNNEAHPGFHDEVDMEFLGTIPGEPYTLQTNVYVRGSGDGRIIGREMRFHLWFDPTAGFHNYAILWNPDAITFFVDDVPIRRYERKTELTFPDRPMWAYGSIWDASDWATDHGRHRADYRYQPFVARFDRFVVAGCGPGAPPSCRPVRASPVGTGLTRQQYAAMRWAQQRHMVYYYCQDFRRDRSLTPEC

>HvXTH19

MARMAVSVLSILLATCALAAASFDKEFDVTWGDGRGKILNNGQLLTLGLDKVSGSGFQSKHEYLFGKIDMQLKLVPGNSAGTVTAYYLSSQGPTHDEIDFEFLGNVTGEPYTLHTNVFTQGQGQREQQFRLWFDPTNDFHTYSILWNPKHIIFMVDDMPIRDFKNLEGKGIAFPKNQPMRLYSSLWNADDWATQGGRVKTDWSHAPFSASYRGFKADACVVTAGGRPRCGASVGTDVAPGTGAAGEWYNQELDLTRQQRMRWVQSNYMIYNYCTDPKRFAQGVPAECSM

>HvXTH15

MASSVRQPWLLLLLVLLPVMATAAVFDDNYAPTWGADGYHLVDQGTEIRLTMDRNSGAGFHSKSTYGSGFFHMRIKVPGGYTAGVVTAFYLASETPYDGSDRDEVDFEFLGNVDGENITLQTNVFVNGDGDREQRLSLWFDPAADFHEYKILWNPYHLVILVDDVPIRVLRNLTGQVAEYEFPAKRMAVRASLWDGSDWATDGGRTKIDWGRAPFTAGFRGFDVDACDNASSTPCDSTDLWWNARRHRRLSVREQAAYENVRRTYMNYDYCADKDRFQNGKLPVECSYTT

>HvXTH11

MASSSSCPPPSPRPSRLLPVLVATVVLLGRGGEARQPAPLHGVVRSMAFDEGYTQLFGSGNLALRREGKRVHLALDESTGSGFASQDRFLHGFFSAAVKLPADYAAGVVVAFYLSNADVYEKTHDELDFEFLGNVRGREWRVQTNVYGNGSTGAGREERYDLPFDPTDDFHHYSILWTQHRIIFYVDETPIREVVRTEAMGAAFPSKPMSLYATIWDGSAWATLGGRYRANYKYAPFVAEFGDLVLHACPVNRIYHSAAAACGTPWYEPVAAALSGEQRASMSAFRRGHMSYSYCHDRRRYPVALSECDVAVLPRLFGPDGMKYGGDRRHRRGGRGRRSDVVM

>HvXTH3

MKAPSGLGLAYKKAVSCALCFAPDQSISTLLHSPPLCLPLCGFAVSRPTFVVGGTVFVSSWGWGAMGPWRRPCVGALLACAAIAASCCCFQLQGADAAASPSFGDNFEITGAKDHVKTSPDGQTWYLSLDNKTGVGFQTKQKYLFGWFSMKLKLVGNDSAGVVTAYYMCSDLDAAPERDELDFEFLGNRTGEPYIIQTNVYRSGVGGREMRHSLWFDPTADFHSYSILWNPKQIVFFVDKVAIREYRNSAKPNKFFPIMKPMYVFSSIWNADDWATRGGLEKTDWTKGPFVSSYSDFTADACAWPSGPAPPACAAATGDSWWDQPPAWALDDGQRRDSGWVARNLVIYDYCGDRKRFPTVPEECALRTTTS

>HvXTH2

MGKPGALVPVVALAFALVLGLELVSGGNFYEECDATWEPQNCWTYDGGNSLSLALVSNSSGLSSTSQLLYFSWSSPLNESMCGCSGSMIRSKRQFIYGTVSTMIQLVKGDSAGTVTTYYTSSVGDDHDEIDFEFLGNETGQPYTLHTNVYAAGVGGKEMQFRPWFDPTDGYHNYTIAWTPCAVVWYVDGAPIRAFRNYERTHGVAFPTTRPMHAYSSIWAAEDWATQGGRVRADWTRAPFVASYRGIDLDICECYGGDCVYTCAGAFRGCGGLTGDQRGKMQWVQDNYRIYDYCADHEAGKVPGVECSLPQY

>HvXTH14

MAPRSDLLAALALALLAASVLSTGAKADFDDQFEVIGDRDHIGYRDDGNDKGQEFSLELDQESGSGFKSKAKYLFGEFQVRMKLVDGNSAGTVTSFYLTSGESATHDEIDIEFMGNSSGDPYVMNTNVWASGDGKKEHQFYLWFDPSADFHTYKITWNPKNIIFEVDGVPVRTFKKYDGLPFPSARPMTVHATLWDGSYWATQHGTVKIHWRHDPFVVPYQGYHANGCVHDKATNKTSCPAGSDAWMHRELDDGELSTVAWAERNCLSYNYCADGWRFPKGFPGECGRK

>HvXTH12

MEMTARFLAAAAACVWLAAAASAFDVPTVAFEEGFSPLFGDGNLVRARDDRAARLLLDRRSGSGFISSDYYLHGFFSASIKLPRDYTAGVVVAFYLSNGDVYEKTHDELDFEFLGSRWGGQWRVQTNVYGNGSTSRGREERYLLPFDPTLAAHRYSILWAPTHIIFYVDDTAIREVVRHPGMGGDFPAKPMAAYATIWDGSAWATEGGKYKVNYKYAPFASDFSDLSLRGCRVADPASPALRLAGGDGCDLLGLMTADYAVMTPQKRAAMRAFRARRMTYTVCYDAARYAAGPFPECDNSDEERGTFWAWGESKTVVMKTRGRGRRGRGSRAGAGARGRAGAASS

>HvXTH10

MAMMQIRRPHDAISHLMVIVVGAVILLQGEAQPSPGYYPSSKVSSTPFSQWYSTLWGPQHQSLSPDQTALTLWMDRSSGSGFKSKRSYRNGYFGVSMKVQPGYTAGVNTAFYLSNNEVYPGYHDEIDVELLGTVPGEPYTLQTNVYVRGTGDAHPIVGREMRFHLWFDPAAAFHHYAVLWNPDEIVFLVDDVPVRRYQKKVEATFPEREMWAYGSVWDASDWATDGGRYRSDYRYQPFVSGFKDFKVAGCEVGAPASCRPVPAGPGGGLSAQQSAAMSWAQQRAMVYYYCQDGSKDRSNYPEC

>HvXTH13

MAPSLPSSSSCWHSALLVAMLVLVVVMDQVAMAYLDDDIEVVWGDDHSFFYMDDAGDDEILALCLDETHGSGFHTKEAYLYARFDVDLMLVPDNSAGTVTTLYLMPEDVPWDYHDEVDLEFLGNVTGEPYTLHTNIFANGVGNREEQFRLWFDPTADFHTYSIDWNPKRITILVDGVPIRSFRNNEEHGVAFPTWQKMRLHGSLWNADDWATQGGRVKTDWSGAPFFARYRNLRASWCRPSPGVAWCGDEPPGSTWFERGLDAAALRRARDAHMIYDYCKDLQRYKGSGLPKECVVD

>HvXTH17

MARMGASVLVILLASCALAAASFDKEFDVTWGDGRGKILNNGQLLMLGLDKVSGSGFQSKREYLFGKIDMQLKLVPGNSAGTVTAYYLSSQGPTHDEIDFEFLGNVTGEPYTLHTNVFTQGQGQREQQFRLWFDPTNDFHTYSILWNPKHIIFLVDDMPIRDFRNMEGKGIAFPKNQPMRLYSSLWNADDWATQGGRVKTDWSHAPFSASYRGFKADACVVTVGGRPRCGASIGTDAAPGTGGAAAVGDWYNQELDLTRQQRMRWVQSNYMIYNYCTDPKRVAKGVPAECSM

>HvXTH21

MASGPSRTVPCSVLPLLLLLAGVARAAGNFYQDVDITWGDGRGKILGGGDLLTLSLDRASGSGFQSKNQYLYGRFDMQIKLVPGDSAGTVATFYLSSQGSAHDEIDFEFLGNASGQPYTVHTNVYSQGKGGREQQFRMWFDPTADFHTYSVLWNPTHILFYVDGTPIREHRNREAATGVPYLRSQAMRVYASVWDAEEWATQGGRVRTDWSRAPFVASYKGLAASGCASQDAAACANSNGAWMYQELDATALDRLQWVQKNYMIYNYCTDTWRFKDGAPPECASK

>HvXTH24

MGQARAYLLASLAAFYLVALAIPQVTADMTDEVNLLWGNCKVQRDGTGRQTVAMSLDRWTTSGFSSKIKYLFGRIDMEIKLMPGNSAGTVTTFYMMSEGPWQFHDEIDLEFLGNSTGNPYTLHTNVYARGVGSREKGYRLWFDPSQDFHTYSIIWTQQYIRFLVDNKLIRQIKNKMMNGSPYPNYQPMRVFSTIWNADDWATQGGRVKTDWTQAPFTAYFRNYKATSCSQGQNSNVCGQSSPNGLFNQQQDQMQQQQVKEVDAKYKVYDFCDDSKRRIGSSEDCQSQ

>HvXTH23

MRTVELGIVAMACLVAVARAGNFFQDSEMSWGDGRGKVVDGGRGLDLTLDKTSGSGFQSKSEYLFGKIDMQIKLVPGNSAGTVTTFYLSSQGTAHDEIDFEFLGNVTGEPYTLHTNVFAQGQGQREQQFRLWFDPTKAFHTYSIIWNPQHVIFAVDGTAIRDFKNHEARGVSFPKSQPMRLYASLWNADDWATQGGRVKTDWSKAPFVASFRNFNADACVMSGGAQRCPAGTMEASAAGGGSWWNQELSGMGYRRMRWVQRKFMIYNYCTDPKRVAQGVPAECKLR

>HvXTH18

MARMGASVLSILLASCALAAASFDKEFDVTWGDGRGKILNNGQLLTLGLDKVSGSGFQSKHEYLFGKIDMQLKLVPGNSAGTVTAYYLSSQGPTHDEIDFEFLGNVTGEPYTLHTNVFTQGQGQREQQFRLWFDPTNDFHTYSILWNPKHIIFMVDDMPIRDFKNLEGKGIAFPKNQPMRLYSSLWNADDWATQGGRVKTDWSHAPFSASYRGFKADACVVTAGGRPRCGASMGTEAAPGTGASGAAGEWYNQELDLTLQQRMRWVQSNYMIYNYCTDPKRVAKGVPAECSM

>HvXTH1

MARPSFSLHLCLAVLALAAAASEAGFYDQFDVVGSGNNVRVNDDGIAQQVALTLDQGNGGSGFSSKDKYLYGEFSVQMKLIGGNSAGTVTSFYLTSGEGDGHDEIDIEFMGNLSGDPYVMNTNVWASGDGKKEHQFYLWFDPTADFHTYKIVWNPKNIIFQVDDVPVRTFKKYDDLPYPSSQPMTVHATLWDGSYWATRHGDVKIDWTQAPFVVNYRGYTSNGCVSNGGSSACPAGSDAWMSTELDAKALGTVAWAESKYMSYDYCTDGWRFPNGFPAECSRRN

>HvXTH6

MAQRFLAVLAVALALSQVASAKSWLDKRFNTDGTVRTGYDASGQQVVMLNLNQQSGAAGFNSKQQYLYGEFSIQMKLIPGNSAGTVSCFYLSSGDDEWRDEIDMEFMGNSSGHPVVLNTNVWANGDGKKEHQFDLWFDPAADYHTYTIIWNPENILFKVDNLFIRSFKRFAGLPYPTSKPMRLHATLWDGSYWATEKGKIPINWSNAPFVVSYRNYYANACVSGGACHAGSDRWMRKQLDGDEWGTVKWAERSYMRYNYCEDGYRFPQGLPAECNRY

>HvXTH5

MARRLLAVLAVALALLQAASAKSWLDKRFNTDGTVRTGYDASGQQVVMLNLNQQSGAAGFNSKQQYLYGEFSIQMKLIPGNSAGTVSCFYLSSGDDEWRDEIDMEFMGNSSGHPVVLNTNVWANGDGKKEHQFDLWFDPAADYHTYTIIWNPENILFKVDNLFIRSFKRFAGLPYPTSKPMRLHATLWDGSYWATEKGKIPINWSNAPFVVSYRNYYANACVSGGACHAGSDRWMKKQLDGAEWGTVKWAERSYMRYNYCEDGYRFPQGLPAECNRY

>HvXTH7

MSNTSTLSSGDGDGHDEIDMEFMGNSSGPGHPVVLNTNVWVNGDGKKEHQFDLWFDPAADYHTYTIIWNPENILFKVDNLFIRSFKRFAGIPYAGSKPMRLHATLWDGSYWATEKGKVPIDWSNAPFNVLYKNYYANACASGGACHAGSDGWMNRQLDGSEWGTVKWAEQNYMRYNYCADGYRFPQGFPAECSRY

>HvXTH4

MAPALPCSRPKLLLLCVALAFLLAVDVGRADIYKDIQIIWSADHTYYFMDGDSEALALSLDFNRGSAFKSNDMYLYARIDIDIKLVEGNSAGTVCTVYTISEGPWDIHDEIDLEFLGNSTGEPYTLHTNIFAYGVGGREQQFKLWFDPSAEYHTYSIVWNPRRITIEVDGVTIRSYDNNEEHGVPFPAWQQQRVYGSLWNADDWATQGGRVKTDWKLAPFVSYYRNYNITYCRPSPGVSWCGAEPAGSPVFNLAPKARADMQWVRDMGYVIYDYCTDRSNRYNDTTRPKECSLPPRP

>HvXTH9

MACHFLLAVLLASSSWVAASSGAAADDVMVPRPTTAAALTFREGYTQLFGDSNLRLHGDGKRVHISLDERTGSGFASQGAYFHGFFSASIKLPSDYAAGVVVAFYVSNGDVYEKTHDELDFEFLGNVRGKEWRVQTNVYGDGSTAVGREERYGLWFDPTHDFHRYAILWTNRTIVFYVDGTPIREVVRSEAMGAQFPSKPMSLYATIWDGSSWATSGGRYKVEYKYAPYVAEFTDLELRGCASHDRAQPASCEPEGMPARQRAAMERVRARHMTYGYCYDRARYPAPLPECRVGAEAAMYLPSGEARSSDRRRHGKRHRRADSAL

>HvXTH8

MKATAGALLAVVATVLLRGIAAAPPRKPVDVPFEKNYVPTWAEDHIHYVNGGREVQLSLDKTTGTGFQTRGSYLFGHFSMHIKLVGGDSAGTVTAFYVPSQNSEHDEIDFEFLGNRTGQPYILQTNVFSGGKGDREQRIYLWFDPTKDYHSYSVLWNLYMIAFFVDDTPIRVFKNSKDLGVRYPFDQPMKLYSSLWNADDWATRGGREKTDWSKAPFVASYRGFHVDGCEASAEAKLCATQGARWWDQPEFQDLDAAQYRRLAWVRKEHTIYNYCTDRERYAAMSPECKRDRDV

>AtXTH33

MKIMWETAVVFCLCSLSLVSSHSRKFTTPNVTRLTDQFSKIAIENGFSRRFGAHNIQVNGSLAKLTLDKSSGAGLVSKNKYHYGFFSARLKLPAGFASGVVVAFYLSNAETYPKSHDEIDIELLGRSRRDDWTIQTNVYANGSTRTGREEKFYFWFDPTQAFHDYTLIWNSHHTVFLVDNIPVRQFPNRGAFTSAYPSKPMSLYVTVWDGSEWATKGGKYPVNYKYAPFVVSVADVELSGCSVNNGSSTGSGPCTKSGGSISSLDPVDGQDFATLSKNQINAMDWARRKLMFYSYCSDKPRYKVMPAECN

>AtXTH8

METERRIITSCSAMTALFLFMTALMASSSIAATPTQSFEDNFNIMWSENHFTTSDDGEIWNLSLDNDTGCGFQTKHMYRFGWFSMKLKLVGGDSAGVVTAYYMCSENGAGPERDEIDFEFLGNRTGQPYIIQTNVYKNGTGNREMRHSLWFDPTKDYHTYSILWNNHQLVFFVDRVPIRVYKNSDKVPNNDFFPNQKPMYLFSSIWNADDWATRGGLEKTDWKKAPFVSSYKDFAVEGCRWKDPFPACVSTTTENWWDQYDAWHLSKTQKMDYAWVQRNLVVYDYCKDSERFPTLPWECSISPWA

>AtXTH28

MGFITRFLVFMSLFTSLVSGFALQKLPLIQFDEGYTQLFGDQNLIVHRDGKSVRLTLDERTGSGFVSNDIYLHGFFSSSIKLPADYSAGVVIAFYLSNGDLYEKNHDEIDFEFLGNIRGREWRIQTNIYGNGSTHLGREERYNLWFDPTEDFHQYSILWSLSHIIFYVDNVPIREVKRTASMGGDFPAKPMSLYSTIWDGSKWATDGGKYGVNYKYAPYVSQFTDLILHGCAVDPTEKFPSCKDEAVQNLRLASEITESQRNKMEIFRQKHMTYSYCYDHMRYKVVLSECVVNPAEAKRLRVYDPVTFGGIPHGHRRGKHRSRSRLARTESI

>AtXTH30

MSKSSYNHIFILILCLCLRSSSAFTNLNTLSFEESLSPLFGDANLVRSPDDLSVRLLLDRYTGSGFISSNMYQHGFYSSMIKLPADYTAGVVVAFYTSNGDVFEKTHDELDIEFLGNIKGKPWRFQTNLYGNGSTHRGREERYRLWFDPSKEFHRYSILWTPHKIIFWVDDVPIREVIRNDAMGADYPAKPMALYATIWDASDWATSGGKYKANYKFAPFVAEFKSFSLDGCSVDPIQEVPMDCSDSVDFLESQDYSSINSHQRAAMRRFRQRFMYYSYCYDTLRYPEPLPECVIVPAEKDRFKETGRLKFGGTEARERRRNRRQQRRPEIEIESDPDDRKLL

>AtXTH17

MKLSCGTSFAFLLLFLLAAQSVHVYAGSFHKDVQIHWGDGRGKIHDRDGKLLSLSLDKSSGSGFQSNQEFLYGKAEVQMKLVPGNSAGTVTTFYLKSPGTTWDEIDFEFLGNISGHPYTLHTNVYTKGTGDKEQQFHLWFDPTVNFHTYCITWNPQRIIFTVDGIPIREFKNPEAIGVPFPTRQPMRLYASLWEAEHWATRGGLEKTDWSKAPFTAFYRNYNVDGCVWANGKSSCSANSPWFTQKLDSNGQTRMKGVQSKYMIYNYCTDKRRFPRGVPAECT

>AtXTH27

METLSRLLVFMSLFSGLVSGFALQNLPITSFEESYTQLFGDKNLFVHQDGKSVRLTLDERTGSGFVSNDYYLHGFFSASIKLPSDYTAGVVVAFYMSNGDMYEKNHDEIDFEFLGNIREKEWRVQTNIYGNGSTHSGREERYNLWFDPTEDFHQYSILWSDSHIIFFVDNVPIREVKRTAEMGGHFPSKPMSLYTTIWDGSKWATNGGKYGVNYKYAPYIARFSDLVLHGCPVDPIEQFPRCDEGAAEDMRAAQEITPSQRSKMDVFRRRLMTYSYCYDRARYNVALSECVVNPAEAQRLRVYDPVRFGGIPRRHRNGKHRSKRSRVDGTESI

>AtXTH4

MTVSSSPWALMALFLMVSSTMVMAIPPRKAIDVPFGRNYVPTWAFDHQKQFNGGSELQLILDKYTGTGFQSKGSYLFGHFSMHIKLPAGDTAGVVTAFYLSSTNNEHDEIDFEFLGNRTGQPAILQTNVFTGGKGNREQRIYLWFDPSKAYHTYSILWNMYQIVFFVDNIPIRTFKNAKDLGVRFPFNQPMKLYSSLWNADDWATRGGLEKTNWANAPFVASYKGFHIDGCQASVEAKYCATQGRMWWDQKEFRDLDAEQWRRLKWVRMKWTIYNYCTDRTRFPVMPAECKRDRDA

>AtXTH10

MTLINRSKPFVLLVGFSIISSLLLWVSQASVVSSGDFNKDFFVTWSPTHVNTSNDGRSRTLKLDQESGASFSSIQTFLFGQIDMKIKLIRGSSQGTVVAYYMSSDQPNRDEIDFEFLGNVNGQPYILQTNVYAEGLDNREERIHLWFDPAKDFHTYSILWNIHQIVFMVDQIPIRLYRNHGEKGVAYPRLQPMSVQASLWNGESWATRGGHDKIDWSKGPFVASFGDYKIDACIWIGNTSFCNGESTENWWNKNEFSSLTRVQKRWFKWVRKYHLIYDYCQDYGRFNNKLPKECSLPKY

>AtXTH21

MVSSTLLVMSISLFLGLSILLVVHGKDFNQDIDITWGDGRGNILNNGTLLNLGLDQSSGSGFQSKAEYLYGKVDMQIKLVPGNSAGTVTTFYLKSQGLTWDEIDFEFLGNVSGDPYIVHTNVYTQGKGDREQQFYLWFDPTAAFHNYSILWNPSHIVFYIDGKPIREFKNLEVLGVAYPKNQPMRMYGSLWNADDWATRGGLVKTNWSQGPFVASFMNYNSENACVWSIVNGTTTTSPCSPGDSTSSSSSSTSEWFSQRGMDSSSKKVLRWVQRKFMVYNYCKDKKRFSNGLPVECTAKNKNTKS

>AtXTH32

MGNSLISLLSIFHLLVLWGSSVNAYWPPSPGYWPSSKVGSLNFYKGFRNLWGPQHQRMDQNALTIWLDRTSGSGFKSVKPFRSGYFGANIKLQPGYTAGVITSLYLSNNEAHPGFHDEVDIEFLGTTFGKPYTLQTNVYIRGSGDGKIIGREMKFRLWFDPTKDFHHYAILWSPREIIFLVDDIPIRRYPKKSASTFPLRPMWLYGSIWDASSWATEDGKYKADYKYQPFTAKYTNFKALGCTAYSSARCYPLSASPYRSGGLTRQQHQAMRWVQTHSMVYNYCKDYKRDHSLTPECWR

>AtXTH16

MGRILNRTVLMTLLVVTMAGTAFSGSFNEEFDLTWGEHRGKIFSGGKMLSLSLDRVSGSGFKSKKEYLFGRIDMQLKLVAGNSAGTVTAYYLSSEGPTHDEIDFEFLGNETGKPYVLHTNVFAQGKGNREQQFYLWFDPTKNFHTYSLVWRPQHIIFMVDNVPIRVFNNAEQLGVPFPKNQPMKIYSSLWNADDWATRGGLVKTDWSKAPFTAYYRGFNAAACTVSSGSSFCDPKFKSSFTNGESQVANELNAYGRRRLRWVQKYFMIYDYCSDLKRFPQGFPPECRKSRV

>AtXTH3

MDYMRIFSVFVVTLWIIRVDARVFGGRGIEKFVTFGQNYIVTWGQSHVSTLHSGEEVDLYMDQSSGGGFESKDAYGSGLFEMRIKVPSGNTGGIVTAFYLTSKGGGHDEIDFEFLGNNNGKPVTLQTNLFLNGEGNREERFLLWFNPTKHYHTYGLLWNPYQIVFYVDNIPIRVYKNENGVSYPSKPMQVEASLWNGDDWATDGGRTKVNWSYSPFIAHFRDFALSGCNIDGRSNNVGACESSNYWWNAGNYQRLSGNEQKLYEHVRSKYMNYDYCTDRSKYQTPPRECY

>AtXTH31

MALSLIFLALLVLCPSSGHSQRSPSPGYYPSSRVPTSPFDREFRTLWGSQHQRREQDVVTLWLDKSTGSGFKSLRPYRSGYFGASIKLQPGFTAGVDTSLYLSNNQEHPGDHDEVDIEFLGTTPGKPYSLQTNVFVRGSGDRNVIGREMKFTLWFDPTQDFHHYAILWNPNQIVFFVDDVPIRTYNRKNEAIFPTRPMWVYGSIWDASDWATENGRIKADYRYQPFVAKYKNFKLAGCTADSSSSCRPPSPAPMRNRGLSRQQMAALTWAQRNFLVYNYCHDPKRDHTQTPEC

>AtXTH11

MRGSDQKILLMVMVVVAVVAAAQGQEETTGFVTWGNNYYQTWGHQALVINKTSELQLTLDKNSGSGFESQLIYGSGYFNVRIKAPQTTSTGVITSFYLISRSSRHDELCFQILGKNGPPYLLNTNMYLYGEGGKDQRFRLWFDPTKDYHSYSFLWNPNQLVFYVDDTPIRVYSKNPDVYYPSVQTMFLMGSVQNGSIIDPKQMPYIAKFQASKIEGCKTEFMGIDKCTDPKFWWNRKQLSSKEKTLYLNARKTYLDYDYCSDRQRYPKVPQECGSYT

>AtXTH9

MVGMDLFKCVMMIMVLVVSCGEAVSGAKFDELYRSSWAMDHCVNEGEVTKLKLDNYSGAGFESRSKYLFGKVSIQIKLVEGDSAGTVTAFYMSSDGPNHNEFDFEFLGNTTGEPYIVQTNIYVNGVGNREQRLNLWFDPTTEFHTYSILWSKRSVVFMVDETPIRVQKNLEEKGIPFAKDQAMGVYSSIWNADDWATQGGLVKTDWSHAPFVASYKEFQIDACEIPTTTDLSKCNGDQKFWWDEPTVSELSLHQNHQLIWVRANHMIYDYCFDATRFPVTPLECQHHRHL

>AtXTH1

MEYLSIFGFVSVLYLIIRVDARAYEVNGIDQSKVGFDDNYVVTWGQNNVLKLNQGKEVQLSLDHSSGSGFESKNHYESGFFQIRIKVPPKDTSGVVTAFYLTSKGNTHDEVDFEFLGNKEGKLAVQTNVFTNGKGNREQKLALWFDPSKDFHTYAILWNPYQIVLYVDNIPVRVFKNTTSQGMNYPSKPMQVVVSLWNGENWATDGGKSKINWSLAPFKANFQGFNNSGCFTNAEKNACGSSAYWWNTGSYSKLSDSEQKAYTNVRQKYMNYDYCSDKVRFHVPPSECKWNN

>AtXTH2

MNRIRYCFELVSVLFLMFTANARARGRGAIDFDVNYVVTWGQDHILKLNQGKEVQLSMDYSSGSGFESKSHYGSGFFQMRIKLPPRDSAGVVTAFYLTSKGDTHDEVDFEFLGNRQGKPIAIQTNVFSNGQGGREQKFVPWFDPTTSFHTYGILWNPYQIVFYVDKVPIRVFKNIKKSGVNYPSKPMQLVASLWNGENWATSGGKEKINWAYAPFKAQYQGFSDHGCHVNGQSNNANVCGSTRYWWNTRTYSQLSANEQKVMENVRAKYMTYDYCSDRPRYPVPPSECRWNQ

>AtXTH15

MGPSSSLTTIVATVLLVTLFGSAYASNFFDEFDLTWGDHRGKIFNGGNMLSLSLDQVSGSGFKSKKEYLFGRIDMQLKLVAGNSAGTVTAYYLSSQGATHDEIDFEFLGNETGKPYVLHTNVFAQGKGDREQQFYLWFDPTKNFHTYSIVWRPQHIIFLVDNLPIRVFNNAEKLGVPFPKSQPMRIYSSLWNADDWATRGGLVKTDWSKAPFTAYYRGFNAAACTASSGCDPKFKSSFGDGKLQVATELNAYGRRRLRWVQKYFMIYNYCSDLKRFPRGFPPECKKSRV

>AtXTH29

MRDSIYLLWIDNRLVVIIMMVMMVSCRCVLGLENINPIFFDEGLSHLFGEGNLIRSPDDRSVRLLLDKYTGSGFISSSMYQHGFFSSLIKLPGAYTAGIVVAFYTSNGDVFVKDHDELDIEFLGNLEGKPWRFQTNMYGNGSTNRGREERYRLWFDPSKEFHRYSILWTPTKIIFWVDDVPIREILRKEEMNGDYPQKPMSLYATIWDASSWATSGGKFGVDYTFSPFVSEFKDIALDGCNVSDSFPGENNNNNIGNYNNINCSVSDQFLMSNDYSTISPKQATAMRRFRERYMYYSYCYDTIRYSVPPPECVIVTAEKNRFRDTGRLKFGGSHPKVHKARKKRRRNRSTPVVSADL

>AtXTH23

MAMISYSTIVVALLASFMICSVSANFQRDVEITWGDGRGQITNNGDLLTLSLDKASGSGFQSKNEYLFGKIDMQIKLVAGNSAGTVTAYYLKSPGSTWDEIDFEFLGNLSGDPYTLHTNVFTQGKGDREQQFKLWFDPTSDFHTYSILWNPQRIIFSVDGTPIREFKNMESQGTLFPKNQPMRMYSSLWNAEEWATRGGLVKTDWSKAPFTASYRGFNEEACVVINGQSSCPNVSGQGSTGSWLSQELDSTGQEQMRWVQNNYMIYNYCTDAKRFPQGLPRECLAA

>AtXTH14

MACFATKQPLLLSLLLAIGFFVVAASAGNFYESFDITWGNGRANIFENGQLLTCTLDKVSGSGFQSKKEYLFGKIDMKLKLVAGNSAGTVTAYYLSSKGTAWDEIDFEFLGNRTGHPYTIHTNVFTGGKGDREMQFRLWFDPTADFHTYTVHWNPVNIIFLVDGIPIRVFKNNEKNGVAYPKNQPMRIYSSLWEADDWATEGGRVKIDWSNAPFKASYRNFNDQSSCSRTSSSKWVTCEPNSNSWMWTTLNPAQYGKMMWVQRDFMIYNYCTDFKRFPQGLPKECKL

>AtXTH26

MAGLQAKTLMFVLAAALATLGRTFVEADFSKNFIVTWGKDHMFMNGTNLRLVLDKSAGSAIKSKVAHLFGSVEMLIKLVPGNSAGTVAAYYLSSTGSTHDEIDFEFLGNATGQPYTIHTNLYAQGKGNREQQFRPWFNPTNGFHNYTIHWNPSEVVWFVDGTPIRVFRNYESEGIAYPNKQGMKVFASLWNAEDWATQGGRVKTNWTLAPFVAEGRRYKARACLWKGSVSIKQCVDPTIRSNWWTSPSFSQLTASQLTKMQKIRDGFMIYDYCKDTNRFKGVMPPECSKKQF

>AtXTH24

MSPFKIFFFTTLLVAAFSVSAADFNTDVNVAWGNGRGKILNNGQLLTLSLDKSSGSGFQSKTEYLFGKIDMQIKLVPGNSAGTVTTFYLKSEGSTWDEIDFEFLGNMSGDPYTLHTNVYTQGKGDKEQQFHLWFDPTANFHTYSILWNPQRIILTVDDTPIREFKNYESLGVLFPKNKPMRMYASLWNADDWATRGGLVKTDWSKAPFMASYRNIKIDSKPNSNWYTQEMDSTSQARLKWVQKNYMIYNYCTDHRRFPQGAPKECTTSS

>AtXTH18

MKLSCGTSFAFLIMFLFAAQSMHVYAGSFHKDVQIHWGDGRGKVRDRDGKLLSLSLDKSSGSGFQSNQEFLYGKAEVQMKLVPGNSAGTVTTFYLKSPGTTWDEIDFEFLGNLSGHPYTLHTNVYTKGSGDKEQQFHLWFDPTVNFHTYCITWNPQRIIFTVDGIPIREFKNSESIGVPFPTKQPMRLYASLWEAEHWATRGGLEKTDWSKAPFTAFYRNYNVEGCVWANGKSSCPANSSWFTQQLDSNGQTRMKGVQSKYMVYNYCNDKRRFPRGVPVECS

>AtXTH19

MKSFTFLILFLFAAQSISVYAGSFHKDVKIHWGDGRGKIHDNQGKLLSLSLDKSSGSGFQSNQEFLYGKAEVQMKLVPGNSAGTVTTFYLKSPGTTWDEIDFEFLGNISGHPYTLHTNVYTKGSGDKEQQFHLWFDPTANFHTYCITWNPQRIIFTVDGIPIREFMNAESRGVPFPTKQPMRLYASLWEAEHWATRGGLEKTDWSKAPFTAYYRNYNVEGCVWVNGKSVCPANSQWFTQKLDSNGQTRMKGVQSKYMVYNYCSDKKRFPRGVPPECS

>AtXTH7

MVVSLFSSRNVFYTLSLCLFAALYQPVMSRPAKFEDDFRIAWSDTHITQIDGGRAIQLKLDPSSGCGFASKKQYLFGRVSMKIKLIPGDSAGTVTAFYMNSDTDSVRDELDFEFLGNRSGQPYTVQTNVFAHGKGDREQRVNLWFDPSRDFHEYAISWNHLRIVFYVDNVPIRVYKNNEARKVPYPRFQPMGVYSTLWEADDWATRGGIEKINWSRAPFYAYYKDFDIEGCPVPGPADCPANSKNWWEGSAYHQLSPVEARSYRWVRVNHMVYDYCTDKSRFPVPPPECSAGI

>AtXTH5

MGRLSSTLCLTFLILATVAFGVPPKKSINVPFGRNYFPTWAFDHIKYLNGGSEVHLVLDKYTGTGFQSKGSYLFGHFSMHIKMVAGDSAGTVTAFYLSSQNSEHDEIDFEFLGNRTGQPYILQTNVFTGGAGNREQRINLWFDPSKDYHSYSVLWNMYQIVFFVDDVPIRVFKNSKDVGVKFPFNQPMKIYSSLWNADDWATRGGLEKTNWEKAPFVASYRGFHVDGCEASVNAKFCETQGKRWWDQKEFQDLDANQYKRLKWVRKRYTIYNYCTDRVRFPVPPPECRRDRDI

>AtXTH20

MVSFCGRRFAFLIIFLFAAQYERVYAGSFHKDVQIHWGDGRGKILDNVGNLLSLSLDKFSGSGFQSHQEFLYGKVEVQMKLVPGNSAGTVTTFYLKSPGTTWDEIDFEFLGNISGHPYTLHTNVYTKGTGDKEQQFHLWFDPTVDFHTYCIIWNPQRVIFTIDGIPIREFKNSEALGVPFPKHQPMRLYASLWEAEHWATRGGLEKTDWSKAPFTAFYRNYNVDACVWSNGKSSCSANSSWFTQVLDFKGKNRVKWAQRKYMVYNYCTDKKRFPQGAPPECS

>AtXTH12

MAAFATKQSPLLLASLLILIGVATGSFYDSFDITWGAGRANIFESGQLLTCTLDKTSGSGFQSKKEYLFGKIDMKIKLVPGNSAGTVTAYYLSSKGETWDEIDFEFLGNVTGQPYVIHTNVFTGGKGNREMQFYLWFDPTADFHTYTVLWNPLNIIFLVDGIPIRVFKNNEANGVAYPKSQPMKIYSSLWEADDWATQGGKVKTDWTNAPFSASYRSFNDVDCCSRTSIWNWVTCNANSNSWMWTTLNSNQLGQLKWVQKDYMIYNYCTDFKRFPQGLPTECNLN

>AtXTH13

MAAFTTKQSLLLLSLLLLISLSAGSFYDNFDITWGNGRANIVESGQLLTCTLDKISGSGFQSKKEYLFGKIDMKMKLVAGNSAGTVTAYYLSSKGETWDEIDFEFLGNVTGQPYVLHTNVFTGGKGNREMQFYLWFDPTADFHTYTVLWNPLNIIFLVDGIPIRVFKNNEANGVAYPKSQPMKIYSSLWEADDWATQGGKVKTDWTNAPFSASYKSFNDVDCCSRTSLLNWVTCNANSNSWMWTTLNSNQYGQMKWVQDDYMIYNYCTDFKRFPQGLPTECNLN

>AtXTH25

MDRSTFILSLLFTLTVSTTTLFSPVFAGTFDTEFDITWGDGRGKVLNNGELLTLSLDRASGSGFQTKKEYLFGKIDMQLKLVPGNSAGTVTAYYLKSKGDTWDEIDFEFLGNLTGDPYTMHTNVYTQGKGDREQQFHLWFDPTADFHTYSVLWNPHHIVFMVDDIPVREFKNLQHMGIQYPKLQPMRLYSSLWNADQWATRGGLVKTDWSKAPFTASYRNFRADACVSSGGRSSCPAGSPRWFSQRLDLTAEDKMRVVQRKYMIYNYCTDTKRFPQGFPKECRH

>AtXTH22

MAITYLLPLFLSLIITSSVSANFQRDVEITWGDGRGQIKNNGELLTLSLDKSSGSGFQSKNEYLFGKVSMQMKLVPGNSAGTVTTLYLKSPGTTWDEIDFEFLGNSSGEPYTLHTNVYTQGKGDKEQQFKLWFDPTANFHTYTILWNPQRIIFTVDGTPIREFKNMESLGTLFPKNKPMRMYSSLWNADDWATRGGLVKTDWSKAPFTASYRGFQQEACVWSNGKSSCPNASKQGTTTGSWLSQELDSTAQQRMRWVQRNYMIYNYCTDAKRFPQGLPKECLAA

>AtXTH6

MAKIYSPSFPGTLCLCIFTLLTLMFIRVSARPATFVEDFKAAWSESHIRQMEDGKAIQLVLDQSTGCGFASKRKYLFGRVSMKIKLIPGDSAGTVTAFYMNSDTATVRDELDFEFLGNRSGQPYSVQTNIFAHGKGDREQRVNLWFDPSMDYHTYTILWSHKHIVFYVDDVPIREYKNNEAKNIAYPTSQPMGVYSTLWEADDWATRGGLEKIDWSKAPFYAYYKDFDIEGCPVPGPTFCPSNPHNWWEGYAYQSLNAVEARRYRWVRVNHMVYDYCTDRSRFPVPPPECRA

>OsXTH1

MGSLGRRPWVGGLTAAMIFAVAVCGFCFSGASAAAAAPTFGDNFEITGAEDHVKTSADGQTWYLYLDNKTGVGFQTKERYLFGWFSMNLKLAGNDSAGVVTAYYMCSDVDAAPQRDELDFEFLGNRTGEPYIIQTNVYRSGVGGREMRHSLWFDPTADFHSYSILWNPKQIVFFVDKVPIREYRNSDKPNTFFPIMKPMYVFSSIWNADDWATRGGLEKTDWTKAPFISSYRDFTADACSWGTAAASPPSCAASTGNSWWDQPPAWALDAGQREDSAWVARNLVIYDYCDDRKRFPSPPEECLLRTTSS

>OsXTH2

MATTTAAAMVVAMSVLLLGGGEAAAPRKPVDVAFEKNYVPTWAEDHIHYVDGGREVQLYLDKSTGTGFQTRGSYLFGHFSMHIKLVAGDSAGTVTAFYLSSQNSEHDEIDFEFLGNRTGEPYILQTNVFSGGKGDREQRIYLWFDPTKDYHSYSVLWNLYMIAFFVDDTPIRVFKNSKDLGVRYPFNQPMKLYSSLWNADDWATRGGREKTDWSRAPFVASYRGFHVDGCEASAEARYCATQGARWWDQPEFRDLDADQYRRLAWVRKTHTIYNYCDDRERYPAMSPECHRDRDA

>OsXTH3

MASLAVVVVVVAVVCAAGVAAAGKFDDVVEPSWANDHVVYEGDLLKLRLDSSSGGGFASKSKFLYGKATADLKLVAGDSAGVVTAFYLSSGGDKHNEFDFEFLGNVTGEPYLVQTNLYIDGVGNREQRIDLWFDPTADFHTYAVLWNPSQVVFLVDDTPIRVYENKNATAAVKGHHRHAAAANGTSNATSAAASVPPFPSPQPMSVYSSIWNADDWATQGGRVKTDWSHAPFVATFRDVRVEGCAWAANATDSDAGEVARCTGSSWGKEGRYWWKEKDMEELTVHQNHQLVWARAHHLVYDYCVDTDRFPVQPPECAGR

>OsXTH4

MGQARAHLLASLWAFYLILAISMVTGDLTNDLDILWGNSKVFYDNSGKQTISLTLDRWTTSAFRSKSTYLFSRIDMDIKLVAGDSAGTVTTLYVSGQGMNRLLILVDDKLIRQIKNNLMYSVPYPTYQPMRVYGSIWNADDWATMGGRVKTDWSQAPFTAYFRNYRAIACPPQQSSPLCGQSSGNWFNQELDVTRKQQLQEVDANYKIYDYCTDTKRFKDNLPKECTIN

>OsXTH5

MGRLANWLDDRTSKSTPDYRIQAGSFLLKLWFRPIFIIFFMPTSSCQPMMSSSYTFQESKHTPQSAYSLRHFALAKAMGQPRAQLLPSMSMAALYLILATSPVISDMTDSLDMLWGNTQVLYDSTGHQIVSLSLDRWTTSAFRSKTKYLFARIDMDIKLVAKDSAGTVTTLYMITEGLWDIHDEIDLEFLGNTTGEPYTLHTNIYARGTGGREKQYRLWFDPTEDFHTYTIIWNPQMILILVDGTPIRQMKNQLRNDIPFPLYQPMRLYASIWDADDWATQGGRIKTDWSQAPFTAFFRNYQANACIPYKTAWICSQGSNDSSWFTQDLDEEGKQKLKDVDDNYKIYDYCTDSRRYPNGYPPECGSQ

>OsXTH6

MAPARAHHLLACLLASALLAAATPVTGGGLMTDQLEVLFGQTQLLNDSNGDQTIALTLDREMGSAFKSKTSYLFARIDMDIKLVADDSAGTVTTIYLISEKDWNTHDEIDLEFLGNVTGQPYTLHTNIFANGEGGREVQYRLWFDPTQDFHTYSVIWNPDEILILVDNMPIRQFKNHLDSGVPFPIYQPMRLFGCLWDADDWATEGGRIKTDWSQAPFVAYFRNYTADGCVPSSYAWVCGQGPASSSDWFDRGLDDVKQQQQLREAQDKYMIYNYCNDPERFPDGYPKECGLQ

>OsXTH7

MAPMPLASSSSKLCSLLILCLAFLAAVDRSTAGIFDEIELIWGASRTYFFMDGDSEALALSLDQSQGSCFRSREKYLYVQIDVEIKLIEGDSAGTVCTIYTISEGPWEIHDEIDLEFLGNVTGEPYTLHTNIFANGVGGREQQFRLWFDPTADYHTYSIVWNPKRILILVDGKAIRDFKNNEDQGVPFPTWQSMRTFGSLWSAEDWATQGGRVKTDWKQAPFVTYYRNYNVTWCRPSPGVAWCGDEPKDSTRFDLDANTLSDLQWVRSNSMIYNYCDDSVRFNATTLPKECTLQ

>OsXTH8

MAMPCSGERCRRVWWSAAAAVVAFFFVVFVAAAAAAATASMYDDVEVVWGGDHSFFFMDGDGDALALCLDETHGSGFRSRDAYLYARFDVDMMLVANNSAGTVTTLYLMPDDVPWEYHDEVDLEFLGNVTGEPYTLHTNIFANGVGGREQQFRLWFDPTADFHTYSIVWNPKHIIILVDGVPIRDYRNTAARGGPAFPTWQKMRAHGSLWNADDWATQGGRVKTDWSEAPFFAYYRGLRVTPCAPSPGVAWCGDEPPESPWFDQQEMDAAALSKARQEHLLYDYCEDTKRFKDTGLPVECTIN

>OsXTH9

MGFGSREMACALVALVLGLCCVGGARATGRIDEGLEVMWGDGRGSVSPDGQVMALSLDHTSGSGWRSKNTYLFARVDLQIKLVANNSAGTVTTCYFMSEGEWDIHDEVDLEFLGNVTGQPYTLHTNVFANGTGGKEQQFHLWFDPTTDFHTYSIVWTSQHILVLVDGTPIREMKNHADKGIAYPSSQRMRLYGSLWNADDWATQGGRVKTDWSQAPFVARYRNFTATEAASSSSPAGYDQQMDATAQQAMKWARDNYMVYDYCADSKRFPQGFPPECSMP

>OsXTH10

MAALLVVVLVAMSAMVATANFNQEFDITWGDGRGKILEDGQLLTLTLDRTSGSGFQSKHEYLYGKIDMQLKLVPGNSAGTVTAYYLSSQGPTHDEIDFEFLGNVTGEPYTLHTNVFTQGQGQREMQFRLWYDPTKDFHTYSILWNPKHIIFMVDDMPIRDFRNLEGKGIAFPKNQPMRLYSSLWNADDWATQGGRVKTDWTHAPFSASYRGFRADACVVAAGGRTRCGATVGTDAAPGTGAAAAAGGWYNQELDLTRQQRMRWVQSKYMIYNYCTDPKRFPQGVPAECSM

>OsXTH11

MRTVALGIVAMACLVAIAHGGNFFQDAEVSWGQGRGKIVDGGRGLDLTLDRSSGSGFQSKSEYLFGKIDMQIKLVPGNSAGTVTTFYLSSQGSTHDEIDFEFLGNVTGEPYTLHTNVFTQGQGQREQQFRLWFDPTQSFHTYSIIWNPQHVIFAVDGTPIRDFKNHEARGVAFPKSQPMRVYASLWNADDWATQGGRVKADWSKAPFVASFRDFNADACVWSNGAQRCPVGTMETVAAPAGGRRGGAGGWWNQELSDMSYRRMRWVQRKFMIYNYCTDAKRFPQGTPAECKLR

>OsXTH12

MVSSSNPGRTPPLVAAIVCSVLLLAGGAAGNFYQDVDITWGDGRGKILGNGQLLTLSLDRSSGSGFQSKNQYLYGRFDMQIKLVPGNSAGTVATFYLSSQGSQHDEIDFEFLGNASGEPYTVHTNVYSQGKGGREQQFRMWFDPTKDFHTYSVLWNPSHILFYVDGTPIREYRNTEATTGVAFPRAQAMRVYASLWDAEEWATQGGRVRTDWSRAPFTASYRGLAASGCTSQDATACANPGSPWMYQQQLDSASQDRLRQVQRDYMIYNYCADTYRFPQGLPPECTAK

>OsXTH13

MAAKLQGGGGGAAVMAVVVVAMVAGAASGGNFYEECDATWEPQNCWSSDNGKSLSLALVSNSSGSMIRSKRQFVYGSVSTSVQLVPGNSAGTVTTFYTSSLGDKHDEIDFEFLGNETGQPYTIHTNVYANGVGDKEMQFKPWFDPTDGSHNYTISWTPCRIVWYIDGMPIRVFRNYQSSNGVAFPTWQPMYAYSSIWAAEDWATQKGRVKTDWSKAPFVANYHGIDLDVCECYGGDCVYGCAAAFNQGGGCAGQQLTGDEMGQMKWVQDNFRIYDYCVDYKRFNGQMAPECSLPQY

>OsXTH14

MGRLSLLLVVFTAAAAVVGLAGASFRDECDIPWEPQNARFTDDGNGLSLSLVSNYSGCMLRTKKQFIFGSVSTLIQLVPGNSAGTVTTYYTSSVGDNHDEIDFEFLGNETGQPYTIHTNIYANGVGDKEMQFKPWFNPTDGYHNYTVSWTACMIVWYIDGTPIRVFRNYEKSNGVAFPMKRPMYGYSSIWAAEDWATQGGRVKADWSKAPFVANYHGLNINVCECSTTSGGGNSCAAKCASTYNSKSSVCQLSDSELARMRKVQDEYRIYNYCVDPKRYNGSVPVECSLPQ

>OsXTH15

MAKALLAVVVVAVAAVLELGLVGANFQDQCDITWEPQNAKMTEGGDHLTLSLVSNSSGCMLRTKKQFIYGSVSTRIQLVKGNSAGTVTTYYTSSIGDKHDEIDFEFLGNSSGLPYTFHTNVFADGVGSREMQFRPWFDPTDGYHNYTIFWNPCMIVSHWTDDSIERHKYFCFRWFVDSIPIRVFRNHEKEGVPFPTKRPMYAFSSIWAAEDWATQGGRVKTDWTKAPFVAEYRDIGLNICECPGSGSGSSSSFSSSSSSTSGDAEDPACAQRCATSDHWYAAEGLCQLSDKQLRQMKAVQLGYTIYDYCADAQAKGRPVPPECSMPQY

>OsXTH16

MARPLGQQQVGAAAALVIVVACCVVAGCSGARGRGFREEFDVIWGEDHVRVTDEDDAATRQVVALTLDQASGSGFQSKDQFLFGEFSMEMKLVPGESPGTVATFYLTSEGDAHDEIDFEFLGNVSGEPYVMHTNVFAQGRGNREQQFYLWFDPTADFHNYTILWNPLNIIFSVDGKAVRVFKNHEAAGVPYPSGQAMRVHASLWNGDFWATRGGQVKINWTAAPFVASYRTYAYSACAVPAAGGGGGGPCTSGQLPNSTSSPSTCDCGGAWMDRQLGADGERDVAWARANYMIYDYCGDQWRFPQGLPAECSLDQSSGHRT

>OsXTH17

MAKHLALSVAAAVAVSWLAASSAAAAGFYEKFDVVGAGDHVRVVSDDGKTQQVALTLDRSSGSGFTSKDTYLFGEFSVQMKLVGGNSAGTVTSFYLSSGEGDGHDEIDIEFMGNLSGNPYVMNTNVWANGDGKKEHQFYLWFDPTADFHTYKIIWNPQNIIFQVDDVPVRTFKKYDDLAYPQSKPMRLHATLWDGSYWATRHGDVKIDWSGAPFVVSYRGYSTNACVNNNPAGGWSSSWCPEGTSAWIHRELDGAELGTVAWAERNYMSYNYCADGWRFPQGFPAECYRK

>OsXTH18

MRGGASLRLRWPAALVAVVAAAVTAAAAAGHGDHNFHRDFDAVWGKGNARFRDGGRMVELTLDEQTGARLQSKERFLFGRFDLEIKLVRGESAGTITSFYICSGGARHDEVDFEFLGNVSGEPYLLHTNIFSDGKGEREQQFVLWFDPTADFHTYSILWNPHNIILYIDGTPIRVFKNNEAYGVPFPTRQPVHVFASIWNAEEWATQGGRVKTDWSRAPFVATYRRYNVSNACVWDAAGAGASRCAGGGGGWMRRRMDWWSWMTLNWVRMNYMAYDYCADRKRFPHRFPAECIIPIGRT

>OsXTH19

MEQKPPAVAANNNQLLLMMIMVVVACSNYMISGAGAQPSPGYYPSKTIRSMAFGEGYDNLWGGQHQTLSADQTALTVWMDRSSGSGFKSKRSYRNGYFGASIKVPSGYTAGVNTAFYLSNNELYPGQHDEIDIELLGTVPGEPWTLQTNVYVHGTGDGAIIGREMRFHLWFDPTADFHHYAILWNPDHIVFLVDDVPVRRYPRAAGNTFPDRQMWAYGSIWDASDWATDGGRYKSDYRYQPFVSRYRDLKIAGCEAAAPASCQPVPASPSGATGELSAQQKAAMRWAQQRSMVYYYCQDYSRNHANYPEC

>OsXTH20

MARPGSGNIPGSACIPLLILLLLLLLLHPSEAQPSPGYYPSKMFRSMAFYEGYSTLWGPQHQTLSQDQKSLTLWMDRSSGSGFKSTRSYRNGYFGASIRVQPGYTAGVNTAFYLSNTEQYPGHHDEIDMELLGTVPGEPYTLQTNVYVRGSGDGNIVGREMRFHLWFDPTAGFHHYAILWNPDQILFLVDDVPIRRYEKKVEGTFPEREMWAYGSIWDASDWATDGGRYRADYRYQPFVSRFADLKVGGCATAAPPACSPVPASSGGGSAALSPQQEAAMAWAQRNAMVYYYCQDYSRDHTFYPEC

>OsXTH21

MAASAAAPATVAGLLVAVAAIMAASPAGAQPSPGYYPSSVHRAMAFSRDYTNKWGPQHQTLSADQSSLTIWLDKTCGSGFKSRKSYRNGYFAARVKLPAGYTAGTNTAFYLSNNEAHPGFHDEIDMEFLGTIPGEPYTLQTNVYVRGSGDGRIVGREMRFHLWFDPTADFHHYAILWNPDAITFFVDDVPIRRYERKSELTFPDRPMWVYGSIWDASDWATDDGRHRADYRYQPFVARFDRFTVAGCAPSAPASCRPVPASPAGAGLTPRQYAAMRWAQQSHMVYYYCQDYRRDHSLTPEC

>OsXTH22

MAIIGRRQQQGVAAAAATLVALMAVVVAAAAEAQPSPGVYPSRMFRAREFGRDFRSLWGAEHQQQEAAAPETGVTVWLDRRSGSGFKSRRAYRSGYFGAWVRLQRGYTAGVITAFYLSNGEAHPGWHDEVDMEFLGTTPGKPYTLQTNVFSLGSGDPPRSLGREIKFHLWFDPTADFHHYAILWTSDHIIFLVDDVPIRRYGRRSAGGAAGFPARPMWVYGSIWDASSWATEDGRYRADYSYQPFVARFSAFLLRGCSPHAPRTCAAPVAGDLTAAQLAAMRWAQRFHMVYNYCYDPKRDHSLTPECRTHLHPSSSSSNSSSSSYHG

>OsXTH23

MALEARFFLAAVFAVAATCLCLSAVASAFAVPSVAFDEGYSPLFGDDNLVRSSDDKSVRLLLDRRSGSGFISSDYYLHGFFSASIKLPKAYTAGVVVAFYLSNGDVYEKTHDELDFEFLGSRWGGQWRVQTNAYGNGSTARGREERYLLPFDPTLEAHRYSVLWAPTHIIFYIDDTPIREVIRHPGMGGDFPSKPMAVYATIWDGSTWATDGGKYKVNYKYAPFASEFSDLALLGCRADPVLRAPRDGGGAGCAEPDLLGLLTADYAVMTPRKRAAMRAFRARHMTYTVCYDAVRYAAGPFPECDVSDVEKESFSAWGESKNVVMKARGRGRRRGRKAGAGAMSRLDVSSS

>OsXTH24

MAAAVLAVLWACMMMMSLAPASLAASGFEEVPTIAFDEGFSPLFGEDNMVKSADGRTVSITLNRYTGSGFISSDYYHHGFFSASIKLPKDHTAGVVVAFYLSNGDVFEKTHDELDFEFLGNRYRHEWKMQTNVYGNGSTDRGREERYLMPFDPTADAHRFSILWHSRLIVFYVDGVPIREVPRTAAMGADYPSKPMALYVTIWDGSTWATDNGKYKVNYKRGPFTAVFSDLVLRGCTARSDIRLATTADDQDRCAAAEEDLMESDEYSSTMAMTARKRMAMRRFRQRQMLYTVCYDTNRYPEPFPECDVNMAERQMYWQWGESKVVRPRVRPRPGRRSKRRPSPEATAIPPPVLVSLQQAD

>OsXTH25

MTTSSWSGLLVISCMLLMSWAAAAVDMSPVRFDAAYMPLFGGDNLVPSPHARTVLLKLDRFTGSGFVSKSAYHHGFFSASIKLPHDYTAGVVVAFYLSNGDVFPGQHDELDFELLGNRRGHAWHVQTNMYGNGSTGRGREERYLLPFDPTAAPHSYAIAWTPAAVIFYIDAIPIRELVRCSSGDYPAKPMSVYATIWDGSAWATDGGRHKVDYAYAPFTAVFSDLVVTGGTDDDHCAAMGLMTSEVAVMTPAKRGSMRRFRSRHLTYSACYDTVRYNGTGVVFPECDESEQDNFHAWGESKRVINSRSSSSATYATGSGVRID

>OsXTH26

MAGRRLLVATAVVAAAAAVVAAAALEAINVTTVAFEEGYTPLFGFDNILRSADDRTVSLLLDRSTGSGFMSSSMYQHGFFSASIKLPSDYTAGVVVAFYTSNGDVIEKRHDELDFEFLGNIRGKPWRVQTNVYGNGSVSRGREERYLLPFDPTTEFHRYSILWTRAAIVFFVDDVPIREVRRTPAMTGDFPSKPMSIYATVWDASTWATSGGRYRVNYRYGPFVASFTDLALLGCRVGDPIGQMLSSAACTAAEDALLASDLAVMTLEKQQAMRRFREQNMVYSYCYDTLRYPAPFLECDVVESERRRFKGSGHLRLAFRRRRRTRPGSRPARPTRAADM

>OsXTH27

MAASRCFLLLLLLLLSPLLASAGEEEEEAVLAMAARLRRPAAASFREGYTQLFGDSNLALHGDGKRVRISLDERTGAGFASQDAYLHGFFSASIKLPPDYAAGVVVAFYMSNGDVYEKTHDELDFEFLGNIKGREWRVQTNVYGNGSTSVGREERYGLWFDPTEDFHRYAILWSHDWIVFYIDETPIREVQRTKSMGVQFPSKPMSLYATIWDGSSWATSGGRYKVNYKYAPFVAEFSELMLHGCAMDTLTRAPMCTPDIANIHNAVAMSGRQRSAMERFRTKYMTYGYCYDRLRYPTPPSECNVGPEAELFLPTGEARSIDRHGRARRHRRGPADSAF

>OsXTH28

MAMARCSLLPILAAVLLAASLSLPPRAAAYAAMVDSLLPASATALSFEEGYTQLFGDSNLMLHGDGKRVHISLDERTGAGFASQGAYHHGFFSASIKLPADHTAGVVVAFYMSNGDVYERTHDELDFEFLGNVRGREWRVQTNVYGNGSTAAGREERYGLWFDPTQDFHRYAIRWSHDTIIFYVDETPIREVVRTASMGAQFPSKPMSLYATIWDGSSWATSGGRYKVNYKYAPYVAEFTDLLLHGCPAGSPPPCEGAAASATMPPGQRSAMERFRARHMTYGYCYDRVRYHAPLPECSVGAEAEAFLPSGEARSTDRRGGRHGKRHRRAGGGVDSAL

>OsXTH29

MVVVVAMPPALSLLVLLVLALHGGAGDATPPPPLRLVRGARRVAFDEGYTRMFGDGNLAVLRDGRRVRLTLDESTGAGFASQDVFLHGFFSAAVKLPAYYAAGVVVAFYLSNGDTYEKTHDEVDFEFLGNVRGREWRVQTNVYGNGSTAAGREERYDLPFDPTDELHHYSILWTRRRIIFYVDETPIREVVRTAAMGAAFPAKPMSVYATIWDGSAWATLGGRYRVNYRYAPFVAEFADLVLHGCAVDPLAVEHSASCGDEEEEAAEAVVSSAAMAAFRRGHMSYSYCHDRRRYPVALSECALTGGAASLGRLFGPDGMKRRRARRARDASS
